# Quinazoline-tethered thiazolo[3,2-*a*]pyrimidines and their cyclic chalcone derivatives as HIV-1 reverse transcriptase inhibitors: green synthesis, molecular docking, and *in silico* studies

**DOI:** 10.1039/d6ra03750j

**Published:** 2026-09-28

**Authors:** Sobhi M. Gomha, Sami A. Al-Hussain, Yaser A. M. Sulaiman, Sayed M. Riyadh, Basant Farag, Mohammad Alhilal, Suzan Alhilal, Magdi E. A. Zaki

**Affiliations:** a Department of Chemistry, Faculty of Science, Islamic University of Madinah Madinah 42351 Saudi Arabia smgomha@iu.edu.sa; b Department of Chemistry, Faculty of Science, Imam Mohammad Ibn Saud Islamic University (IMSIU) Riyadh 11623 Saudi Arabia sahussain@imamu.edu.sa mezaki@imamu.edu.sa; c Department of Clinical Laboratory Sciences, College of Pharmacy-Tikrit University Iraq neimiyaser@tu.edu.iq; d Department of Chemistry, Faculty of Science, Cairo University Cairo 12613 Egypt riyadh1993@hotmail.com; e Department of Chemistry, Faculty of Science, Zagazig University Zagazig 44519 Egypt basantfarag@zu.edu.eg; f Department of Nursing, Faculty of Health Sciences, Mardin Artuklu University Mardin Turkey mohammadalhilal@artuklu.edu.tr; g Department of Medical Services and Techniques, Vocational School of Health Services, Mardin Artuklu University Mardin Turkey suzanalhilal@artuklu.edu.tr

## Abstract

Nitrogen-fused heterocycles have attracted considerable research interest in pharmaceutical, medicinal, organometallic, and materials chemistry. In this work, two new series of quinazoline-linked thiazolo[3,2-*a*]pyrimidines and their cyclic chalcone derivatives were synthesized using choline hydroxide as an environmentally friendly catalyst. The synthesized compounds were evaluated for their inhibitory activity against HIV-1 reverse transcriptase (RT). Compound 11e showed the highest inhibitory activity, with an IC_50_ value of 0.599 µg mL^−1^ (1.305 µM). Molecular docking was performed to examine the possible binding modes and interactions within the active site of the enzyme. However, only a weak correlation was observed between the docking scores and experimental IC_50_ values (Pearson's *r* ≈ −0.18), indicating that the docking results provide qualitative binding information rather than a direct prediction of the inhibitory activity. *In silico* ADMET analysis also indicated generally favorable oral drug-like properties. These findings suggest that the synthesized compounds, particularly 11e, may serve as preliminary leads for the further development of HIV-1 RT inhibitors.

## Abstract 2

Choline hydroxide-mediated green synthesis produced quinazoline-linked thiazolo[3,2-*a*]pyrimidines and cyclic chalcones with promising HIV-1 reverse transcriptase inhibition, supported by docking and* in silico *ADMET analysis.

## Graphical abstract

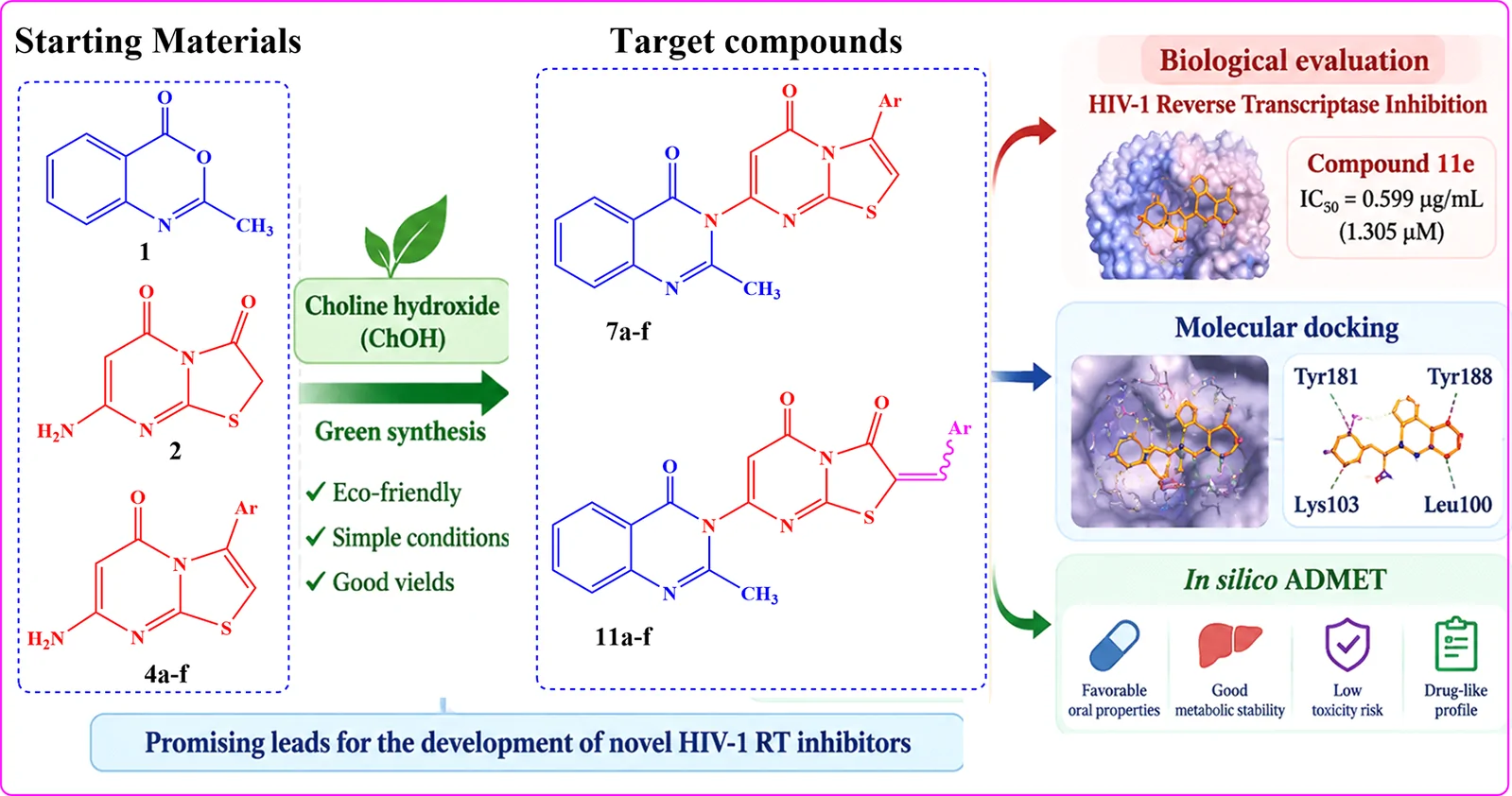

## 1. Introduction

Ribonuclease H (RNase H) is located within the subunit of reverse transcriptase (RT) and is considered a crucial enzyme in HIV replication. The significant challenges in their HIV inhibition applications are the high mutation rate and continuous formation of novel mutant strains. The current standard approach for managing HIV infection is antiretroviral therapy (ART).^1^ Therefore, the development of novel anti-HIV drugs with innovative mechanisms of action is a crucial target. Thiazolo[3,2-*a*] pyrimidines are popular motifs that serve as isosteric analogues of purines and were recently reported as allosteric inhibitors of RNase H with low cytotoxicity compared with active-site inhibitors.^2,3^ Also, these scaffolds have been implicated in a variety of therapeutic areas, including as 5-hydroxytryptamine (5-HT2A) receptor antagonists,^4^ inhibitors of cell division cycle 25B (CDC25B) phosphatase,^5^ xanthine oxidase inhibitors,^6^ tyrosinase inhibitors (a copper-containing enzyme that oxidizes tyrosine into melanin pigments in the skin, hair, and eyes),^7^ and inhibitors of the Bcl-2 family of proteins.^8^ The wide range of activities of thiazolo[3,2-*a*]pyrimidines as pharmacophores has revealed their potential as anticancer, anti-inflammatory, antimicrobial, antioxidant, anti-Alzheimer, antidiabetic, antitubercular, anticonvulsant, anti-Parkinsonian, and antimalarial agents.^9,10^

The conformationally restricted cyclic chalcones, with three-dimensional structures and different degrees of conjugation, are considered bioactive molecules capable of forming covalent and H-bond interactions. Recently, they have played a crucial role as follicle-stimulating hormone (FSH) agonists, peroxisome proliferator-activated receptor (PPAR) binders, cystic fibrosis transmembrane conductance regulator (CFTR) inhibitors,^11^ and anticancer agents.^12^ In materials science, the physicochemical properties of cyclic chalcones have been exploited in various applications, including high-contrast mechano-chromic luminescence,^13^ optical compounds,^14^ forensic reagents,^15^ photo-switches,^16^ semiconductors,^17^ anion sensors,^18^ and thermostable polymeric materials.^19^

Volatilization of hazardous chemicals has a detrimental effect on human health and the environment. Ionic liquids play a major role in addressing this global environmental problem and promoting the development of green chemistry.^19^ These ionic liquids have privileged properties, including high solubility, low volatility, biodegradability, and recyclability.^20,21^ An environmentally benign example of ionic liquids is choline hydroxide (ChOH), which possesses several advantageous properties, including a high basic character, ready availability, low cost, high polarity, and solubility in water and ethanol.^22^ Recently, ChOH was used as an alternative to conventional basic catalysts under mild and efficient conditions for the synthesis of fused heterocycles^23,24^ and thiazolidinone-based cyclic chalcones.^25^ Our on-going commitment to developing this technique with a sustainable profile directed our attention towards the synthesis of thiazolo[3,2-*a*]pyrimidines and their cyclic chalcone derivatives and the study of their biological activities against HIV-1 RT. The absorption, distribution, metabolism, excretion, and toxicity (ADMET) prediction of these compounds provided further motivation to investigate their *in silico* drug-likeness properties. This report continues our program aimed at the synthesis of bioactive candidates.^26–36^

## 2. Results and discussion

### 2.1. Chemistry

Our research aimed to prepare quinazoline as a framework skeleton from a benzoxazine-based molecule using an environmentally benign non-toxic catalyst. The ring opening of benzoxazine by a nitrogen nucleophile in the presence of a basic catalyst is a common procedure in the synthesis of quinazoline.^37–39^

The detailed synthetic route for the target compounds is summarized in Schemes 1–3. Scheme 1 outlines the preparation of quinazoline-tethered thiazolo[3,2-*a*]pyrimidine derivatives 5, 6, and 7a–f from benzoxazinone 1 and the corresponding heterocyclic amines under choline hydroxide-mediated conditions, including the substituent scope and isolated yields. Scheme 2 presents the proposed mechanism involving amine deprotonation, benzoxazine ring opening, cyclization, and dehydration. Scheme 3 describes the synthesis of cyclic chalcone derivatives 11a–f through the condensation of compound 5 with different aromatic aldehydes 10a–f, with reaction conditions and isolated yields included.

**Scheme 1:**
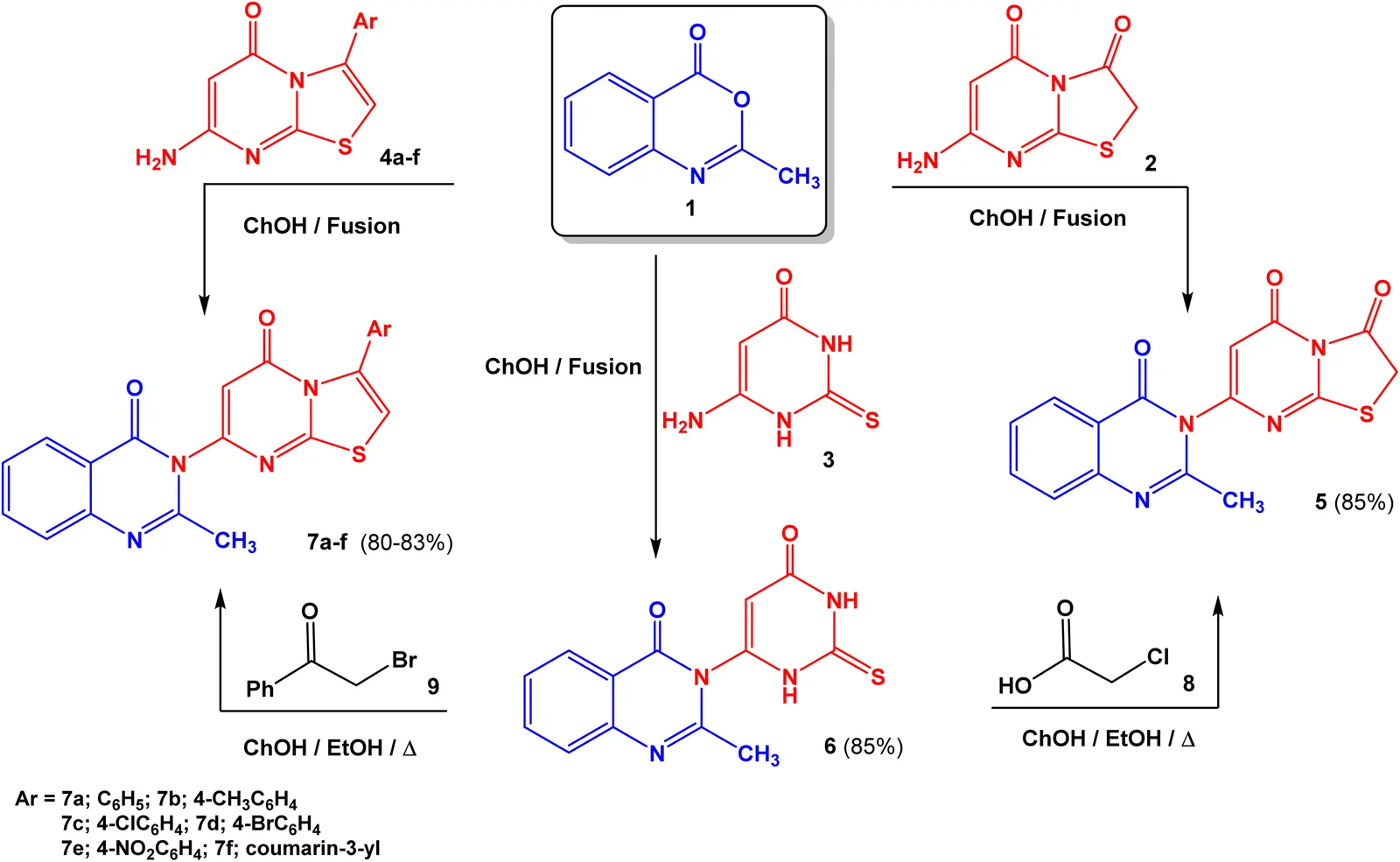
Detailed synthetic route of the quinazoline-tethered thiazolo[3,2-*a*]pyrimidine derivatives 5, 6, and 7a–f.

**Scheme 2:**
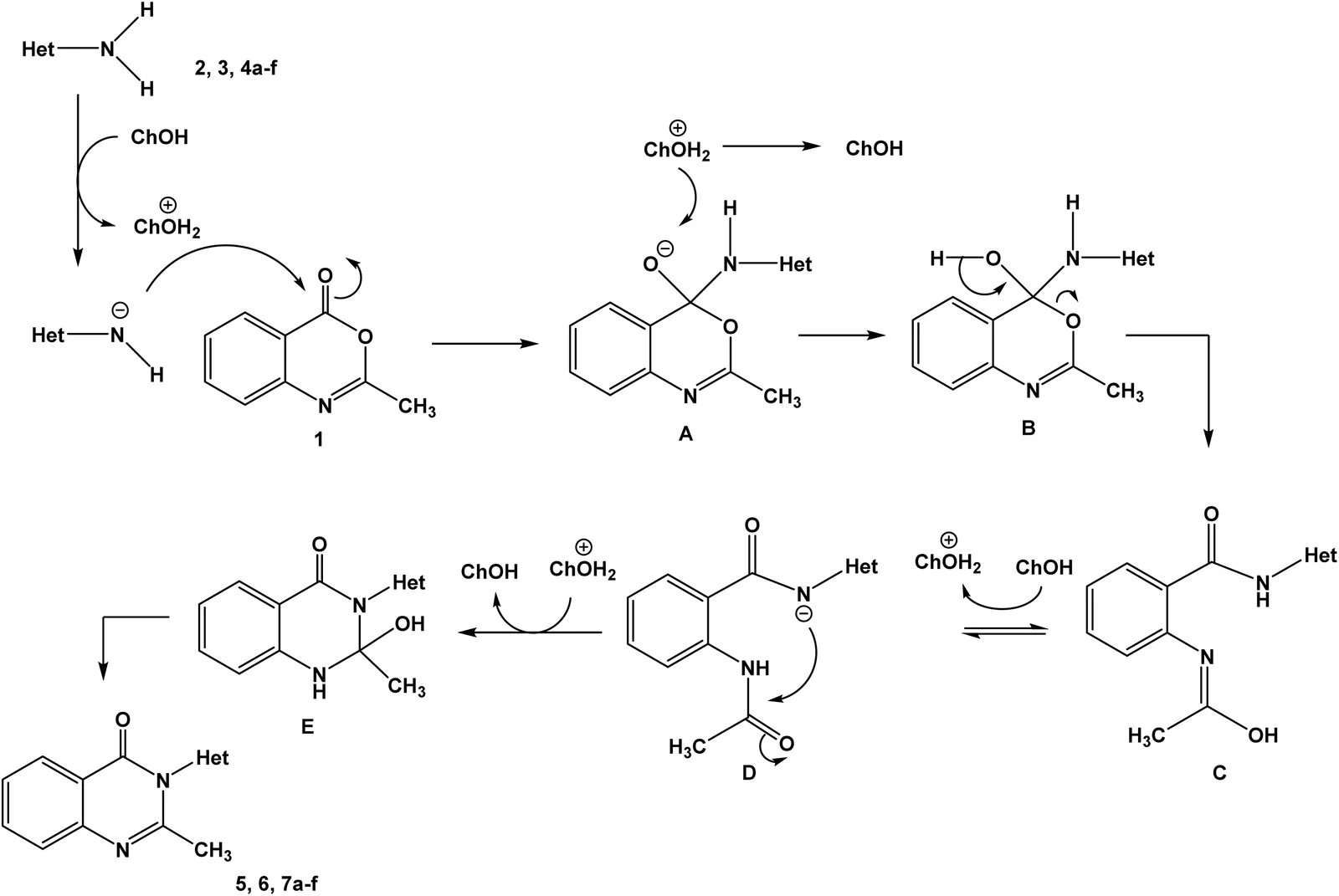
Proposed mechanism of the benzoxazine ring opening and quinazoline ring formation.

**Scheme 3:**
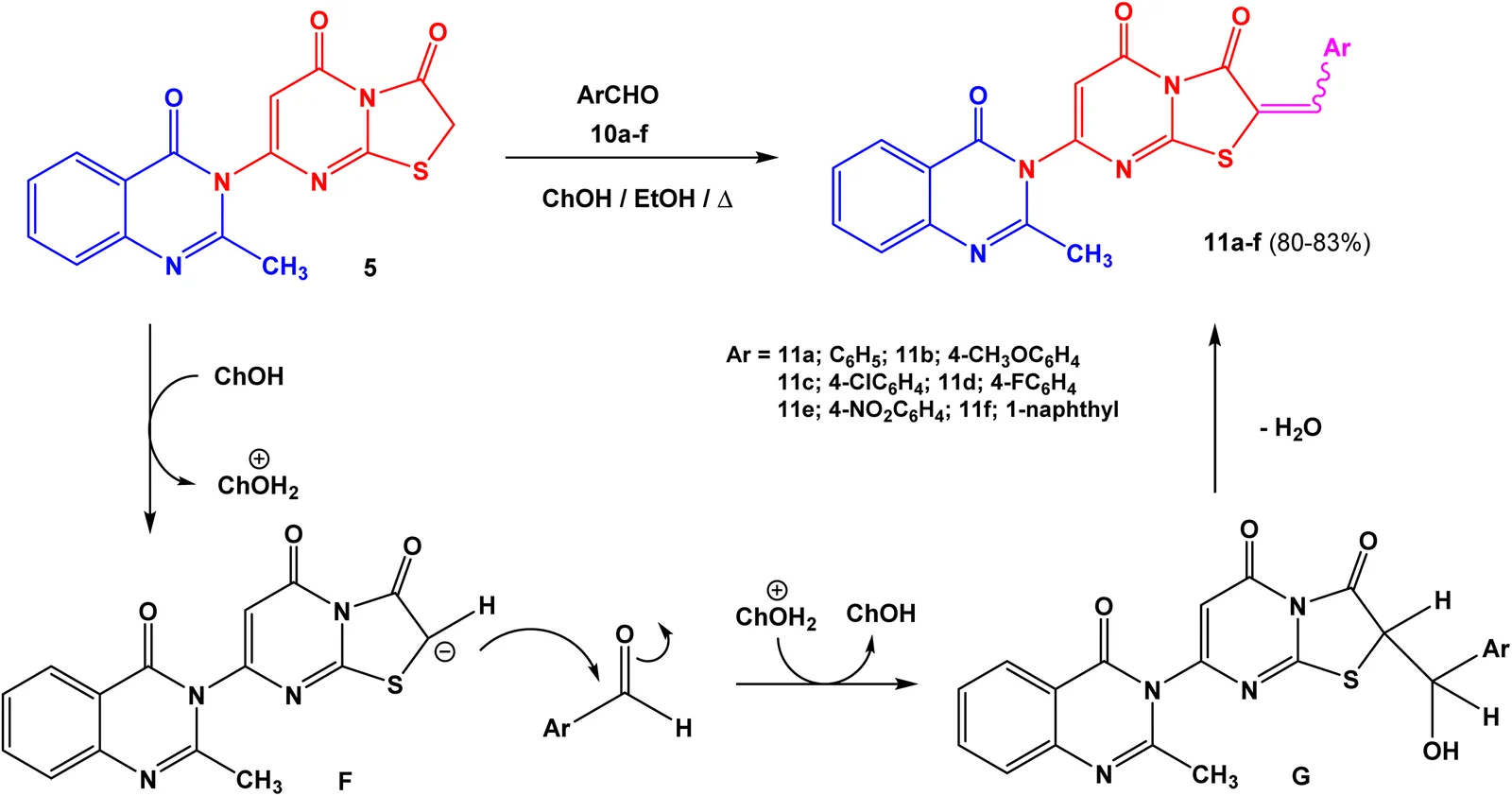
Detailed synthesis of the cyclic chalcone derivatives 11a–f.

The inherent synthetic strategy adopted in our study was initiated by fusing 2-methyl-4*H*-benzo[3,1]oxazin-4-one (1) with 7-amino-5*H*-thiazolo[3,2-*a*]pyrimidine-3,5(2*H*)-dione (2),^40^ 6-amino-2-thioxo-2,3-dihydropyrimidin-4(1*H*)-one (3) or 7-amino-3-aryl-5*H*-thiazolo[3,2-*a*]pyrimidin-5-ones (4a–f)^41–43^ in the presence of choline hydroxide (ChOH) under thermal conditions to furnish 7-(2-methyl-4-oxoquinazolin-3-yl)-5*H*-thiazolo[3,2-*a*]pyrimidine-3,5(2*H*)-dione (5), 2-methyl-3-(6-oxo-2-thioxo-1,2,3,6-tetrahydropyrimidin-4-yl)quinazolin-4(3*H*)-one (6) or 7-(2-methyl-4-oxoquinazolin-3-yl)-3-aryl-5*H*-thiazolo[3,2-*a*]pyrimidin-5-one (7a–f), respectively, as depicted in Scheme 1.

The molecular structures of compounds 5, 6, and 7a–f were confirmed based on their spectral data and elemental analyses. The IR spectra of the isolated products lacked the lactone carbonyl absorption characteristic of benzoxazine and revealed the lactam amide absorption of quinazoline at *υ* = 1682–1692 cm^−1^.^37^ The ^1^H NMR spectrum of compound 5 showed a singlet signal at *δ* = 3.87 ppm, integrating for 2 protons and assignable to the (CH_2_–CO) groups of the thiazolidinone ring. The pyrimidine-H at position 5 in compound 6 resonated at *δ* = 6.81 ppm, while the (NH–CO–NH–CS) residue resonated at *δ* = 10.82 and 11.54 ppm. Additionally, compounds 7a–f revealed a singlet signal at *δ* = 6.91–6.96 ppm, attributed to the (CH) of the thiazole ring.^35^

Alternative routes to compounds 5 and 7a involved treating compound 6 with chloroacetic acid (8) or phenacyl bromide (9), respectively, in an ethanolic solution of choline hydroxide under the same reaction conditions (Scheme 1).

The proposed mechanism for the ring opening of benzoxazine and ring closure to form quinazoline^39^ is depicted in Scheme 2. Choline hydroxide promotes the initial step of the reaction *via* deprotonation of the heterocyclic amine, thereby increasing its nucleophilicity. The resulting anion attacks the lactone group of benzoxazine and affords intermediate (A), which abstracts a proton from the oxonium ion of choline hydroxide and is converted into intermediate (B). Ring opening of the latter intermediate gives intermediate (C). Deprotonation and ring closure of intermediate (D) yield the respective intermediate (E), which undergoes dehydration to yield the isolated products 5, 6, and 7a–f (Scheme 2).

To demonstrate the generality of the above-mentioned environmentally benign procedure, we extended its scope to the preparation of cyclic chalcones. Thus, heating compound 5 with different aromatic aldehydes 10a–f in an ethanolic solution of choline hydroxide yielded the respective 2-arylidine-7-(2-methyl-4-oxoquinazolin-3-yl)-5*H*-thiazolo[3,2-*a*]pyrimidine-3,5(2*H*)-dione derivatives (11a–f), as shown in Scheme 3.

The consecutive steps of this condensation reaction are initiated by deprotonation of the active methylene group (CH_2_–CO) in the thiazolidinone ring with the aid of choline hydroxide, affording intermediate F. The resulting carbanion then attacks the aldehydic carbonyl group to form the corresponding tetrahedral addition intermediate G. Subsequent dehydration of this intermediate furnishes the isolated cyclic chalcones 11a–f (Scheme 3).^25^

Two different choline hydroxide-mediated conditions were used depending on the nature of the transformation. Fusion conditions were applied for the synthesis of compounds 5, 6, and 7a–f, since this step involves benzoxazine ring opening followed by quinazoline ring formation, which requires stronger thermal activation. This method afforded the target products in good isolated yields. In contrast, compounds 11a–f were obtained through condensation of the active methylene group of compound 5 with aromatic aldehydes under milder ethanolic reflux conditions. These conditions were more suitable for the chalcone-forming step because they gave good yields, avoided high-temperature fusion, and allowed easier work-up. Therefore, while fusion was necessary for the ring-opening/cyclization step, ethanolic reflux was the preferable condition for preparing the cyclic chalcone derivatives 11a–f.

Although the same catalyst, choline hydroxide, was used in both steps, the synthesis was carried out stepwise to allow the isolation and full structural characterization of intermediate 5 before aldehyde condensation. A one-pot protocol for the direct preparation of compounds 11a–f from benzoxazinone 1, amine 2, and aldehydes 10a–f would be synthetically attractive and may reduce the need for work-up and minimize waste generation. This approach will be investigated in future optimization studies.

The use of choline hydroxide provides several green-chemistry advantages, because it is a readily available, non-volatile, and strongly basic ionic liquid that can promote the reactions without using conventional inorganic bases. In the present protocol, the synthesis of compounds 5–7a–f was performed under fusion conditions, reducing the need for a reaction solvent, while compounds 11a–f were prepared in ethanol. The reactions also afforded good isolated yields, supporting the practical efficiency of the method. However, the greenness of the process should be considered cautiously, since the fusion step requires a relatively high temperature (230 °C) for 6 h, which increases energy demand, and some products required recrystallization from EtOH/DMF. Therefore, although choline hydroxide improves the sustainability profile compared with many conventional basic catalysts, further optimization is still needed to reduce energy consumption, minimize DMF use, evaluate catalyst recyclability, and assess waste generation and atom economy more quantitatively.

### 2.2. *In silico* docking study

To gain insights into molecular interactions and docking scores, the newly synthesized ligand molecules were subjected to molecular docking studies against HIV-1 reverse transcriptase.^44^ The docking results for the newly synthesized compounds are presented alongside those of the reference drug (zidovudine), as shown in Tables 1, 2, and S1.

**Table 1:** Docking scores (kcal mol^−1^), interactions of the docked complexes, and RMSD (Å) values of the compounds 7a–f, 11a–f, and zidovudine within the binding site of the target enzyme

Cpd	Docking score (kcal mol^−1^)	Docked complex interactions	Donor atom	Acceptor atom	RMSD (Å)
7a	−6.375	**H-bond**	S	—	1.073
		1 (THR 80)			
7b	−6.898	**H-bond**	C	—	1.115
		1 (ASP 30)			
		**Arene–H**			
		1 (ILE 47)			
		1 (GLY 48)			
7c	−6.428	**Arene–H**	—	—	1.310
		1 (ALA 28)			
7d	−6.422	**Arene–H**	—	—	1.144
		1 (ALA 28)			
7e	−6.776	**H-bond**	—	O	1.264
		1 (ASP 29)	—		
		1 (ASP 30)			
		**Arene–H**			
		1 (ILE 47)			
		1 (PRO 81)			
7f	−6.762	**H-bond**	—	O	1.270
		1 (ASP 29)			
		**Arene–H**			
		1 (ILE 47)			
11a	−6.732	**H-bond**	S	—	0.581
		1 (ASP 25)	C	—	
		1 (ASP 30)			
		**Arene–H**			
		1 (ALA 28)			
		1 (ASP 29)			
11b	−6.916	**H-bond**	S	—	1.079
		1 (THR 80)			
11c	−6.709	**Arene–H**	—	—	1.611
		1 (ALA 28)			
11d	−6.571	**H-bond**	—	O	1.158
		1 (ASP 29)	—		
		1 (ASP 30)			
		**Arene–H**			
		1 (ILE 47)			
		1 (GLY 48)			
11e	−6.815	**Arene–H**	—	—	1.110
		1 (ASP 29)			
		1 (GLY 49)			
11f	−6.838	**Arene–H**	—	—	1.474
		1 (ASP 29)			
Co-crystallized ligand	−5.144	**H-bond**	—	O	1.386
		1 (ASP 29)	O	—	
		1 (ASP 30)			
		**Arene–H**			
		1 (ALA 28)			
Zidovudine	−5.796	**H-bond**	N	—	1.712
		1 (ASP 30)	—	O	
		1 (ASP 30)			
		**Arene–H**			
		1 (ILE 47)			

**Table 2:** 2D and 3D ligand interactions of the ligands with 7a–f, 11a–f, and zidovudine at the 6DV4 binding site of the target enzyme

Cpd	2D ligand interactions	3D ligand interactions
7b	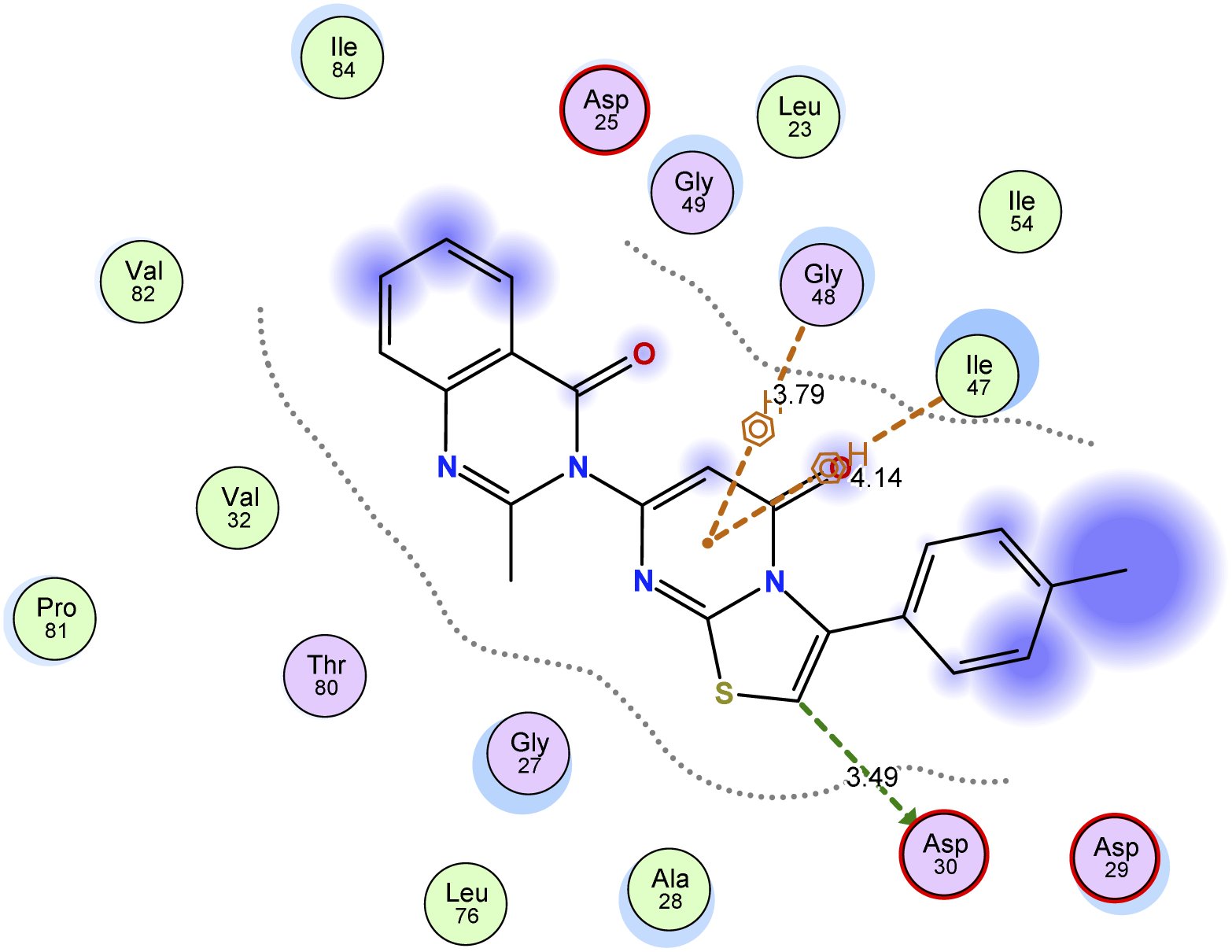	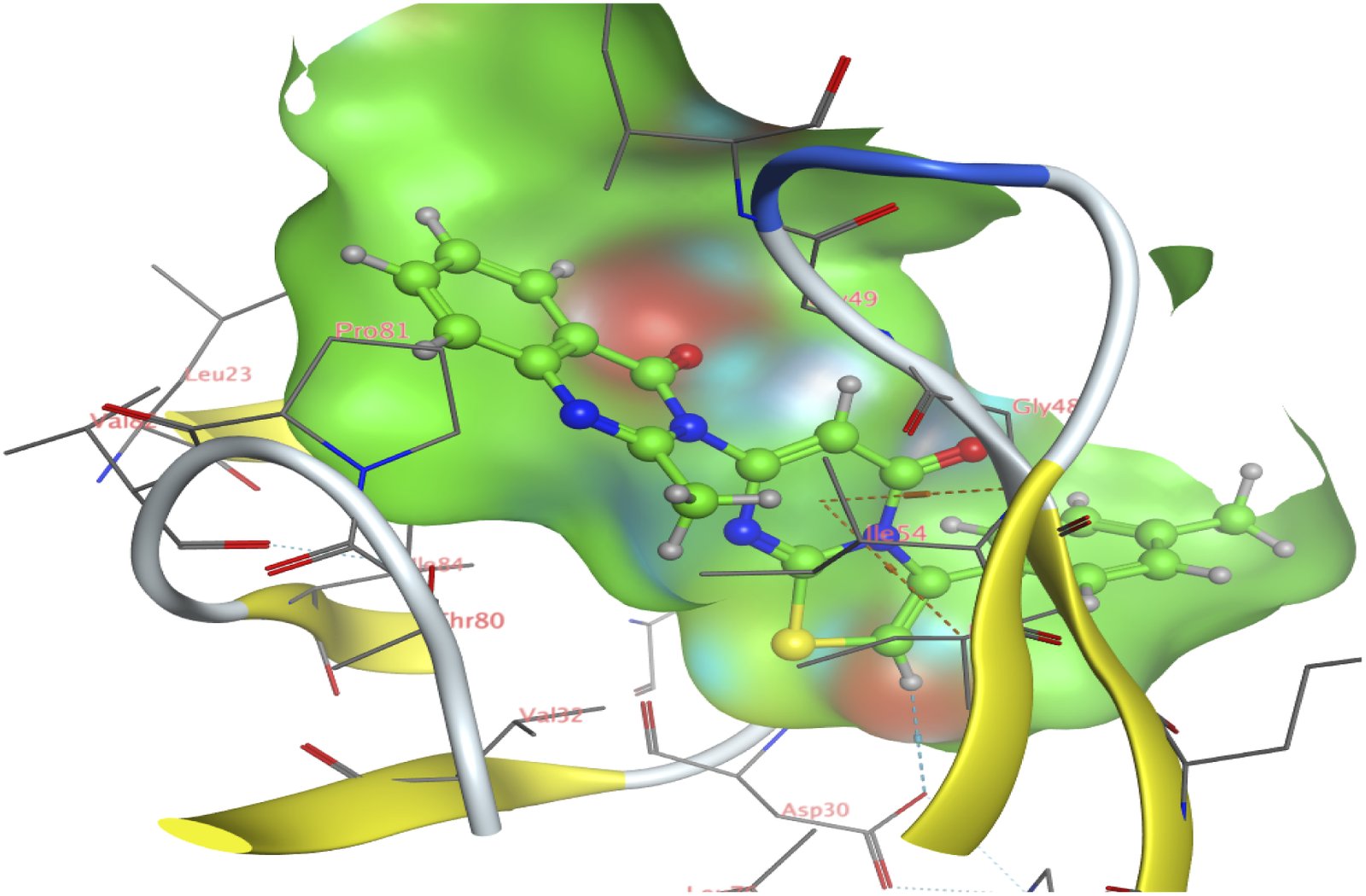
7e	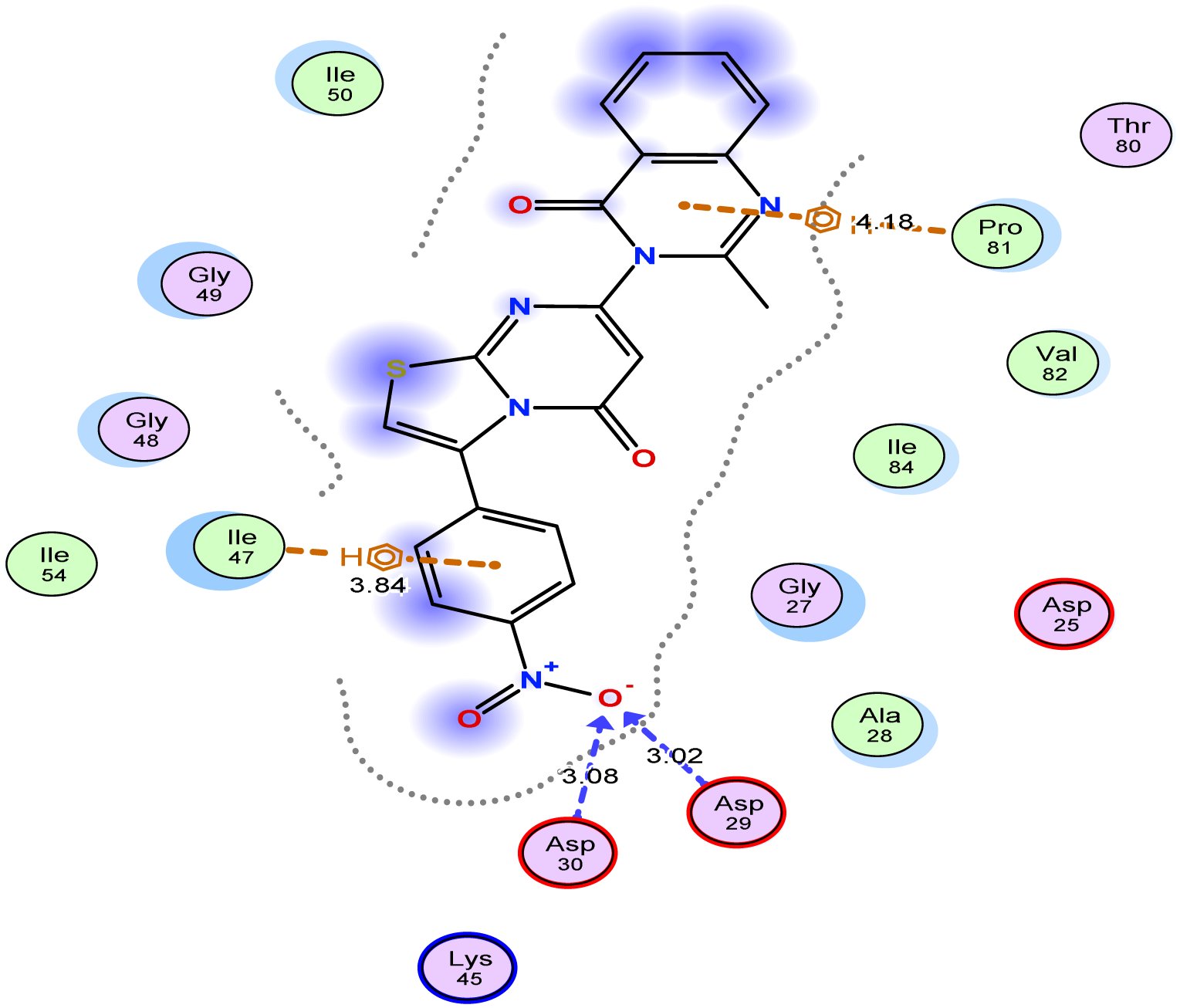	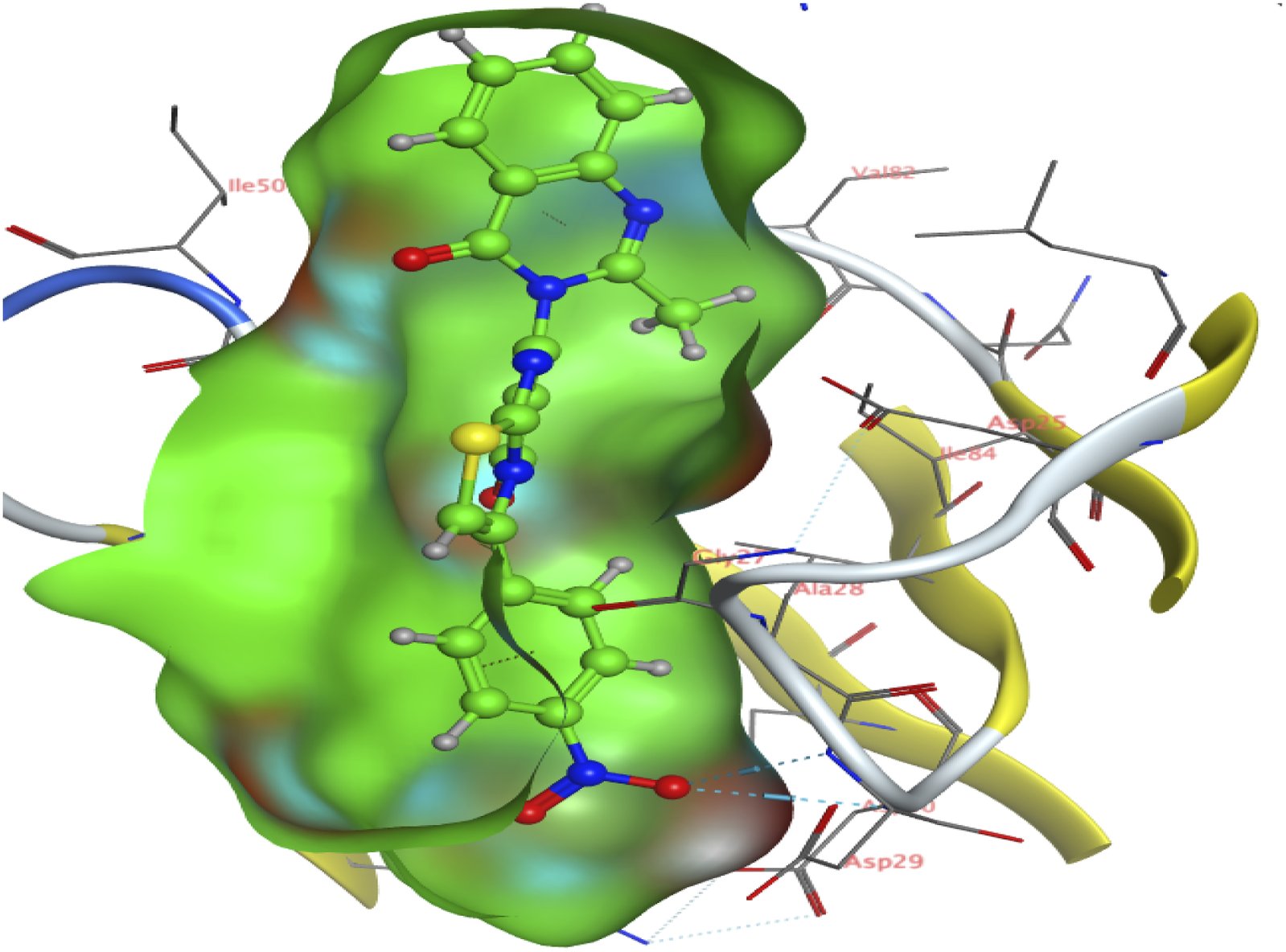
7f	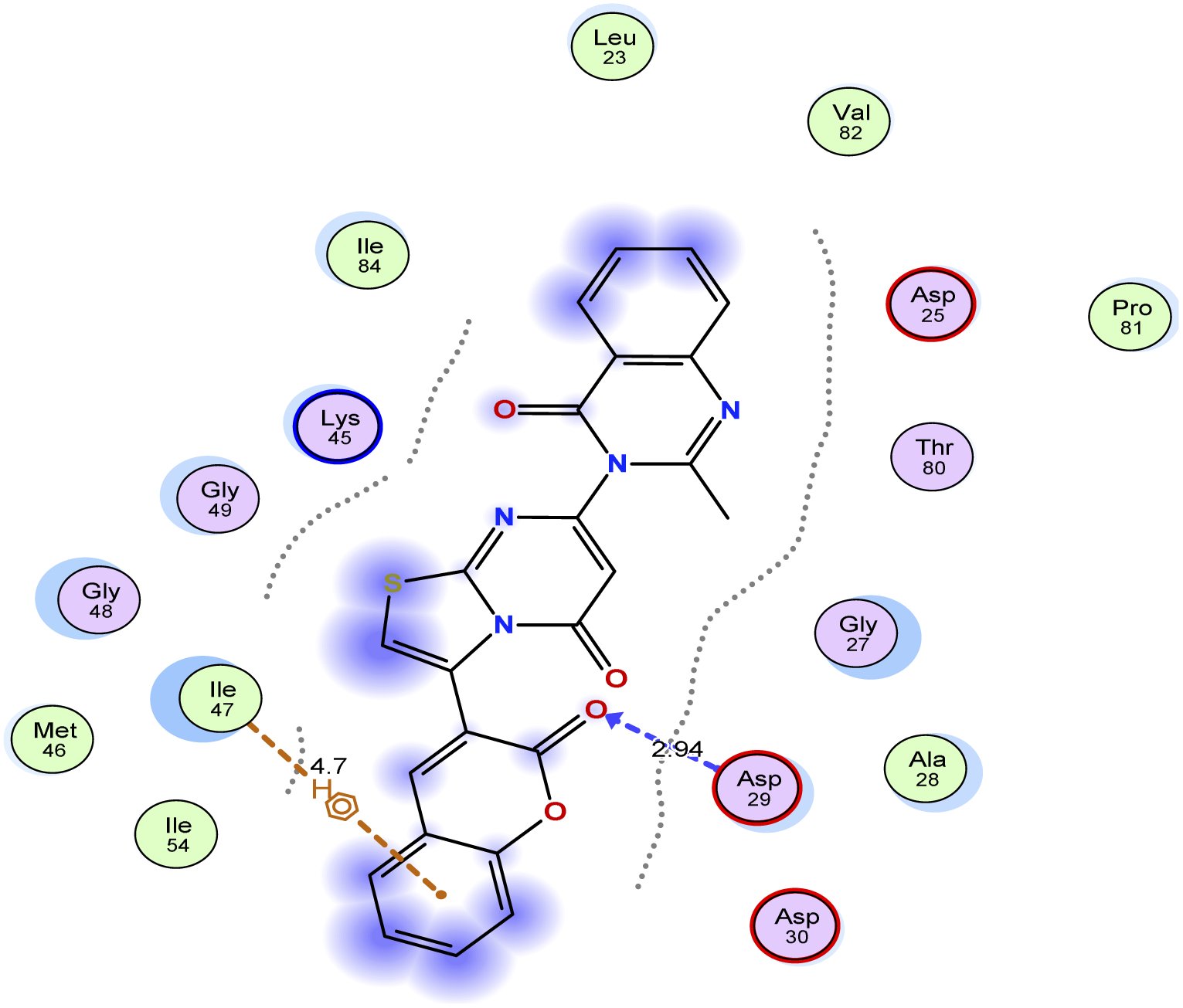	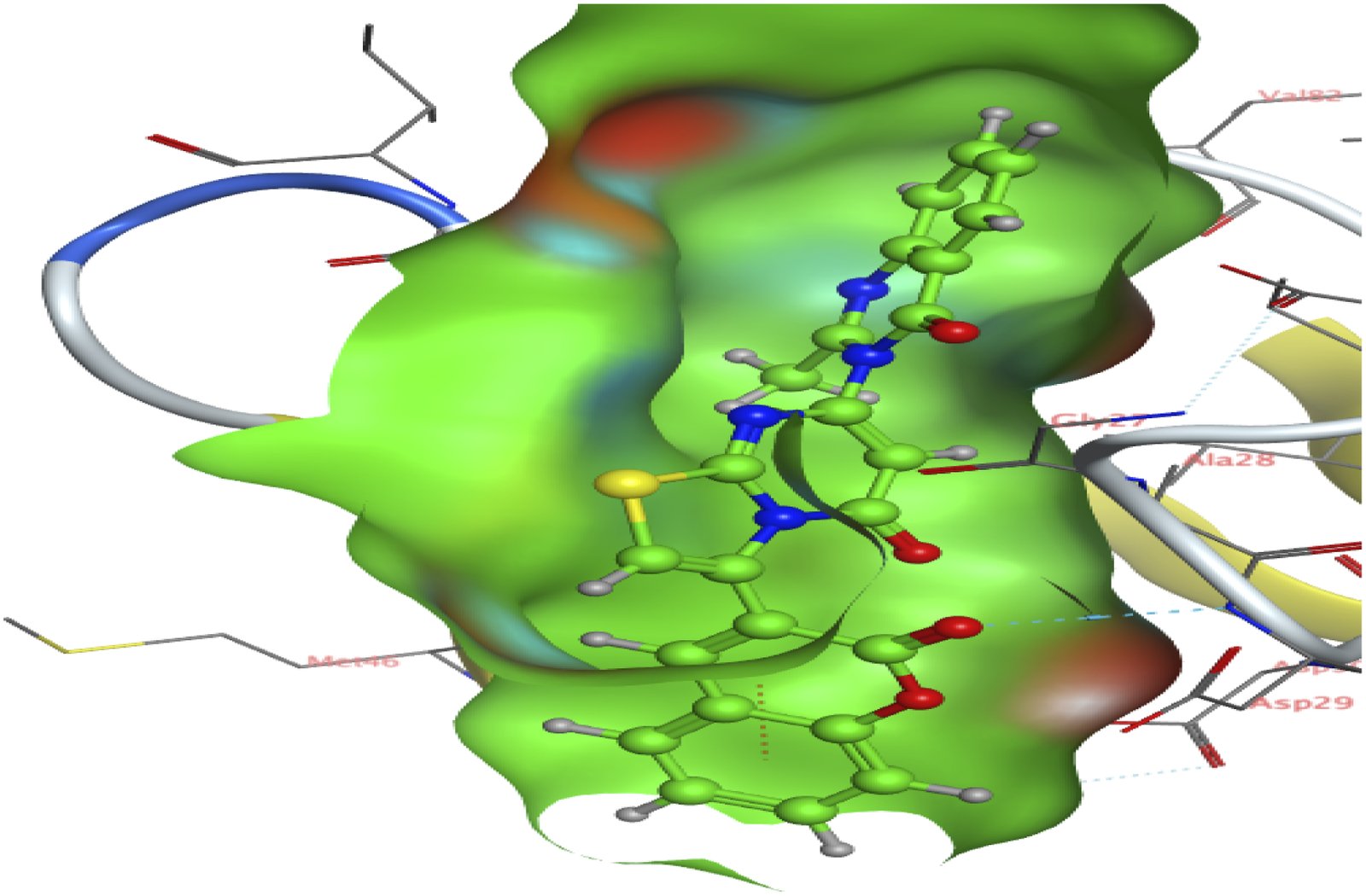
11b	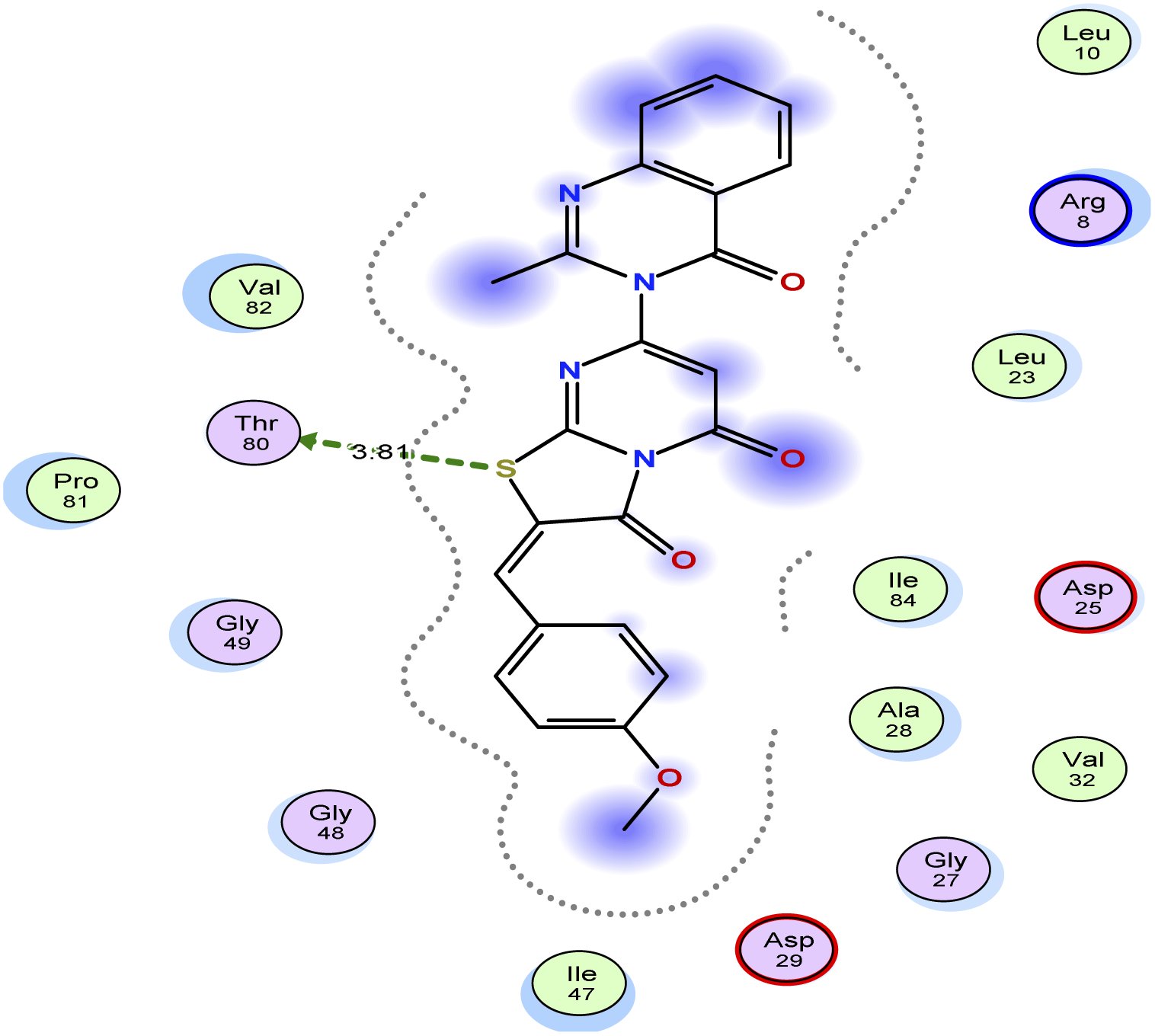	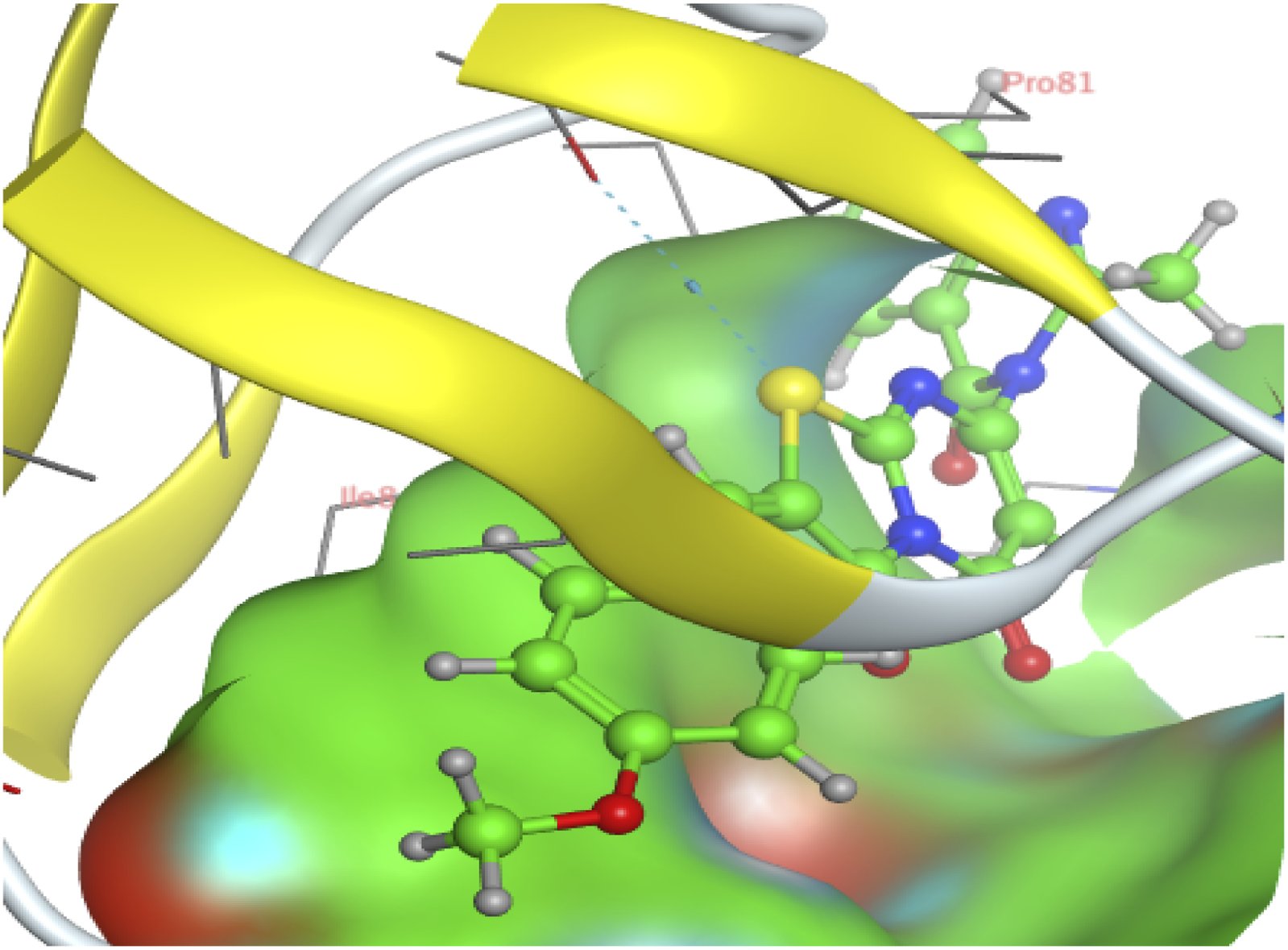
11e	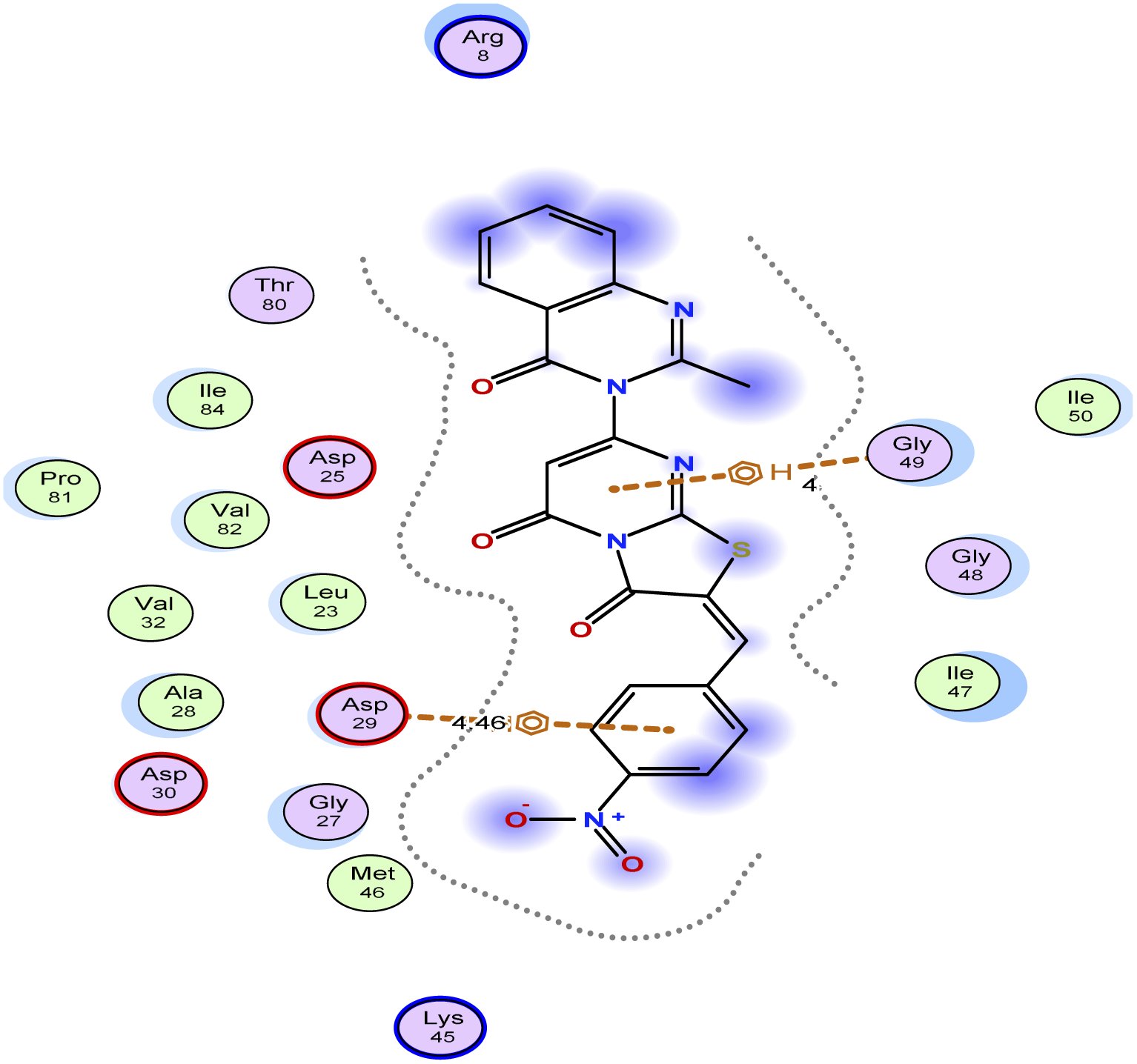	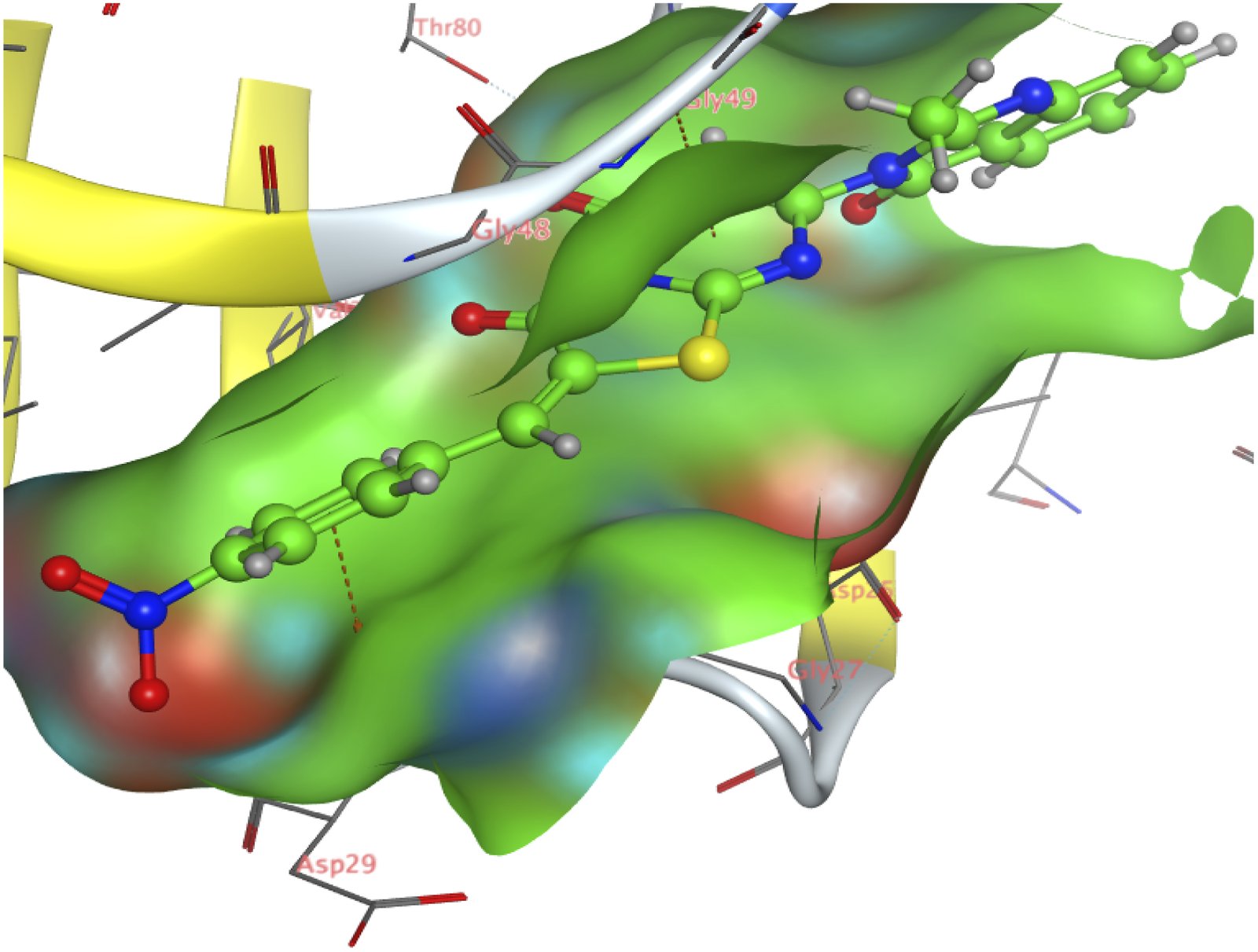
11f	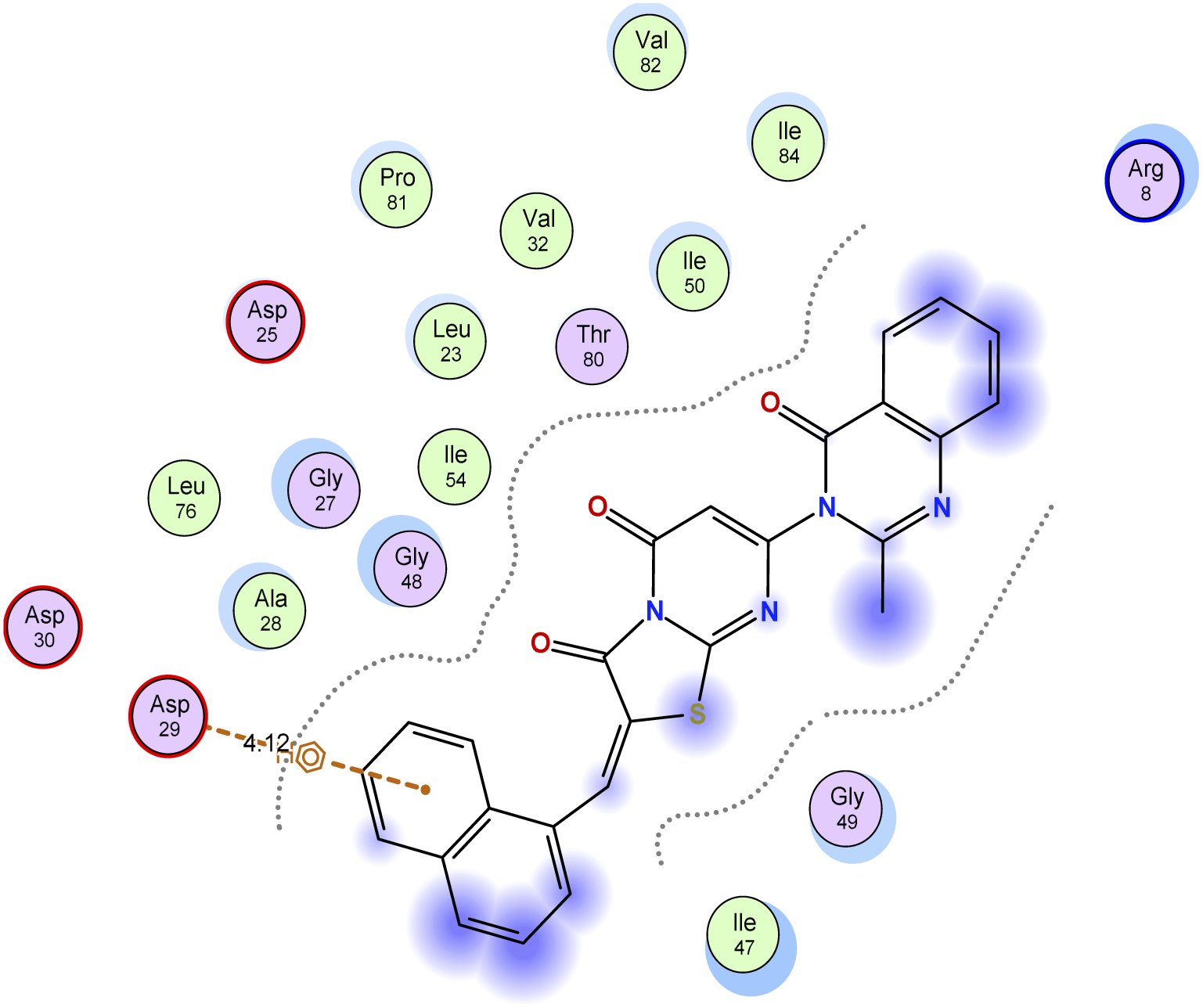	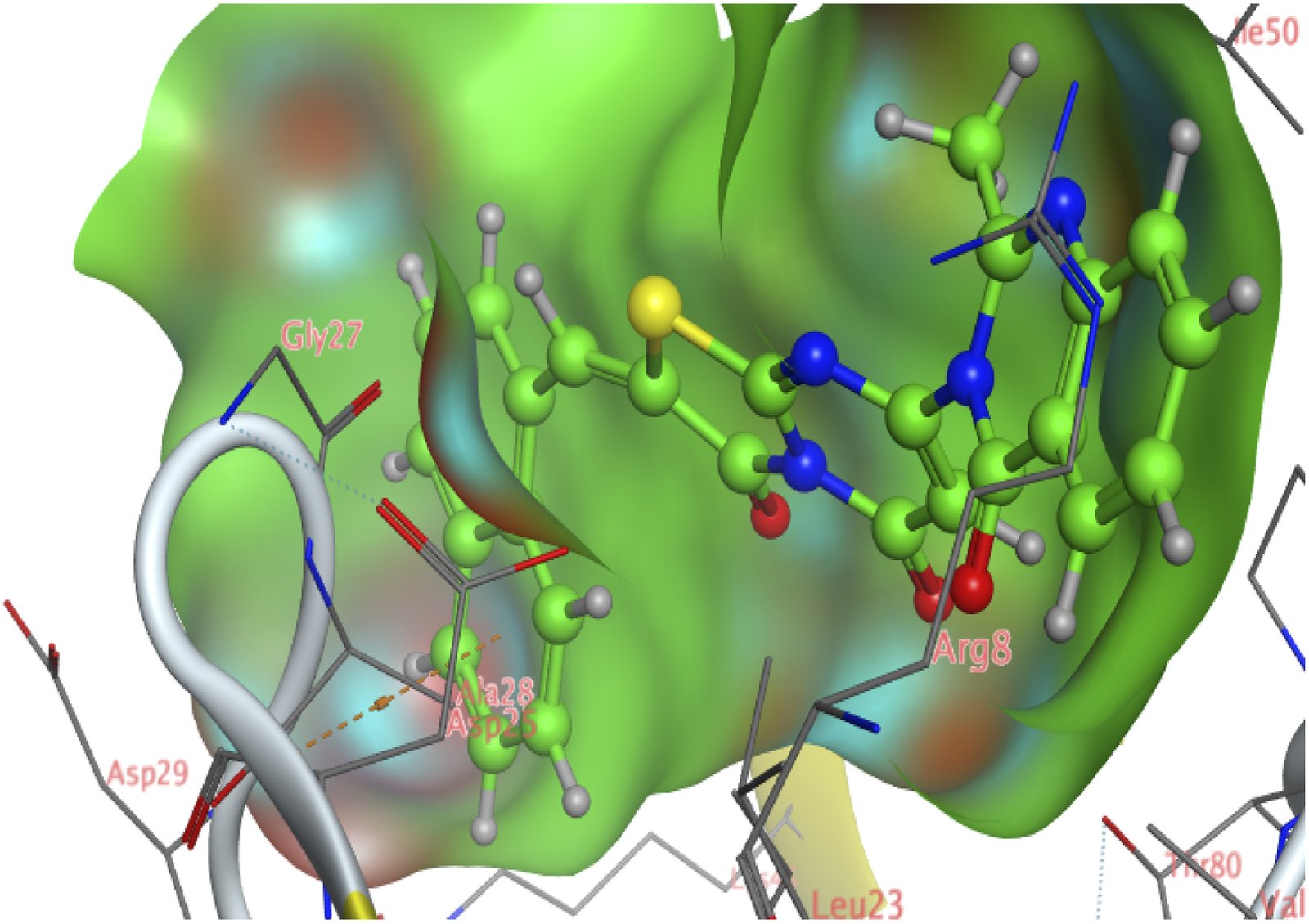
Co-crystallized ligand	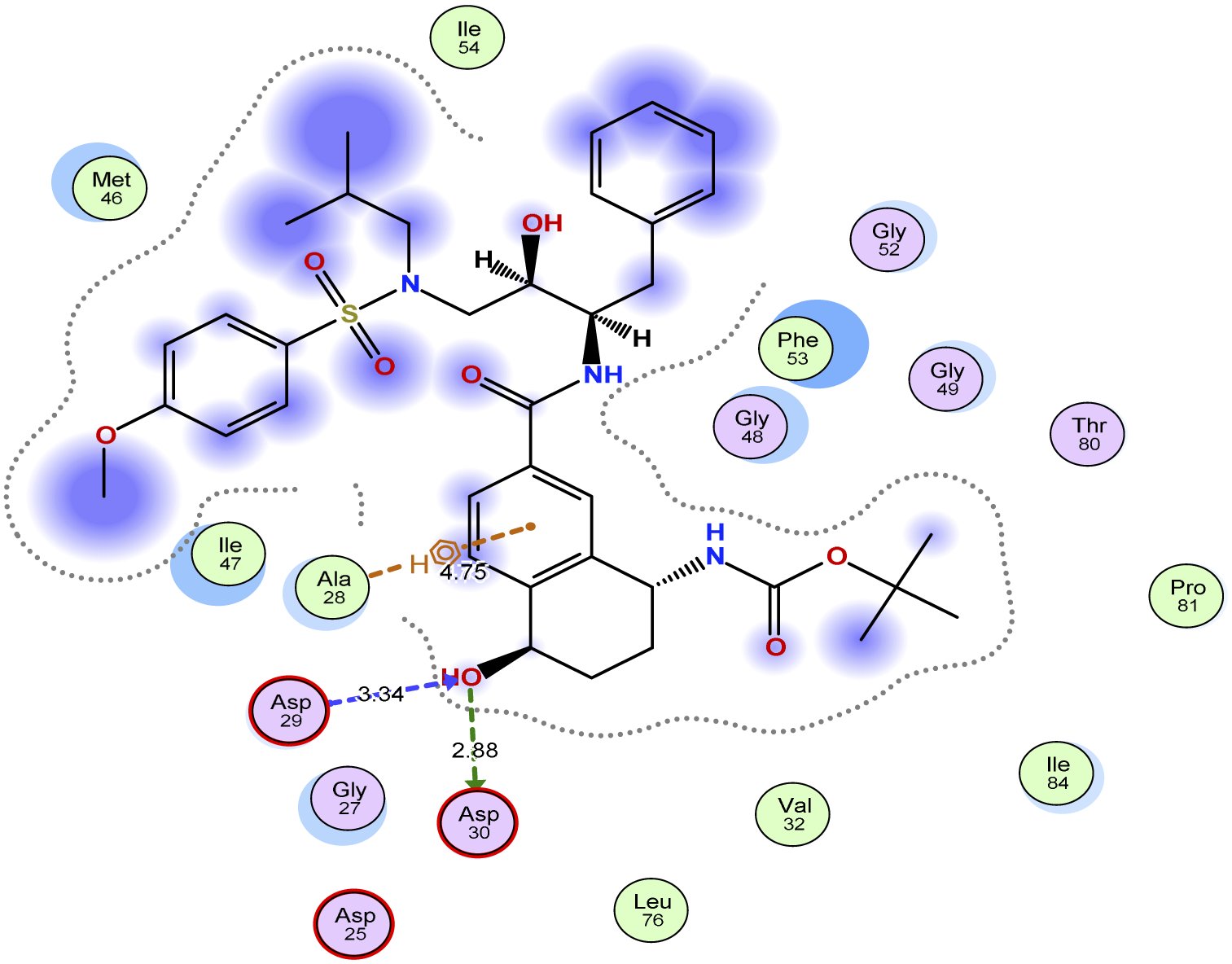	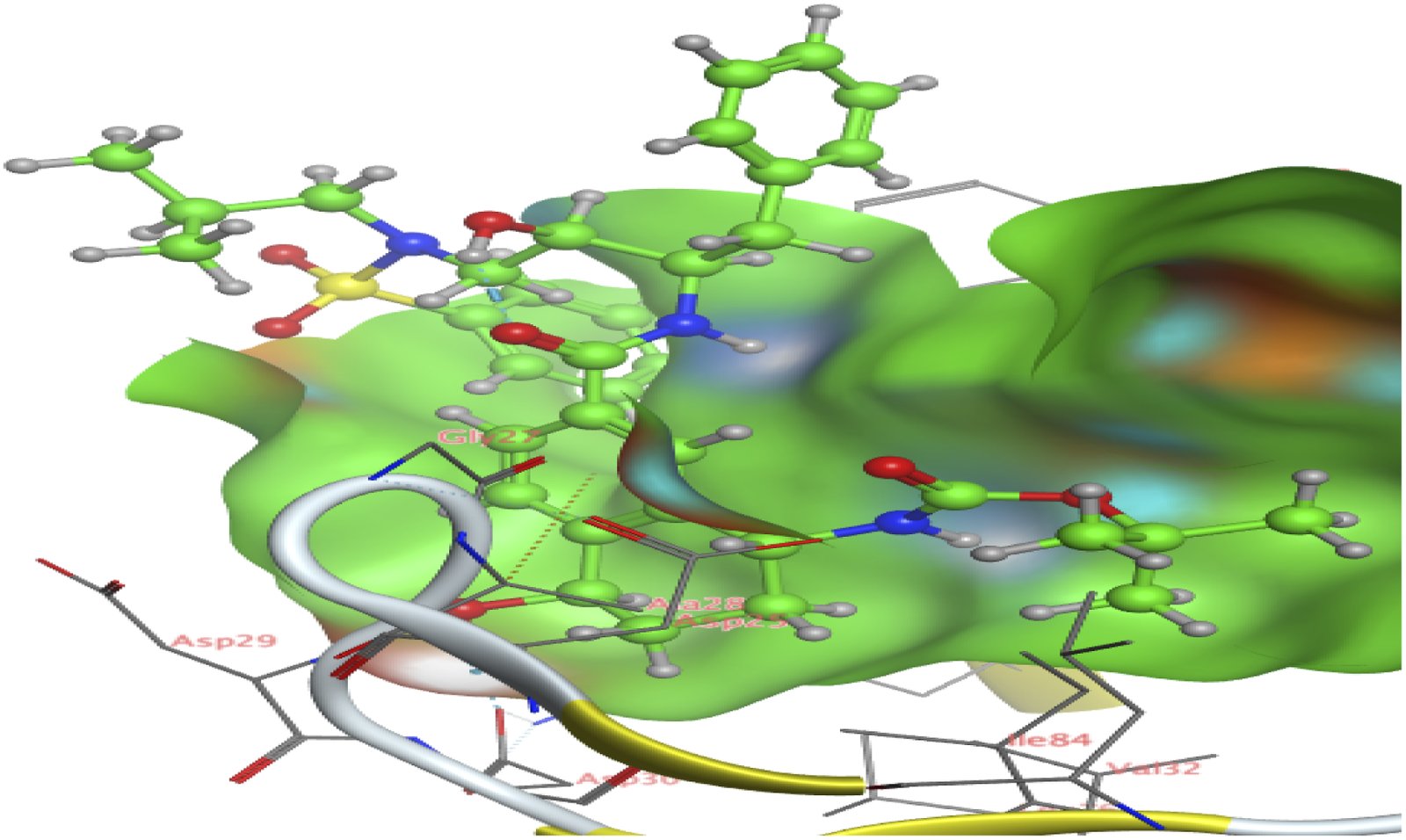
Zidovudine	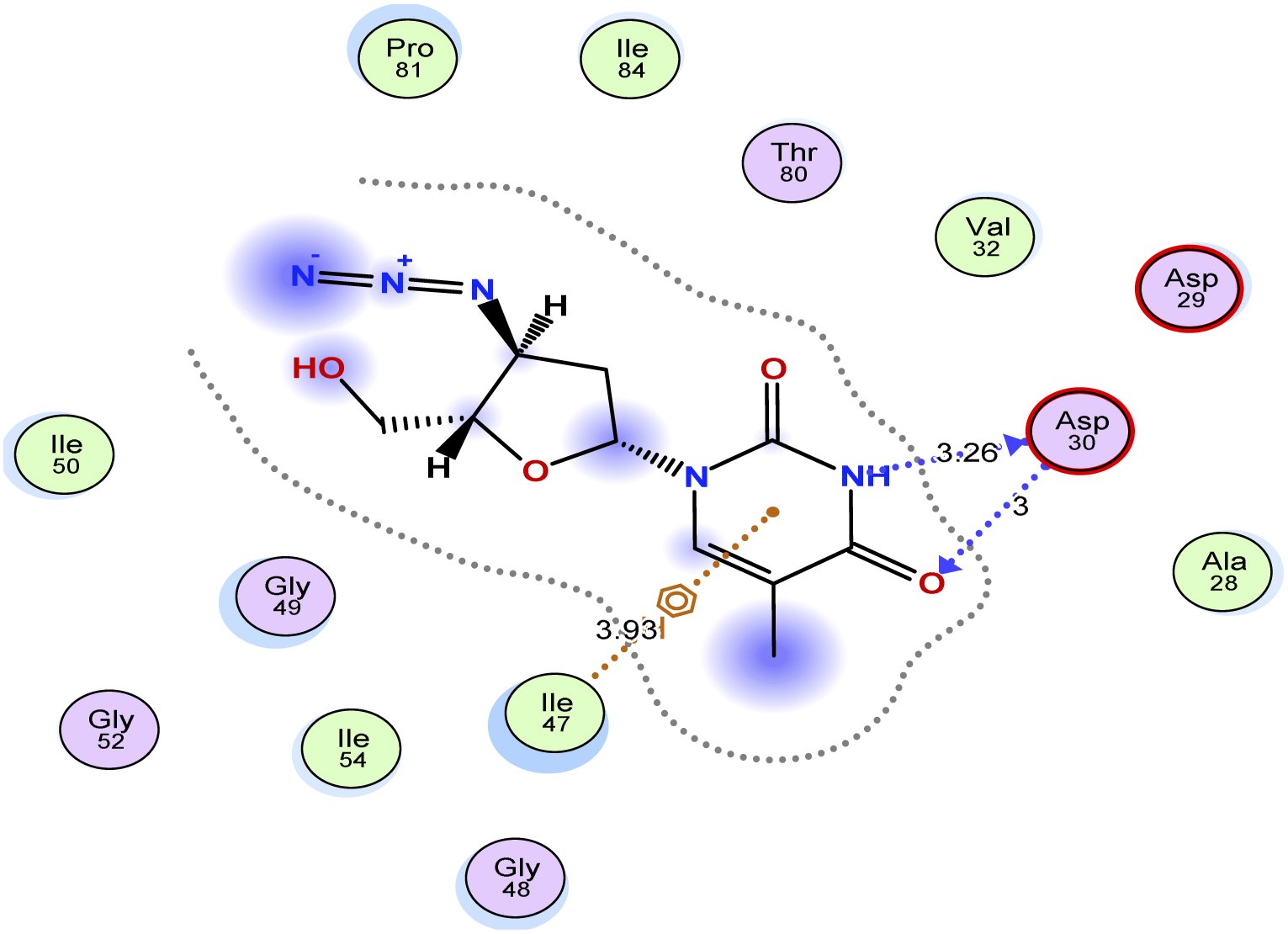	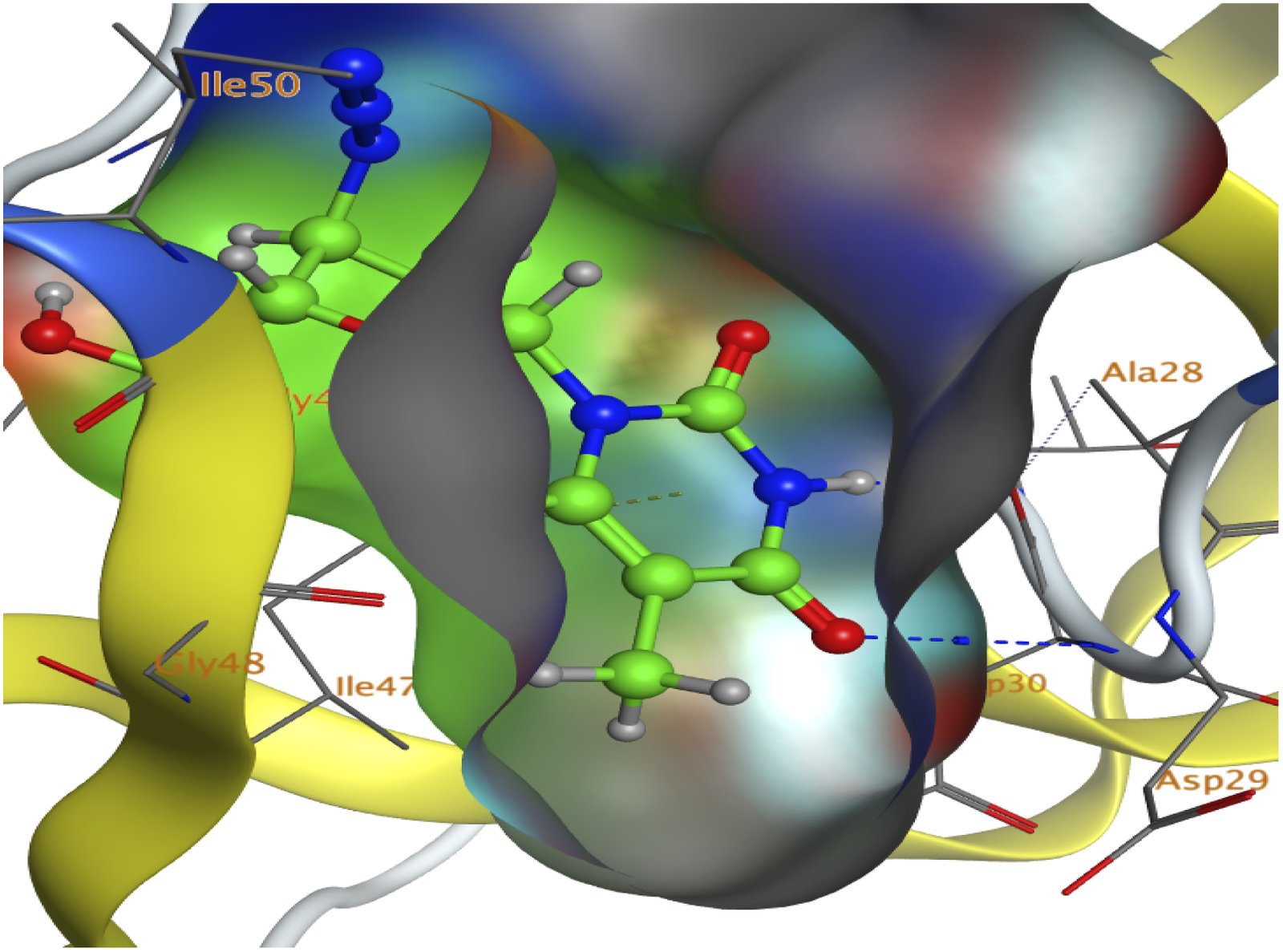

As shown in Table 1, the tested compounds exhibited stronger binding affinities toward the target enzyme than the reference ligand, with acceptable RMSD values of less than 2 Å. The docking scores of the investigated ligands ranged from −6.375 to −6.916 kcal mol^−1^, compared with that of zidovudine (−5.796 kcal mol^−1^). The highest binding interactions were observed for compounds 7b, 7e, 7f, 11b, 11e, and 11f. Compounds 7a and 11b exhibited an H-bond interaction between the S atom of the thiazole moiety and the residue THR 80. Compound 7b showed a hydrogen-bond interaction between the C5 of the thiazole moiety and the side-chain residue ASP30. On the contrary, when comparing the docking poses of 7e and 11d with that of the co-crystallized ligand, it is evident that both molecules occupy similar positions and establish two H-bond acceptor interactions between the O atom and the vital backbone residues of ASP29 and ASP30. The predicted binding modes of compound 7f at the active site of the target enzyme suggested a hydrogen-bond acceptor interaction between the coumarin moiety and ASP29. Similarly, 11a ligand was connected to the protein through two hydrogen bonds involving the protein residues ASP25 and ASP30.

The results of the docking analyses showed high docking scores and strong relevant binding modes for the tested compounds with the target enzyme, in agreement with their *in vitro* enzyme inhibitory activity. The tested compounds were able to maintain strong binding with the target receptors and a similar binding mode for the HIV-1 RT enzyme, albeit at slightly different orientations.

The docking results provide qualitative insights into the possible binding modes and ligand–enzyme interactions; however, docking scores alone should not be considered direct predictors of experimental HIV-1 RT inhibitory activity.

#### 2.2.1. Assessment of physicochemical characteristics

The physicochemical features are critical in determining whether a molecule becomes a therapeutic candidate and how well it performs during the significant phases of drug development.^45^ A compound is considered to exhibit drug-likeness molecule (DLM) properties when it satisfies the requirements outlined in Lipinski's “Rule of Five” (Ro5). The establishment of physicochemical filters as protocols to identify compounds with good drug-likeness, like Lipinski's “Rule of 5”, is a technique that has been frequently used.^46^ To assess the physicochemical parameters and DLM properties of the investigated compounds, the SwissADME tool was used,^47^ and the results are summarized in Table 3. A molecule is considered to meet the Ro5 requirements if its molecular weight is less than 500 and its log *P*_0_/*w* (octanol–water partition coefficient) is less than 5, and the hydrogen-bond donors and acceptors are less than 5 and 10, respectively.^46^ According to the Lipinski rule, a therapeutic candidate or oral bioactive drug should not have more than one violation.^48^ All the synthesized compounds fell within the specified ranges according to the predicted ADME data obtained from the computations.^49^ The tested compounds are predicted to obey Lipinski's Rule of Five and to exhibit intestinal absorption properties in humans.

**Table 3:** Physicochemical characteristics of the compounds 7a–f and 11a–f

Cpd	*M*_wt_, g mol^−1^	log *P*_0_/*w*	No. of H-bond donors	No. of H-bond acceptors	TPSA, Å^2^	log *S*	*F* (sp^3^)	Rotatable bonds
7a	386.0	3.050	0	6	69.26	−4.528	0.048	2
7b	400.1	3.619	0	6	69.26	−5.117	0.091	2
7c	420.0	3.892	0	6	69.26	−5.375	0.048	2
7d	463.9	4.097	0	6	69.26	−5.490	0.048	2
7e	431.0	3.066	0	9	112.4	−5.082	0.048	3
7f	454.0	2.829	0	8	99.47	−5.150	0.042	2
11a	414.0	2.403	0	7	86.33	−4.518	0.045	2
11b	444.0	2.533	0	8	95.56	−4.791	0.087	3
11c	448.0	3.267	0	7	86.33	−5.022	0.045	2
11d	432.0	2.558	0	7	86.33	−4.758	0.045	2
11e	459.0	2.379	0	10	129.4	−4.972	0.045	3
11f	464.0	3.683	0	7	86.33	−5.792	0.038	2

As shown in Table 3, the optimal range for each property is as follows: M. Wt. between 150 and 500 g mol^−1^, lipophilicity (log *P*_0_/*w*) under 5, polarity (TPSA) between 20 and 130 Å^2^, solubility (log *S*) higher than −6, and flexibility of no more than 6 rotatable bonds.^50^

Topological polar surface area (TPSA) is widely used as a physicochemical descriptor to estimate polarity and, in turn, to assess properties that influence membrane permeation and transport. In the radar visualization, the shaded pink zone marks the desirable window associated with oral drug-likeness, integrating constraints on molecular size (SIZE), lipophilicity (LIPO), polarity/TPSA, aqueous solubility (INSOLU), saturation (INSATU), and conformational flexibility (FLEX), as depicted in Fig. 1 and S2.

The oral bioavailability graph (Fig. 1) provides an overview of these properties, including potential size, lipophilicity, total polar surface area, degree of saturation, and molecular flexibility.

**Fig. 1:**
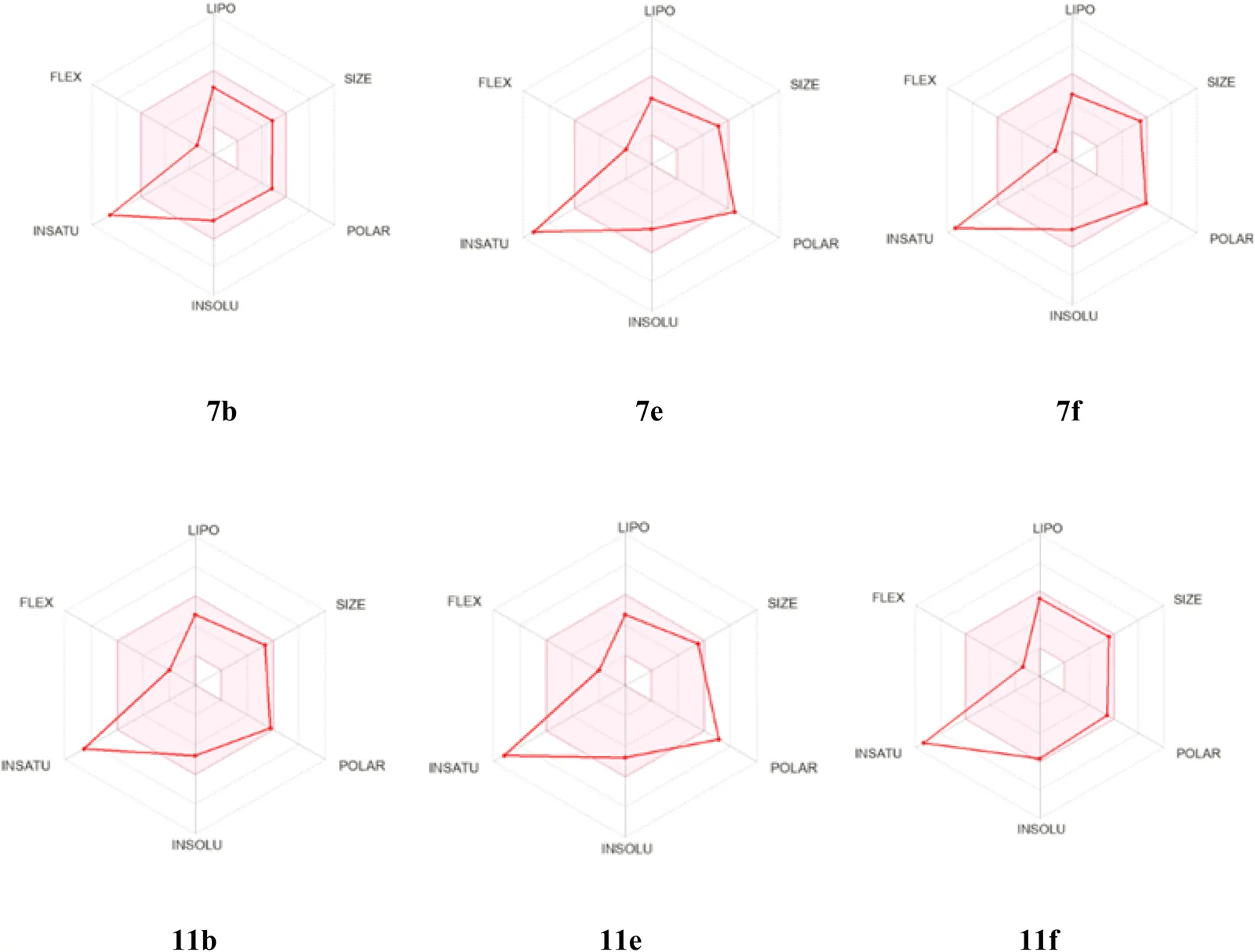
Bioavailability radar of the compounds 7b, 7e, 7f, 11b, 11e and 11f.

#### 2.2.2. *In silico* pharmacokinetic profile ADMET

The ADMET properties of compounds 7a–f and 11a–f were computationally predicted, and the results are summarized in Table 4. The predicted human intestinal absorption values ranged from 89.87 to 100%, while the predicted fraction unbound ranged from 0.283 to 0.37. Identifying CYP isoforms potentially involved in drug metabolism is important because polymorphically expressed isoforms, including CYP2C9 and CYP2C19, may contribute to interindividual pharmacokinetic variability. The CYP superfamily plays a major role in metabolic biotransformation and drug elimination, and inhibition of these enzymes may contribute to pharmacokinetic drug–drug interactions.^51–56^ The computational models classified compounds 7a–d as potential CYP3A4 substrates and predicted them to be inhibitors of CYP1A2, CYP2C19, and CYP2C9. Compounds 7e and 11f were predicted to be CYP3A4 substrates and inhibitors of CYP1A2 and CYP2C9, whereas compounds 7f and 11a–d were predicted to be CYP3A4 substrates and CYP1A2 inhibitors. Compound 11e was predicted to be a CYP3A4 substrate. Predicted total-clearance values ranged from −0.47 to 0.115 log mL min^−1^ kg^−1^. The toxicity model predicted no skin sensitization for the tested compounds. All these values are computational estimates and therefore require experimental confirmation.

**Table 4:** ADMET characteristics of the synthesized compounds 7a–f and 11a–f and zidovudine

Cpd	Absorption	Distribution	Metabolism	Excretion	Toxicity
	HIA (% absorbed)	Fraction unbound			
				Total clearance (log mL min^−1^ kg^−1^)	Skin sensitization
7a	100	0.348	CYP3A4 substrate, CYP1A2, CYP2C19, and CYP2C9 inhibitors	0.115	No
7b	100	0.37		0.057	
7c	99.65	0.363		−0.017	
7d	99.58	0.362		−0.039	
7e	100	0.325	CYP3A4 substrate, CYP1A2, and CYP2C9 inhibitors	0.071	
7f	100	0.344	CYP3A4 substrate and CYP1A2 inhibitor	0.013	
11a	100	0.291		−0.323	
11b	94.51	0.305		−0.291	
11c	100	0.31		−0.455	
11d	100	0.325		−0.47	
11e	89.87	0.283	CYP3A4 substrate	−0.421	
11f	100	0.326	CYP3A4 substrate, CYP1A2, and CYP2C9 inhibitors	−0.364	
Zidovudine	69.94	0.065	—	0.052	

The *in silico* ADMET analysis predicted high intestinal absorption, moderate fraction-unbound values, several potential CYP450 interactions, and no skin sensitization. These computational results suggest potentially favorable pharmacokinetic profiles; however, they do not confirm oral bioavailability and require experimental validation from medicinal researchers.

### 2.3. HIV-1 RT inhibition activity

RT is a key target enzyme for the life cycle of retroviruses like HIV, where it plays a role in viral replication and propagation within host cells. Thus, RT inhibitors as antiviral drugs could block viral replication, offering improved tolerability and resistance profiles. The ability of compounds 7b, 7e, 7f, 11b, 11e, and 11f to inhibit RT was evaluated using zidovudine as the reference drug, and the results are summarized in Table 5. These compounds were selected as representative derivatives to cover different substituent effects, including electron-donating, electron-withdrawing, and extended aromatic/coumarin motifs, as well as favorable docking interactions. Therefore, the SAR discussion is limited to these experimentally tested compounds, and testing the full synthesized library will be required for a more comprehensive SAR evaluation.

**Table 5:** HIV-1 RT inhibitory activities of the compounds 7b, 7e, 7f, 11b, 11e, 11f and zidovudine expressed as IC_50_ values in µg mL^−1^ and µM

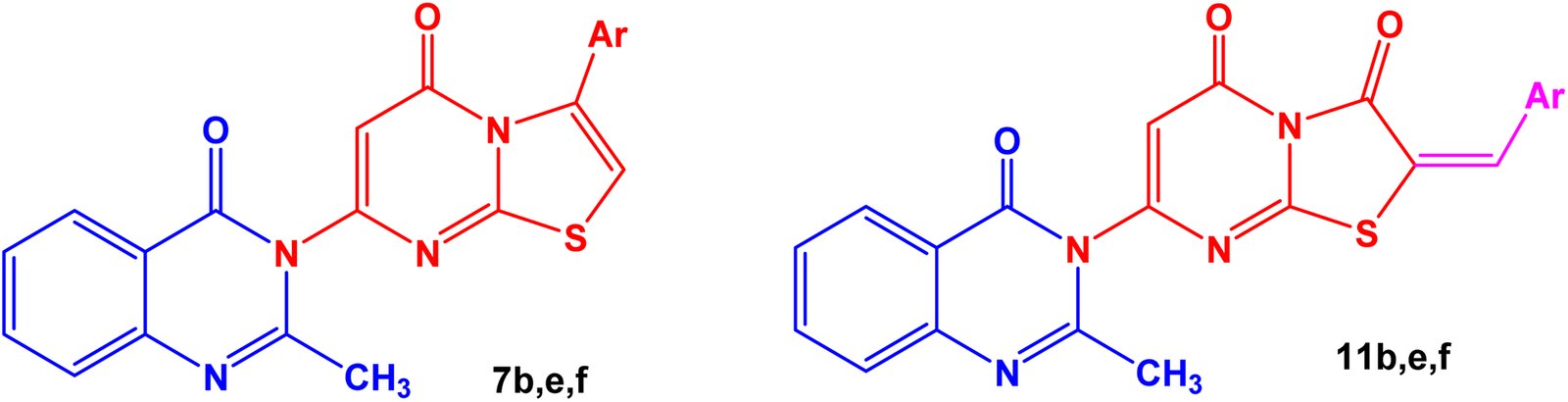			
No.	Ar	IC_50_ (µg mL^−1^)	IC_50_ (µM)
7b	4-CH_3_C_6_H_4_	1.5336 ± 0.066	3.834 ± 0.165
7e	4-NO_2_C_6_H_4_	5.5463 ± 0.238	12.868 ± 0.552
7f	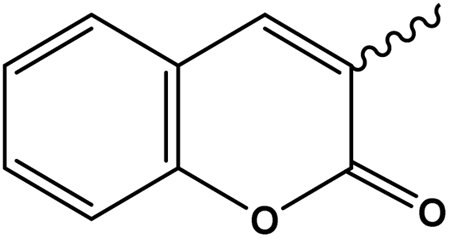	2.0074 ± 0.086	4.422 ± 0.189
11b	4-CH_3_OC_6_H_4_	6.2751 ± 0.269	14.133 ± 0.606
11e	4-NO_2_C_6_H_4_	0.599 ± 0.026	1.305 ± 0.057
11f	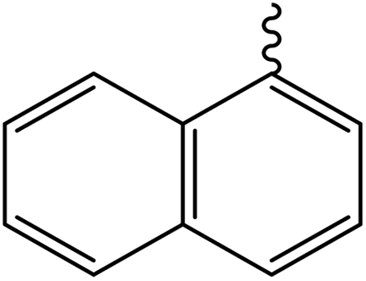	0.9005 ± 0.034	1.941 ± 0.073
Zidovudine	—	0.2024 ± 0.009	0.758 ± 0.034

Although the tested compounds showed favorable docking scores within the HIV-1 RT binding site, the docking results did not fully correlate with the *in vitro* inhibitory activity. For the biologically tested compounds 7b, 7e, 7f, 11b, 11e, and 11f, the correlation between docking score and IC_50_ values was weak (Pearson's *r* ≈ −0.18). Notably, compound 11b showed the best docking score (−6.916 kcal mol^−1^) but relatively weak HIV-1 RT inhibition (IC_50_ = 14.133 µM), whereas 11e displayed the strongest RT inhibition (IC_50_ = 1.305 µM) despite not having the best docking score. Compound 11e was the most active derivative in the synthesized series, with an IC_50_ value of 1.305 µM, although it was less potent than zidovudine (IC_50_ = 0.758 µM).

Therefore, docking was used mainly to support the proposed binding modes and key ligand–enzyme interactions, while the experimental RT inhibition data remain the primary basis for ranking the compounds according to their activity.

Zidovudine was used as the reference compound in this assay to provide a standard comparison with an established nucleoside reverse transcriptase inhibitor. Since zidovudine requires intracellular phosphorylation to its active triphosphate form in cellular systems, its use here should be interpreted only within the context of the present enzyme-based assay. Therefore, the comparison with zidovudine is intended as an assay reference and does not imply cellular antiviral efficacy. Further studies using additional direct RT inhibitor controls, together with cellular antiviral and cytotoxicity assays, will be required to confirm the pharmacological relevance of these compounds.

#### 2.3.1. Structure–activity relationship (SAR)

Because only compounds 7b, 7e, 7f, 11b, 11e, and 11f were experimentally evaluated, the SAR discussion is limited to this tested subset (Table 5). Within the 7-series, the 4-methylphenyl derivative 7b showed the strongest HIV-1 RT inhibition, followed by the coumarin-substituted derivative 7f, whereas the 4-nitrophenyl derivative 7e was less active. These results suggest that, among the tested 7-series compounds, the methyl substituent and extended coumarin system were more favorable than the strongly electron-withdrawing nitro group. Within the tested 11-series, the 4-nitrophenyl derivative 11e was the most active compound, followed by the naphthyl derivative 11f, whereas the 4-methoxyphenyl derivative 11b was less active. Thus, nitro substitution and extended aromaticity appeared to be more favorable than methoxy substitution in this limited subset. These observations represent preliminary SAR trends only; experimental evaluation of the remaining synthesized compounds, including 7c and 7d, is required before broader conclusions can be drawn.

These results identify the tested compounds, particularly 11e and 11f, as preliminary leads for further optimization as HIV-1 RT inhibitors. However, cell-based HIV replication assays, cytotoxicity evaluation in relevant host cells, CC_50_ determination, selectivity index calculation, and resistance-profiling studies are required to confirm their antiviral relevance and therapeutic potential.

## 3. Experimental

### 3.1. Chemistry

#### 3.1.1. Materials and instruments

The melting points of the newly synthesized compounds were determined using an electrothermal Gallenkamp apparatus (IA 9000). FTIR spectra were recorded on a Pye-Unicam SP300 spectrophotometer using KBr discs. ^1^H and ^13^C NMR spectra were acquired on a JEOL 500 MHz spectrometer (500 MHz for ^1^H and 125 MHz for ^13^C). Mass spectra were obtained using a Shimadzu GCMS-Q1000-EX at an ionization voltage of 70 eV. Elemental (CHNS) analyses were performed using an Elementar Vario ELIII analyzer (Germany).

##### 3.1.1.1. General procedure for the reactions of 2-methyl-4*H*-benzo[3,1]oxazin-4-one with heterocyclic amines

7-Amino-5*H*-thiazolo[3,2-*a*]pyrimidine-3,5(2*H*)-dione (2), 6-amino-2-thioxo-2,3-dihydro pyrimidin-4(1*H*)-one (3) or 7-amino-3-aryl-5*H*-thiazolo[3,2-*a*]pyrimidin-5-ones (4a–f) (1 mmol each) was treated with 2-methyl-4*H*-benzo[3,1]oxazin-4-one (1) (0.16 g, 1 mmol) and choline hydroxide (5 mL). The reaction mixture was heated in an oil bath at 230 °C for 6 hours (monitored by TLC). After cooling, ethanol (5 mL) was added and the resulting precipitate was filtered and recrystallized from an EtOH/DMF mixture to give product 5, 6 or 7a–f, respectively.

###### 3.1.1.1.1. 7-(2-Methyl-4-oxoquinazolin-3-yl)-5*H*-thiazolo[3,2-*a*]pyrimidine-3,5(2*H*)-dione (5)

Yellow powder (0.28 g, yield = 85%), mp 217–219 °C; IR (KBr): *υ* = 1680, 1668 (C=O), 1591 (C=N), cm^−1^; ^1^H-NMR (DMSO-*d*_6_): *δ* = 2.47 (s, 3H, CH_3_), 3.87 (s, 2H, CH_2_), 6.82 (s, 1H, pyrimidine-H), 7.05–7.76 (m, 4H, Ar–H) ppm; ^13^C-NMR (DMSO-*d*_6_): *δ* = 17.1 (CH_3_), 34.2 (C-2 thiazole), 85.4 (C-5 pyrimidine), 116.9, 121.0, 121.6, 131.8, 134.3, 143.1, 146.2, 151.9, 156.3 (Ar–Cs), 163.7, 169.5, 174.7 (C=O) ppm; MS (70 eV): *m*/*z* = 326 (M^+^, 34), 184 (100); anal. calcd for C_15_H_10_N_4_O_3_S (326.05): C, 55.21; H, 3.09; N, 17.17%. Found: C, 55.36; H, 3.21; N, 17.29%.

###### 3.1.1.1.2. 2-Methyl-3-(6-oxo-2-thioxo-1,2,3,6-tetrahydropyrimidin-4-yl)quinazolin-4(3*H*)-one (6)

Yellow powder (0.24 g, yield = 85%), mp 233–235 °C; IR (KBr): *υ* = 2260, 2130 (NH), 1682, 1660 (C=O), 1593 (C=N), 1530 (C=S) cm^−1^; ^1^H-NMR (DMSO-*d*_6_): *δ* = 2.47 (s, 3H, CH_3_), 6.81 (s, 1H, pyrimidine-H), 7.45–7.96 (m, 4H, Ar–H), 10.82 (s, 1H, NH), 11.54 (s, 1H, NH) ppm; ^13^C-NMR (DMSO-*d*_6_): *δ* = 17.3 (CH_3_), 85.4 (C-5 pyrimidine), 119.9, 125.6, 127.8 (2C), 133.3, 146.1, 156.2, 158.9 (Ar–Cs), 164.7, 167.5 (C=O), 174.3 (C=S) ppm; MS (70 eV): *m*/*z* = 286 (M^+^, 21), 64 (100); anal. calcd for C_13_H_10_N_4_O_2_S (286.05): C, 54.54; H, 3.52; N, 19.57%. Found: C, 54.38; H, 3.41; N, 19.40%.

###### 3.1.1.1.3. 7-(2-Methyl-4-oxoquinazolin-3-yl)-3-phenyl-5*H*-thiazolo[3,2-*a*]pyrimidin-5-one (7a)

Yellow powder (0.32 g, yield = 83%), mp 206–208 °C; IR (KBr): *υ* = 1690, 1665 (C=O), 1593 (C=N), cm^−1^; ^1^H-NMR (DMSO-*d*_6_): *δ* = 2.47 (s, 3H, CH_3_), 6.36 (s, 1H, pyrimidine-H), 6.95 (s, 1H, thiazole-H), 7.02–8.01 (m, 9H, Ar–H) ppm; ^13^C-NMR (DMSO-*d*_6_): *δ* = 16.9 (CH_3_), 85.5 (C-5 pyrimidine), 112.6 (C-2 thiazole), 116.3, 116.8, 120.4, 120.9, 122.5, 122.9, 123.4, 128.9, 131.0, 131.5, 132.6, 134.4, 141.4, 152.3 (Ar–Cs), 163.8, 168.9 (C=O) ppm; MS (70 eV): *m*/*z* = 386 (M^+^, 18), 90 (100); anal. calcd for C_21_H_14_N_4_O_2_S (386.08): C, 65.27; H, 3.65; N, 14.50%. Found: C, 65.35; H, 3.42; N, 14.39%.

###### 3.1.1.1.4. 7-(2-Methyl-4-oxoquinazolin-3-yl)-3-(*p*-tolyl)-5*H*-thiazolo[3,2-*a*]pyrimidin-5-one (7b)

Yellow powder (0.33 g, yield = 83%), mp 213–215 °C; IR (KBr): *υ* = 1690, 1666 (C=O), 1597 (C=N), cm^−1^; ^1^H-NMR (DMSO-*d*_6_): *δ* = 2.35 (s, 3H, Ar-CH_3_), 2.47 (s, 3H, CH_3_), 6.37 (s, 1H, pyrimidine-H), 6.91 (s, 1H, thiazole-H), 7.14–7.91 (m, 8H, Ar–H) ppm; ^13^C-NMR (DMSO-*d*_6_): *δ* = 17.6 (CH_3_), 22.4 (Ar-CH_3_), 85.4 (C-5 pyrimidine), 110.1 (C-2 thiazole), 120.7, 126.1, 126.7, 127.4 (2C), 128.5 (2C), 129.1 (2C), 131.8, 146.2, 148.6, 151.9, 156.3 (Ar–Cs), 163.8, 169.4 (C=O) ppm; MS (70 eV): *m*/*z* = 400 (M^+^, 47), 90 (100); anal. calcd. for C_22_H_16_N_4_O_2_S (400.10): C, 65.99; H, 4.03; N, 13.99%. Found: C, 66.08; H, 3.92; N, 14.09%.

###### 3.1.1.1.5. 7-(2-Methyl-4-oxoquinazolin-3-yl)-3-(4-chlorophenyl)-5*H*-thiazolo[3,2-*a*]pyrimidin-5-one (7c)

Yellow powder (0.34 g, yield = 82%), mp 241–243 °C; IR (KBr): *υ* = 1691, 1666 (C=O), 1594 (C=N), cm^−1^; ^1^H-NMR (DMSO-*d*_6_): *δ* = 2.47 (s, 3H, CH_3_), 6.33 (s, 1H, pyrimidine-H), 6.95 (s, 1H, thiazole-H), 7.36–8.02 (m, 8H, Ar–H) ppm; ^13^C-NMR (DMSO-*d*_6_): *δ* = 17.3 (CH_3_), 85.1 (C-5 pyrimidine), 113.1 (C-2 thiazole), 120.8, 122.3, 126.1, 126.7, 127.4, 128.5, 129.1, 133.8, 134.8, 142.1, 146.2, 148.6, 151.9, 156.3 (Ar–Cs), 164.1, 168.5 (C=O) ppm; MS (70 eV): *m*/*z* = 420 (M^+^, 23), 198 (100); anal. calcd for C_21_H_13_ClN_4_O_2_S (420.04): C, 59.93; H, 3.11; N, 13.31%. Found: C, 60.05; H, 3.28; N, 13.49%.

###### 3.1.1.1.6. 7-(2-Methyl-4-oxoquinazolin-3-yl)-3-(4-bromophenyl)-5*H*-thiazolo[3,2-*a*]pyrimidin-5-one (7d)

Yellow powder (0.38 g, yield = 82%), mp 230–232 °C; IR (KBr): *υ* = 1691, 1668 (C=O), 1594 (C=N), cm^−1^; ^1^H-NMR (DMSO-*d*_6_): *δ* = 2.47 (s, 3H, CH_3_), 6.33 (s, 1H, pyrimidine-H), 6.69 (s, 1H, thiazole-H), 7.26–7.94 (m, 8H, Ar–H) ppm; ^13^C-NMR (DMSO-*d*_6_): *δ* = 17.3 (CH_3_), 85.2 (C-5 pyrimidine), 110.9 (C-2 thiazole), 120.7, 122.3, 126.1, 126.8, 127.4, 128.5, 131.1, 133.8, 134.7, 142.1, 146.3, 148.6, 151.9, 156.4 (Ar–Cs), 164.1, 168.4 (C=O) ppm; MS (70 eV): *m*/*z* = 463 (M^+^, 32), 154 (100); anal. calcd for C_21_H_13_BrN_4_O_2_S (463.99): C, 54.21; H, 2.82; N, 12.04%. Found: C, 54.08; H, 2.68; N, 11.97%.

###### 3.1.1.1.7. 7-(2-Methyl-4-oxoquinazolin-3-yl)-3-(4-nitrophenyl)-5*H*-thiazolo[3,2-*a*]pyrimidin-5-one (7e)

Yellow powder (0.34 g, yield = 80%), mp 264–266 °C; IR (KBr): *υ* = 1690, 1665 (C=O), 1592 (C=N), cm^−1^; ^1^H-NMR (DMSO-*d*_6_): *δ* = 2.47 (s, 3H, CH_3_), 6.32 (s, 1H, pyrimidine-H), 6.71 (s, 1H, thiazole-H), 7.19–8.31 (m, 8H, Ar–H) ppm; ^13^C-NMR (DMSO-*d*_6_): *δ* = 17.8 (CH_3_), 85.4 (C-5 pyrimidine), 111.1 (C-2 thiazole), 120.8, 123.1, 126.1, 126.8, 127.4, 127.9, 133.5, 140.8, 142.6, 146.2, 148.6, 149.7, 151.9, 156.3 (Ar–Cs), 163.7, 169.5 (C=O) ppm; MS (70 eV): *m*/*z* = 431 (M^+^, 47), 138 (100); anal. calcd for C_21_H_13_N_5_O_4_S (431.07): C, 58.46; H, 3.04; N, 16.23%. Found: C, 58.34; H, 3.12; N, 16.38%.

###### 3.1.1.1.8. 7-(2-Methyl-4-oxoquinazolin-3-yl)-3-(2-oxo-2*H*-chromen-3-yl)-5*H*-thiazolo[3,2-*a*]pyrimidin-5-one (7f)

Yellow powder (0.36 g, yield = 80%), mp 250–252 °C; IR (KBr): *υ* = 1692, 1665 (C=O), 1594 (C=N), cm^−1^; ^1^H-NMR (DMSO-*d*_6_): *δ* = 2.47 (s, 3H, CH_3_), 6.34 (s, 1H, pyrimidine-H), 6.92 (s, 1H, thiazole-H), 7.08–7.93 (m, 9H, Ar–H and coumarin-H) ppm; ^13^C-NMR (DMSO-*d*_6_): *δ* = 17.4 (CH_3_), 85.4 (C-5 pyrimidine), 116.1 (C-2 thiazole), 118.4, 120.8, 125.6, 126.2, 126.8, 127.4, 128.5, 130.4, 133.8, 134.3, 140.2, 142.1, 146.2, 148.6, 151.9, 153.6, 156.3, 158.4 (Ar–Cs), 163.7, 167.4, 169.5 (C=O) ppm; MS (70 eV): *m*/*z* = 454 (M^+^, 30), 123 (100); anal. calcd for C_24_H_14_N_4_O_4_S (454.07): C, 63.43; H, 3.11; N, 12.33%. Found: C, 63.38; H, 3.22; N, 12.44%.

#### 3.2.1. Alternative synthesis of compounds 5 or 7a

2-Methyl-3-(6-oxo-2-thioxo-1,2,3,6-tetrahydropyrimidin-4-yl)quinazolin-4(3*H*)-one (6) (0.286 g, 1 mmol) was mixed with chloroacetic acid (8) (0.094 g, 1 mmol) or phenacyl bromide (9) (0.2 g, 1 mmol) in absolute ethanol (30 mL) containing choline hydroxide (0.24 g, 2 mmol). The reaction mixture was heated under refluxing for 6 hours and the resulting precipitate was filtered and crystallized from an EtOH/DMF mixture to afford an authentic sample of compound 5 or 7a, respectively.

##### 3.2.1.1. Reactions of compound 3 with aldehydes (10a–f)

An equimolar amount of 7-(2-methyl-4-oxoquinazolin-3-yl)-5*H*-thiazolo[3,2-*a*]pyrimidine-3,5(2*H*)-dione (5) (0.33 g, 1 mmol) and the appropriate aldehyde (10a–f) (1 mmol each) was dissolved in 30 mL of absolute ethanol containing choline hydroxide (0.24 g, 2 mmol). The reaction mixture was heated under reflux for 6 hours (monitored by TLC). After completion, water was added to the mixture, and the precipitated solid was collected by filtration, washed with water, and recrystallized from EtOH to give the pure products 11a–f.

###### 3.2.1.1.1. 2-Benzylidine-7-(2-methyl-4-oxoquinazolin-3-yl)-5*H*-thiazolo[3,2-*a*]pyrimidine-3,5(2*H*)-dione (11a)

Yellow powder (0.35 g, yield = 85%), mp 247–249 °C; IR (KBr): *υ* = 1690, 1665 (C=O), 1593 (C=N), cm^−1^; ^1^H-NMR (DMSO-*d*_6_): *δ* = 2.47 (s, 3H, CH_3_), 6.88 (s, 1H, pyrimidine-H), 7.11–7.92 (m, 9H, Ar–H), 8.36 (s, 1H, CH=C) ppm; ^13^C-NMR (DMSO-*d*_6_): *δ* = 17.0 (CH_3_), 85.4 (C-5 pyrimidine), 117.6, 121.4, 122.3, 123.9, 125.7, 128.8, 130.1, 131.8, 132.0, 134.3, 142.3, 145.1, 150.2, 151.9, 159.0 (Ar–Cs), 163.7, 168.5, 174.6 (C=O) ppm; MS (70 eV): *m*/*z* = 414 (M^+^, 35), 115 (100); anal. calcd for C_22_H_14_N_4_O_3_S (414.08): C, 63.76; H, 3.41; N, 13.52%. Found: C, 63.56; H, 3.27; N, 13.38%.

###### 3.2.1.1.2. 2-(4-Methoxybenzylidine)-7-(2-methyl-4-oxoquinazolin-3-yl)-5*H*-thiazolo[3,2-*a*]pyrimidine-3,5(2*H*)-dione (11b)

Yellow powder (0.38 g, yield = 85%), mp 236–238 °C; IR (KBr): *υ* = 1691, 1666 (C=O), 1593 (C=N), cm^−1^; ^1^H-NMR (DMSO-*d*_6_): *δ* = 2.47 (s, 3H, CH_3_), 3.82 (s, 3H, OCH_3_), 6.75 (s, 1H, pyrimidine-H), 7.02–7.91 (m, 8H, Ar–H), 8.45 (s, 1H, CH=C) ppm; ^13^C-NMR (DMSO-*d*_6_): *δ* = 18.4 (CH_3_), 54.2 (OCH_3_), 84.6 (C-5 pyrimidine), 116.8, 120.6, 126.8 (2C), 127.3 (2C), 128.3 (2C), 133.5, 135.4, 143.2, 146.2, 151.8, 156.3, 158.3 (Ar–Cs), 163.7, 168.5, 173.8 (C=O) ppm; MS (70 eV): *m*/*z* = 444 (M^+^, 24), 62 (100); anal. calcd for C_23_H_16_N_4_O_4_S (444.09): C, 62.15; H, 3.63; N, 12.61%. Found: C, 62.26; H, 3.52; N, 12.49%.

###### 3.2.1.1.3. 2-(4-Chlorobenzylidine)-7-(2-methyl-4-oxoquinazolin-3-yl)-5*H*-thiazolo[3,2-*a*]pyrimidine-3,5(2*H*)-dione (11c)

Yellow powder (0.37 g, yield = 83%), mp 263–265 °C; IR (KBr): *υ* = 1690, 1665 (C=O), 1593 (C=N), cm^−1^; ^1^H-NMR (DMSO-*d*_6_): *δ* = 2.47 (s, 3H, CH_3_), 6.81 (s, 1H, pyrimidine-H), 7.20–7.88 (m, 8H, Ar–H), 8.43 (s, 1H, CH=C) ppm; ^13^C-NMR (DMSO-*d*_6_): *δ* = 18.6 (CH_3_), 84.4 (C-5 pyrimidine), 116.8, 120.6, 126.7 (2C), 127.2 (2C), 128.6 (2C), 133.5, 135.4, 143.4, 146.6, 151.9, 156.4, 158.2 (Ar–Cs), 163.7, 168.5, 174.1 (C=O) ppm; MS (70 eV): *m*/*z* = 448 (M^+^, 26), 138 (100); anal. calcd for C_22_H_13_ClN_4_O_3_S (448.04): C, 58.87; H, 2.92; N, 12.48%. Found: C, 58.69; H, 2.81; N, 12.35%.

###### 3.2.1.1.4. 2-(4-Fluorobenzylidine)-7-(2-methyl-4-oxoquinazolin-3-yl)-5*H*-thiazolo[3,2-*a*]pyrimidine-3,5(2*H*)-dione (11d)

Yellow powder (0.36 g, yield = 83%), mp 244–246 °C; IR (KBr): *υ* = 1692, 1665 (C=O), 1598 (C=N), cm^−1^; ^1^H-NMR (DMSO-*d*_6_): *δ* = 2.47 (s, 3H, CH_3_), 6.82 (s, 1H, pyrimidine-H), 7.01–7.96 (m, 8H, Ar–H), 8.41 (s, 1H, CH=C) ppm; ^13^C-NMR (DMSO-*d*_6_): *δ* = 18.4 (CH_3_), 84.4 (C-5 pyrimidine), 116.8, 117.5, 120.6, 126.7 (2C), 127.5, 128.6, 130.4, 131.5, 133.5, 143.4, 146.6, 151.9, 156.4, 160.2 (Ar–Cs), 163.7, 168.5, 174.3 (C=O) ppm; MS (70 eV): *m*/*z* = 433 (M^+^, 23), 76 (100); anal. calcd for C_22_H_13_FN_4_O_3_S (433.07): C, 61.11; H, 3.03; N, 12.96%. Found: C, 61.19; H, 2.97; N, 13.05%.

###### 3.2.1.1.5. 2-(4-Nitrobenzylidine)-7-(2-methyl-4-oxoquinazolin-3-yl)-5*H*-thiazolo[3,2-*a*]pyrimidine-3,5(2*H*)-dione (11e)

Yellow powder (0.37 g, yield = 80%), mp 273–275 °C; IR (KBr): *υ* = 1690, 1668 (C=O), 1594 (C=N), cm^−1^; ^1^H-NMR (DMSO-*d*_6_): *δ* = 2.47 (s, 3H, CH_3_), 6.87 (s, 1H, pyrimidine-H), 7.29–8.13 (m, 8H, Ar–H), 8.36 (s, 1H, CH=C) ppm; ^13^C-NMR (DMSO-*d*_6_): *δ* = 18.4 (CH_3_), 83.9 (C-5 pyrimidine), 116.4, 120.4, 123.7, 126.1, 126.8, 127.3, 128.4, 129.1, 133.5, 136.4, 141.1, 146.9, 151.9, 156.3, 158.3 (Ar–Cs), 163.7, 168.5, 173.7 (C=O) ppm; MS (70 eV): *m*/*z* = 459 (M^+^, 27), 122 (100); anal. calcd for C_22_H_13_N_5_O_5_S (459.06): C, 57.51; H, 2.85; N, 15.24%. Found: C, 57.72; H, 3.03; N, 15.37%.

###### 3.2.1.1.6. 2-(Naphthalen-1-ylmethylene)-7-(2-methyl-4-oxoquinazolin-3-yl)-5*H*-thiazolo[3,2-*a*]pyrimidine-3,5(2*H*)-dione (11f)

Yellow powder (0.37 g, yield = 80%), mp 259–261 °C; IR (KBr): *υ* = 1690, 1666 (C=O), 1598 (C=N), cm^−1^; ^1^H-NMR (DMSO-*d*_6_): *δ* = 2.47 (s, 3H, CH_3_), 6.85 (s, 1H, pyrimidine-H), 7.03–7.73 (m, 11H, Ar–H), 8.39 (s, 1H, CH=C) ppm; ^13^C-NMR (DMSO-*d*_6_): *δ* = 18.1 (CH_3_), 84.5 (C-5 pyrimidine), 116.9, 122.6, 124.5 (2C), 125.3 (2C), 126.8 (2C), 127.3 (2C), 128.4 (2C), 131.2, 133.5, 135.4, 141.5, 143.8, 146.2, 151.9, 156.3, 158.2 (Ar–Cs), 163.5, 168.5, 173.4 (C=O) ppm; MS (70 eV): *m*/*z* = 464 (M^+^, 25), 77 (100); anal. calcd for C_26_H_16_N_4_O_3_S (464.09): C, 67.23; H, 3.47; N, 12.06%. Found: C, 67.11; H, 3.30; N, 11.95%.

### 3.2. Docking study

#### 3.2.1. Ligand preparation

The synthesized structures were first constructed using ChemDraw Professional 16.0 and subsequently subjected to energy minimization using the MMFF94× force field. Geometry optimization was continued until the gradient convergence criterion of 0.1 kcal mol^−1^·Å^−1^ was reached.

#### 3.2.2. Protein preparation

The enzyme's X-ray crystal structure (PDB ID: 6DV4, resolution: 1.14 Å) was downloaded in PDB format from the Protein Data Bank.^57^ The enzyme was prepared for docking study through the following steps:

(1) Water molecules were removed, and hydrogen atoms were added to the structure using the usual geometry. Broken bonds were then reconnected, and potential structural issues were corrected.^58^

(2) The Alpha Site Finder was used to identify the largest binding site. Dummy atoms were generated based on the enzyme structure, and the protein binding pocket was identified.

(3) The identified binding pocket was saved in PDB format and used to dock the most effective drugs and estimate the ligand–enzyme interactions at the active site.

(4) Binding poses were generated with the Triangle Matcher placement approach and ranked using the London dG scoring function, focusing on contacts between the ligand and key active-site residues.

(5) The resulting complexes were then examined using 2D interaction maps and 3D molecular views to identify and confirm ligand–amino acid interactions. Docking parameters, scores, and interaction profiles were reported following the previously described literature workflow.^59,60^

Protein and ligand preparation, together with redocking of the co-crystallized ligand, were performed to validate the docking protocol. The ligand was redocked into its native binding site, and the predicted binding pose was compared with its crystallographic orientation. The co-crystallized ligand (CCL) showed an RMSD of 1.386 Å, which was below the acceptable threshold of 2.0 Å, confirming the reliability of the docking protocol.

#### 3.2.3. *In silico* physicochemical and ADMET

To predict the physiological and ADMET parameters, the SMILE representations of the synthesized compounds were uploaded to the pkCSM web server. Using pkCSM, the pharmacokinetic profiles of the selected synthesized compounds were predicted.^61^ The pkCSM web server, which is publicly available (https://structure.bioc.cam.ac.uk/pkcsm), provides an extensive information platform for quickly assessing pharmacokinetic^62,63^ and toxicity properties.^64^

### 3.3. HIV-1 RT inhibition activity

The antiviral activities of the tested compounds against RT were evaluated using an RT assay kit.^65^

*Negative control*: 0.5% DMSO in reaction buffer (without inhibitor, maximal RT activity).

*Background control*: all components except RT (to measure the non-specific signal).

*Positive control*: the reference drug at several concentrations (for validation).

#### 3.3.1. Assay procedure

##### 3.3.1.1. RT reaction

(i) Set up the reaction mixture (for each well):

• 10 µL template–primer complex

• 10 µL nucleotide mix (with biotin-dUTP)

• 10 µL diluted compound or control

• 10 µL HIV-1 RT enzyme (diluted in 1× reaction buffer)

“Final reaction volume = 40 µL per well.”

(ii) Add the components to the plate as follows (duplicates/triplicates recommended):

• Columns 1–2: background control (RT replaced with buffer)

• Columns 3–4: negative control (0.5% DMSO, no inhibitor)

• Columns 5–20: tested compounds and the reference drug at various concentrations.

(iii) Incubate the plate at 37 °C for 60–90 min (with the plate covered to prevent evaporation).

(iv) Stop the reaction by adding 10 µL of stop solution (if provided), or proceed directly to the detection step.

##### 3.3.1.2. Detection (can be done immediately after RT step)

(a) Transfer 50 µL of each reaction mixture to the corresponding well of the pre-coated 96-well plate.

(b) Incubate for 60 min at 37 °C on a shaker (gentle shaking).

(c) Wash three times with 300 µL per well of 1× wash buffer (soak for 30 s during each wash and tap dry).

(d) Add 100 µL per well of anti-biotin HRP conjugate. Incubate for 60 min at 37 °C.

(e) Wash five times as described in step (c).

(f) Add 100 µL per well of TMB substrate. Incubate for 10–20 min at room temperature in the dark (color develops).

(g) Stop reaction with 100 µL per well of stop solution. Color changes from blue to yellow.

(h) Measure absorbance at 450 nm (reference 620–650 nm) within 30 min.

##### 3.3.1.3. Calculations and data processing

(1) Average the duplicate/triplicate readings.

(2) Subtract the background (wells without RT):

Corrected absorbance = (*A*) sample – (*A*) background.

(3) Normalize activity:

(%) Activity = [corrected (*A*)sample/corrected(*A*) negative control] × 100

(%) Inhibition = 100 − (%) activity

#### 3.2.4. HIV-1 RT inhibition assay full protocol

The anti-HIV-1 RT activity was evaluated using a commercial HIV-1 reverse transcriptase assay kit. The assay was performed in a 96-well format using a template–primer complex, a nucleotide mixture containing biotin-dUTP, HIV-1 RT enzyme, and different concentrations of each tested compound. The compounds were dissolved in DMSO and diluted with reaction buffer, keeping the final DMSO concentration at 0.5%. Zidovudine was used as the positive control, while wells containing 0.5% DMSO without an inhibitor served as the negative control. Background wells contained all assay components except HIV-1 RT.

The reaction mixture contained 10 µL of the template–primer complex, 10 µL of the nucleotide mixture, 10 µL of the tested compound or control, and 10 µL of the diluted HIV-1 RT enzyme. After incubation at 37 °C for 60–90 min, the reaction mixture was transferred to the pre-coated detection plate, followed by incubation with an anti-biotin HRP conjugate and TMB substrate. Absorbance was measured at 450 nm with reference wavelength correction at 620–650 nm.

Each compound was tested in triplicate at concentrations of 0.01, 0.1, 1, 10, and 50 µg mL^−1^. Background absorbance was subtracted, and percentage inhibition was calculated relative to the negative control. IC_50_ values were obtained from dose–response curves by nonlinear regression analysis and are reported as µg mL^−1^ and µM.

## 4. Conclusion

Two novel series of quinazoline-tethered thiazolo[3,2-*a*]pyrimidines, namely 3-aryl-5*H*-thiazolo[3,2-*a*]pyrimidin-5-ones (7a–f) and 2-arylidine-5*H*-thiazolo[3,2-*a*]pyrimidine-3,5(2*H*)-diones (11a–f), were efficiently synthesized using a choline hydroxide-mediated protocol. Selected representative compounds were evaluated for HIV-1 RT inhibition, showing IC_50_ values ranging from 1.305–14.133 µM, compared with zidovudine (0.758 µM). Among the synthesized derivatives, compound 11e was the most active member of the series, with an IC_50_ value of 0.599 µg mL^−1^ (1.305 µM). Molecular docking provided qualitative insights into possible ligand–target interactions, with docking scores ranging from −6.375 to −6.916 kcal mol^−1^ compared with zidovudine (−5.796 kcal mol^−1^). However, the docking scores did not fully correlate with the experimental HIV-1 RT inhibition data and should therefore be interpreted as supportive binding-mode information rather than direct predictors of activity. *In silico* ADMET predictions suggested generally favorable properties, including high intestinal absorption (89.87–100%), a moderate fraction unbound (0.283–0.37), total clearance values ranging from −0.47 to 0.115 log mL min^−1^ kg^−1^, and no predicted skin sensitization. Overall, these findings identify the prepared scaffolds, particularly 11e, as promising preliminary leads for further optimization as HIV-1 RT inhibitors. Nevertheless, additional cell-based antiviral assays, cytotoxicity evaluation, CC_50_ determination, selectivity index calculation, and resistance-profile studies are required to validate their antiviral relevance and therapeutic potential.

## Conflicts of interest

There are no conflicts to declare.

## Supplementary Material

RA-016-D6RA03750J-s001

## Data Availability

The data supporting the findings of this study are available within the article and its supplementary information (SI). Additional data related to this work are available from the corresponding author upon reasonable request. Supplementary information: additional data supporting the molecular docking and *in silico* physicochemical analyses presented in the article. See DOI: https://doi.org/10.1039/d6ra03750j.

## References

[cit1] Bagasra O. (2006). A unified concept of HIV latency. Expert Opin. Biol. Ther..

[cit2] Zhu D.-D., Corona A., Maloccu S., Tramontano E., Wang S., Pannecouque C., De Clercq E., Meng G., Chen F.-E. (2024). Structure-Based design of novel thiazolone[3,2-*a*]pyrimidine derivatives as potent RNase H inhibitors for HIV therapy. Molecules.

[cit3] Zhao K.-X., Zhang Y.-Y., Wang J.-S., Wang S., Corona A., Maloccu S., Tramontano E., Pannecouque C., De Clercq E., Meng G., Wang L., Chen F.-E. (2024). Design, synthesis and biological evaluation of thiazolo[3, 2-*a*]pyrimidine derivatives as novel RNase H inhibitors. Bioorg. Chem..

[cit4] Awadallah F. M. (2008). Synthesis, Pharmacophore Modeling, and Biological Evaluation of Novel 5*H*-Thiazolo[3,2-*a*]pyrimidin-5-one Derivatives as 5-HT_2A_ Receptor Antagonists. Sci. Pharm..

[cit5] Kolb S., Mondesert O., Goddard M.-L., Jullien D., Villoutreix B. O., Ducommun B., Garbay C., Braud E. (2009). Development of Novel Thiazolopyrimidines as CDC25B Phosphatase Inhibitors. Chem. Med. Chem..

[cit6] Khobragade C. N., Bodade R. G., Dawane B. S., Konda S. G., Khandare N. T. (2010). Synthesis and biological activity of pyrazolo[3,4-*d*]thiazolo[3,2-*a*]pyrimidin-4-one derivatives: *in silico* approach. J. Enzyme Inhib. Med. Chem..

[cit7] Zolghadri S., Bahrami A., Khan M. T. H., Munoz-Munoz J., Carcia-Molina F., Garcia-Canovas F., Akbar Saboury A. (2019). A comprehensive review on tyrosinase inhibitors. J. Enzyme Inhib. Med. Chem..

[cit8] Xu Y., Zhou M., Li Y., Li C., Zhang Z., Yu B., Wang R. (2013). Characterization of the Stereochemical Structures of 2H-Thiazolo[3,2-*a*]pyrimidine Compounds and Their Binding Affinities for Anti-apoptotic Bcl-2 Family Proteins. Chem. Med. Chem..

[cit9] Abdel-Megid M., El-mahdy K. M., Elkazak A. M., Zarif S. E., Salem M. E. (2024). Synthetic approaches and biological evaluation of nitrogen bridgehead thiazolo[3,2-*a*]pyrimidines. Results Chem..

[cit10] Alam O., Khan S. A., Siddiqui N., Ahsan W. (2010). Synthesis and pharmacological evaluation of newer thiazolo [3,2-*a*] pyrimidines for anti-inflammatory and antinociceptive activity. Med. Chem. Res..

[cit11] Nirwan S., Chahal V., Kakkar R. (2019). Thiazolidinones: Synthesis, Reactivity, and Their Biological Applications. J. Heterocycl. Chem..

[cit12] Kadlecsik C., Bognar G., Kenari F., Pinter Z., Ribeiro J. C. O., Envall M. G., Carvalho-Silva V. H., Napolitano H. B., Perjesi P. (2025). (*E*)-2-Benzylidenecyclanones: Part XXI—Reaction of Cyclic Chalcone Analogs with Cellular Thiols: Comparison of Reactivity of (*E*)-2-Arylidene-1-Indanone with -1-Tetralone and -1-Benzosuberone Analogs in Thia-Michael Reactions. Int. J. Mol. Sci..

[cit13] Sun J., Wang Y., Zhan Y. (2025). Phenothiazine-functionalized cyclic chalcone derivative: aggregation-induced emission enhancement, high-contrast mechanochromic luminescence beyond 100nm, and its applications in latent fingerprint extraction, anti-counterfeiting and information encryption. Appl. Mater. Today.

[cit14] Feng Y.-C., Luan J.-Y., Jiang N., Wei W.-T., Geng H., Wang D.-B., Wang K.-B., Xue X.-L., Fang Y., Hu Z.-Q. (2025). Cyclic chalcone dye: Solvatochromism, polarity sensitivity and theoretical calculations. J. Mol. Struct..

[cit15] Levin-Elad M., Liptz Y., Bar-Or K. L., Almog J. (2017). 1,2-Indanedione - A winning ticket for developing fingermarks: a validation study. Forensic Sci. Int..

[cit16] Wegermann C. A., Monzani E., Casella L., Ribeiro M. A., Bruzeguini C. E. T., Vilcachagua J. D., Costa L. A. S., Ferreira A. M. C. (2022). Unveiling geometrical isomers and tautomers of isatin-hydrazones by NMR spectroscopy. J. Mol. Struct..

[cit17] Du T., Gao R., Deng Y., Wang C., Zhou Q., Geng Y. (2020). Indandione-Terminated Quinoids: Facile Synthesis by Alkoxide-Mediated Rearrangement Reaction and Semiconducting Properties. Angew. Chem..

[cit18] Jakusová K., Donovalová J., Cigáň M., Gáplovský M., Garaj V., Gáplovský A. (2014). Isatinphenylsemicarbazones as efficient colorimetric sensors for fluoride and acetate anions – Anions induce tautomerism. Spectrochim. Acta, Part A.

[cit19] Rozmer Z., Perjesi P. (2020). (*E*)-2-Benzylidenecyclanones: Part XVI. Study on the interaction of some (*E*)-2-benzylidenebenzosuberone derivatives with serum albumin by UV-vis method, inhibitory effect on topoisomerase. J. Pharm. Biopharm. Res..

[cit20] Gadilohar B. L., Shankarling G. S. (2017). Choline based ionic liquids and their applications in organic transformation. J. Mol. Liq..

[cit21] Niedermeyer H., Hallett J. P., Villar-Garcia I. J., Hunt P. A., Welton T. (2012). Mixtures of ionic liquids. Chem. Soc. Rev..

[cit22] Pereira M. M. A. (2012). Immobilized Ionic Liquids in Organic Chemistry. Curr. Org. Chem..

[cit23] Hu H., Qiu F., Ying A., Yang J., Meng H. (2014). An Environmentally Benign Protocol for Aqueous Synthesis of Tetrahydrobenzo[b]Pyrans Catalyzed by Cost-Effective Ionic Liquid. Int. J. Mol. Sci..

[cit24] Krishnammagari S. K., Jeong Y. T. (2018). An efficient and green synthesis of novel highly functionalized nitrogen-fused pyrido[2',3':3,4]pyrazolo[1,5-a]pyrimidine derivatives using recyclable choline hydroxide. Res. Chem. Intermed..

[cit25] Al-Saleem M. S. M., Al-Humaidi J. Y., Elhenawy A. A., Riyadh S. M., Zaki M. E. A., Jefri O. A., Gomha S. M. (2025). Choline hydroxide mediated eco-friendly synthesis of 5-arylidene thiazol-4(5*H*)-one clubbed coumarin: antimicrobial evaluation and in silico studies. Sci. Nat..

[cit26] El-Naggar M., Gomha S. M., Abd El-Ghany N. A., Abolibda T. Z., Alruwaili A. H., Zaki M. E. A., Ebaid M. S., Mohamed N. A. (2025). Green synthesis and molecular docking studies of bisthiazole and bisthiadiazole derivatives using *p*-aminobenzoic acid chitosan/CuONP composites. Green Chem. Lett. Rev..

[cit27] Elmachkouri Y. A., Irrou E., Ouachtak H., Gomha S. M., Oubella A., Alotaibi S. H., Taha M. L. (2025). *In silico* studies of pyrazolopyranopyrimidine as a potential anticancer inhibitor: Synthesis, network pharmacology, ADMET prediction, molecular docking, and dynamics simulations. J. Mol. Struct..

[cit28] Al-Saleem M. S. M., Ahmed M. S. M., Riyadh S. M., Alruwaili A. H., Zaki M. E. A., Gomha S. M. (2025). Synthesis, Molecular Characterization, and Antimicrobial Evaluation of Hydrazones Derived from 2-Hydrazinobenzimidazole. Drug Des. Dev. Ther..

[cit29] Abolibda T. Z., Fathalla M., Farag B., Zaki M. E. A., Gomha S. M. (2023). Synthesis and molecular docking of some novel 3-thiazolyl-coumarins as inhibitors of VEGFR-2 kinase. Molecules.

[cit30] Alghamdi A., Abouzied A. S., Alamri A., Anwar S., Ansari M., Khadra I., Zaki Y. H., Gomha S. M. (2023). Synthesis, molecular docking, and dynamic simulation targeting main protease (Mpro) of new thiazole clubbed pyridine scaffolds as potential COVID-19 inhibitors. Drug Des. Dev. Ther..

[cit31] Khan S., Iqbal T., Abass K. S., Abdellattif M. H., Gomha S. M., Zaki M. E. A. (2025). Pyrimidine-Attached Oxazolidinone derivatives: synthesis, kinetic profiling and computational evaluation for Anti-Alzheimer’s therapeutics. J. Mol. Struct..

[cit32] Alharbi R. A. K., Riyadh S. M., Al-Kaff N. S., Said M. A. (2024). Newly synthesized Bis-Hydrazono[1,3,4] thiadiazoles as Anti-Alzheimer’s Agents: Greener past and *in vitro* acetylcholinesterase inhibition assay investigations. J. Saudi Chem. Soc..

[cit33] Gomha S. M., Abdelhamid A. O., Kandil O. M., Kandeel S. M., Abdelrehem N. A. (2018). Synthesis and Molecular Docking of Some Novel Thiazoles and Thiadiazoles Incorporating Pyranochromene Moiety as Potent Anticancer Agents. Mini-Rev. Med. Chem..

[cit34] Gomha S. M., Abdelrazek F. M., Abdelrahman A. H., Metz P. (2018). Synthesis of Some New Pyridine-Based Heterocyclic Compounds with Anticipated Anti-Tumor Activity. J. Heterocycl. Chem..

[cit35] Alqarni S., Riyadh S. M., Abouzied A. S., Alqarni A. M., Farag B., Shehata M. R., Zaki M. E. A., Jefri O. A., Gomha S. M. (2026). Benzimidazotriazines as adenosine receptor-targeting anticancer agents: Synthesis, cytotoxicity, DFT, and docking studies. Arab. J. Chem..

[cit36] Noreen F., Zaki M. E. A., Sadeghian N., Tokali F. S., Taslimi P., Alharthy R. D., Şenol H., Zhao X., Çakır F., Gomha S. M., Shafiq Z. (2026). Rational design of phenyl 2,4,5-trichlorobenzenesulfonate based thiosemicarbazones as α-glucosidase and α-amylase inhibitors: Integrating enzymatic evaluation and molecular modeling. RSC Adv..

[cit37] El-Fagal S. F., El-Helw E. A. E., El-Bordany E. A., Ghareeb E. A. (2025). Antioxidant activity, molecular docking, and modeling pharmacokinetics study of some benzo[*f*]quinoline candidates. Sci. Rep..

[cit38] Kim G. A., Ovchinnikova I. G., Nosova E. V., Rusinov G. L., Charushin V. N. (2018). (*E*)-2-(hydroxystyryl)-3-phenylquinazolin-4(3*H*)-ones: synthesis, photochemical and luminescent properties. Arkivoc.

[cit39] Noolvi M. N., Patel H. M. (2013). Synthesis, method optimization, anticancer activity of 2,3,7-trisubstituted Quinazoline derivatives and targeting EGFR-tyrosine kinase by rational approach. Arab. J. Chem..

[cit40] Gaafar A. M., Aly A. S., Abu-Zied K. M., Abdel-Rahman A. E., Helmy M. M. (2016). Chemical Synthesis of Some Novel 6-Aminouracil-2-Thiones and Their Glycoside Analogues. Egypt. J. Chem..

[cit41] Hurst D. T., Beaumont C., Jones D. T. E., Kingsley D. A., Partridge J. D., Rutherford T. J. (1988). The Chemistry of Pyrimidinethiols II. The Preparation and Reactions of Some 2-Arenecarbonylmethylthiopyrimidines. Aust. J. Chem..

[cit42] Cai D., Zhang Z.-H., Chen Y., Yan X.-J., Zhang S.-T., Zou L.-J., Meng L.-H., Li F., Fu B.-J. (2106). Synthesis of some new thiazolo[3,2-a]pyrimidine derivatives and screening of their in vitro antibacterial and antitubercular activities. Med. Chem. Res..

[cit43] Hamama W. S., Berghot M. A., Baz E. A., Gouda M. A. (2011). Synthesis and Antioxidant Evaluation of Some New 3-Substituted Coumarins. Arch. Pharm..

[cit44] Tarasova O., Poroikov V., Veselovsky A. (2018). Molecular Docking Studies of HIV-1 Resistance to Reverse Transcriptase Inhibitors: Mini-Review. Molecules.

[cit45] Gleeson M. P., Hersey A., Montanari D., Overington J. (2011). Probing the links between in vitro potency, ADMET and physicochemical parameters. Nat. Rev. Drug Discovery.

[cit46] Lipinski C. A., Lombardo F., Dominy B. W., Feeney P. J. (2001). Experimental and computational approaches to estimate solubility and permeability in drug discovery and development settings. Adv. Drug Deliv. Rev..

[cit47] Daina A., Michielin O., Zoete V. (2017). SwissADME: a free web tool to evaluate pharmacokinetics, drug-likeness and medicinal chemistry friendliness of small molecules. Sci. Rep..

[cit48] Lipinski C. A. (2004). Lead-and drug-like compounds: the rule-of-five revolution. Drug Discov. Today Technol..

[cit49] Miteva M. A., Violas S., Montes M., Gomez D., Tuffery P., Villoutreix B. O. (2006). FAF-Drugs: free ADME/tox filtering of compound collections. Nucleic Acids Res..

[cit50] Tripathi P., Ghosh S., Talapatra S. N. (2019). Bioavailability prediction of phytochemicals present in *Calotropis procera* (Aiton) R. Br. by using Swiss-ADME tool. World Sci. News.

[cit51] Di L. (2014). The role of drug metabolizing enzymes in clearance. Expert Opin. Drug Metab. Toxicol..

[cit52] Hollenberg P. F. (2002). Characteristics and common properties of inhibitors, inducers, and activators of CYP enzymes. Drug Metab. Rev..

[cit53] Huang S.-M. (2008). *et al.*, New era in drug interaction evaluation: US Food and Drug Administration update on CYP enzymes, transporters, and the guidance process. J. Clin. Pharmacol..

[cit54] Kirchmair J. (2015). *et al.*, Predicting drug metabolism: experiment and/or computation?. Nat. Rev. Drug Discov..

[cit55] Veith H. (2009). *et al.*, Comprehensive characterization of cytochrome P450 isozyme selectivity across chemical libraries. Nat. Biotechnol..

[cit56] Guengerich F. P. (2008). Cytochrome p450 and chemical toxicology. Chem. Res. Toxicol..

[cit57] Mahalingam B., Louis J. M., Hung J., Harrison R. W., Weber I. T. (2001). Structural implications of drug-resistant mutants of HIV-1 protease: high-resolution crystal structures of the mutant protease/substrate analogue complexes. Proteins.

[cit58] Labute P. (2009). Protonate3D: assignment of ionization states and hydrogen coordinates to macromolecular structures. Proteins.

[cit59] Gomha S. M., Al-Hussain S. A., Farag B., Abolibda T. Z., Nayl A. A., Saeed A., Sayed A. R., Zaki M. E. A. (2025). Synthesis and Biological Evaluation of Thiazolyl-Pyrimidine Hybrids as Potential Antiproliferative Agents with Molecular Docking Insights. J. Biochem. Mol. Toxicol..

[cit60] Huwaimel B., Abouzied A. S., Zaki M. E. A., Alamri A., Farag B., Gomha S. M. (2025). Synthesis, Anticancer Evaluation, and Molecular Docking of Novel Thiazolobenzimidazole–Thiazole Hybrids as Potent Colon Cancer Inhibitors. ChemistryOpen.

[cit61] Pires D. E., Blundell T. L., Ascher D. B. (2015). pkCSM: predicting small-molecule pharmacokinetic and toxicity properties using graph-based signatures. J. Med. Chem..

[cit62] Obach R. S., Lombardo F., Waters N. J. (2008). Trend analysis of a database of intravenous pharmacokinetic parameters in humans for 670 drug compounds. Drug Metab. Dispos..

[cit63] Moda T. L., Torres L. G., Carrara A. E., Andricopulo A. D. (2008). PK/DB: database for pharmacokinetic properties and predictive in silico ADME models. Bioinformatics.

[cit64] Cao D., Wang J., Zhou R., Li Y., Yu H., Hou T. (2012). ADMET evaluation in drug discovery. 11. PharmacoKinetics Knowledge Base (PKKB): a comprehensive database of pharmacokinetic and toxic properties for drugs. J. Chem. Inf. Model..

[cit65] Palmer S., Shafer R. W., Merigan T. C. (1999). Hydroxyurea Enhances the Activities of Didanosine, 9-[2-(Phosphonylmethoxy)ethyl]adenine, and 9-[2-(Phosphonylmethoxy)propyl]adenine against Drug-Susceptible and Drug-Resistant Human Immunodeficiency Virus Isolates. Antimicrob. Agents Chemother..

