# Supplementary material for: Quinazoline-tethered thiazolo[3,2-a]pyrimidines and their cyclic chalcone derivatives as HIV-1 reverse transcriptase inhibitors: green synthesis, molecular docking, and in silico studies
Source: RSC Adv. 2026 Sep 28;16(45):58056–72. doi: 10.1039/d6ra03750j (PMC13618335; doi:10.1039/d6ra03750j)
Supplement: Supplementary file 1 [file RA-016-D6RA03750J-s001.pdf]

Supplementary Information (SI) for RSC Advances.
This journal is © The Royal Society of Chemistry

Supporting Information File

Quinazoline-Tethered Thiazolo[3,2-a]pyrimidines and Their Cyclic Chalcone Derivatives as HIV-1 Reverse Transcriptase Inhibitors: Green Synthesis, Molecular Docking, and In Silico Studies

Sobhi M. Gomha[1]*, Sami A. Al-Hussain[2], Yaser A.M Sulaiman[3], Sayed M. Riyadh[4], Basant Farag[5], Mohammad Alhilal[6], Suzan Alhilal [7], Magdi E. A. Zaki[2]*

[1]Department of Chemistry, Faculty of Science, Islamic University of Madinah, Madinah, 42351, Saudi Arabia, smgomha@iu.edu.sa

[2]Department of Chemistry, Faculty of Science, Imam Mohammad Ibn Saud Islamic University (IMSIU), Riyadh 11623, Saudi Arabia; sahussain@imamu.edu.sa (SA), mezaki@imamu.edu.sa (MZ)

[3]Department of Clinical Laboratory Sciences, College of Pharmacy- Tikrit University, Iraq; neimiyaser@tu.edu.iq

[4]Department of Chemistry, Faculty of Science, Cairo University, Cairo 12613, Egypt; riyadh1993@hotmail.com

[5]Department of Chemistry, Faculty of Science, Zagazig University, Zagazig 44519, Egypt. basantfarag@zu.edu.eg

[6]Department of Nursing, Faculty of Health Sciences, Mardin Artuklu University, Mardin, Turkey; mohammadalhilal@artuklu.edu.tr; ORCID: 0000-0002-2832-8409

[7]Department of Medical Services and Techniques, Vocational School of Health Services, Mardin Artuklu University, Mardin, Turkey; suzanalhilal@artuklu.edu.tr; ORCID: 0000-0002-9372-9364

Table S1. 2D and 3D ligand interactions for ligands **7a-f**, **11a-f**, and zidovudine at the 6DV4 binding site of target enzyme

**Cpd.**	**2D Ligand interaction**	**3D Ligand interaction**
**7a**	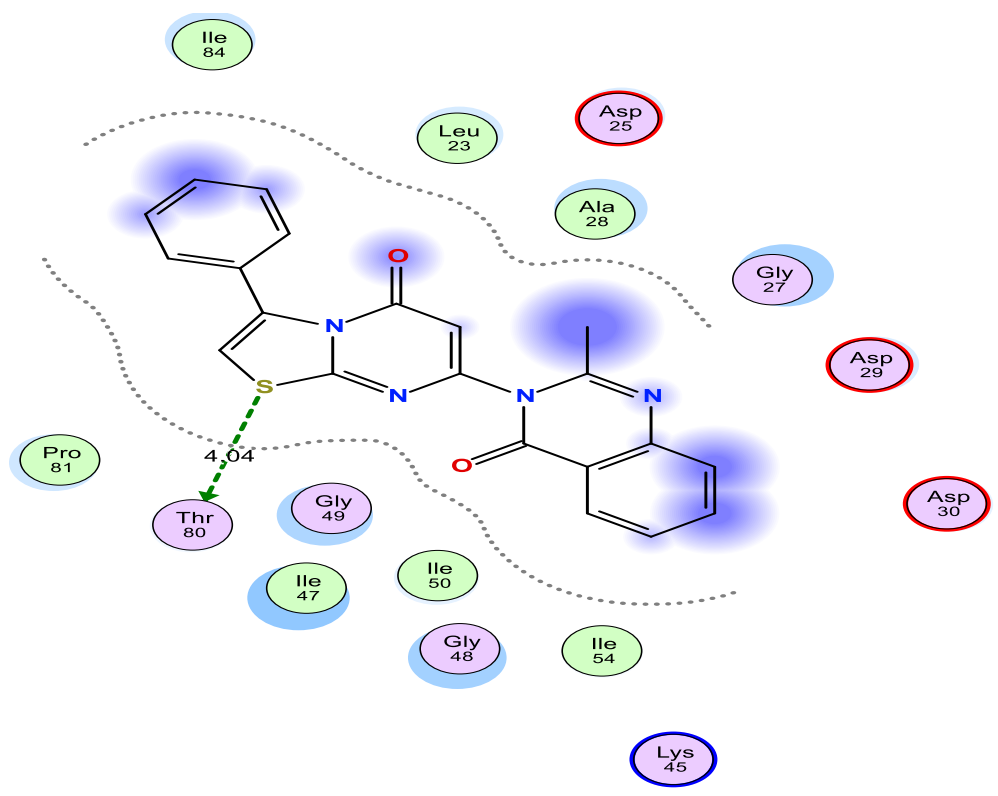	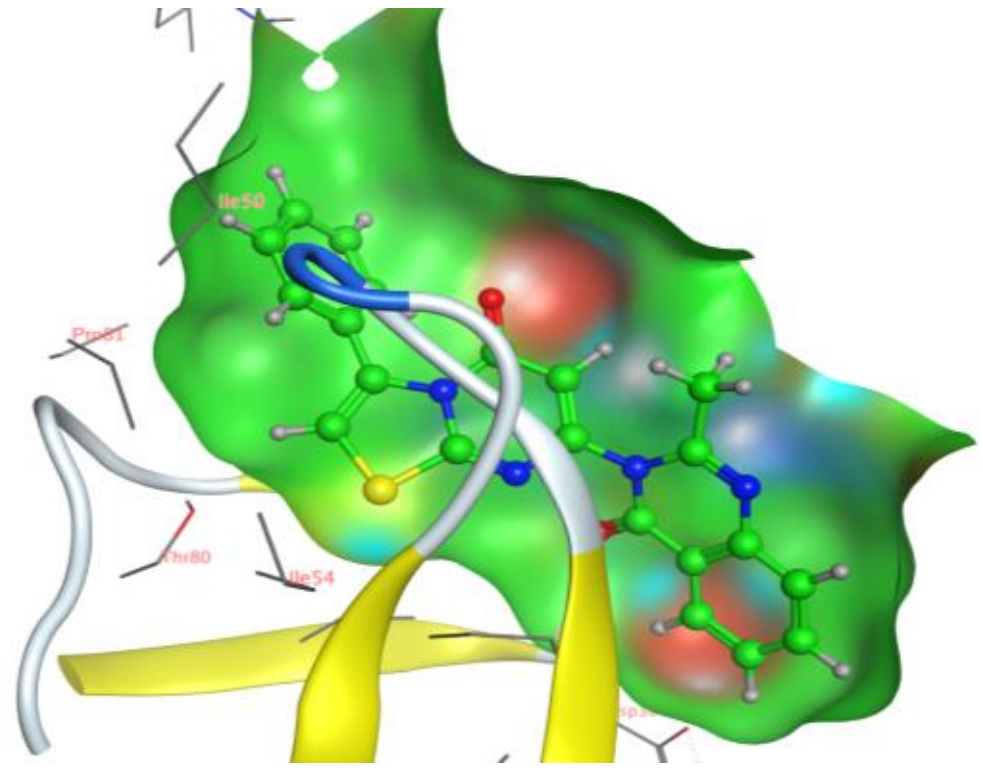
**7b**	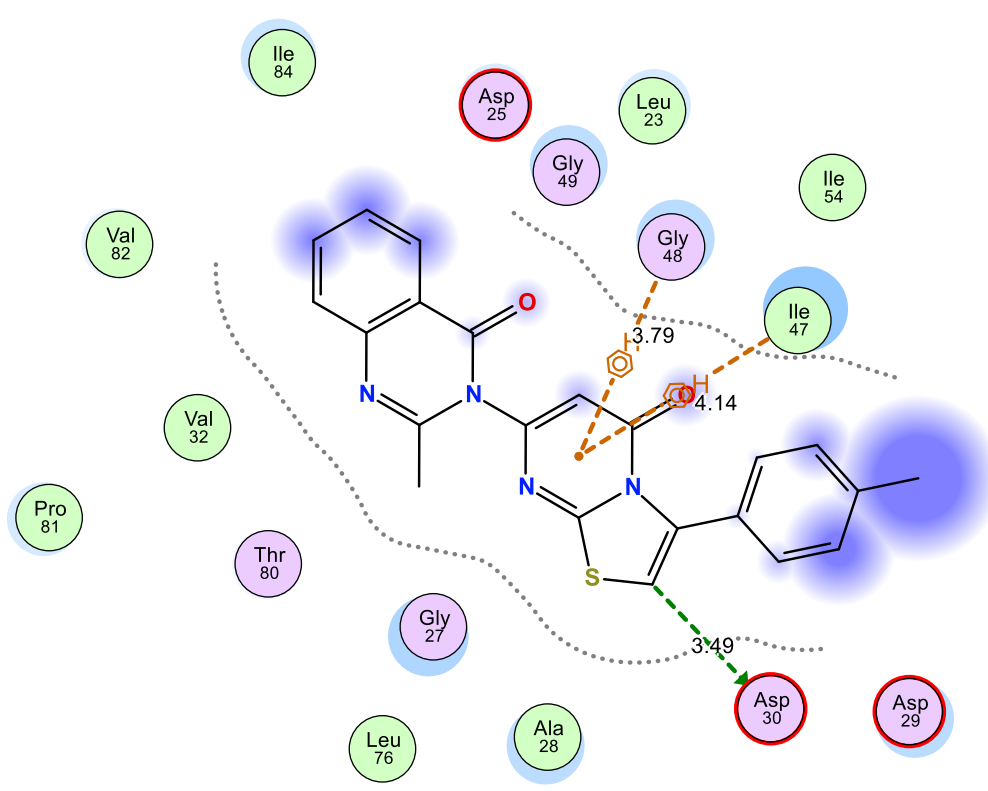	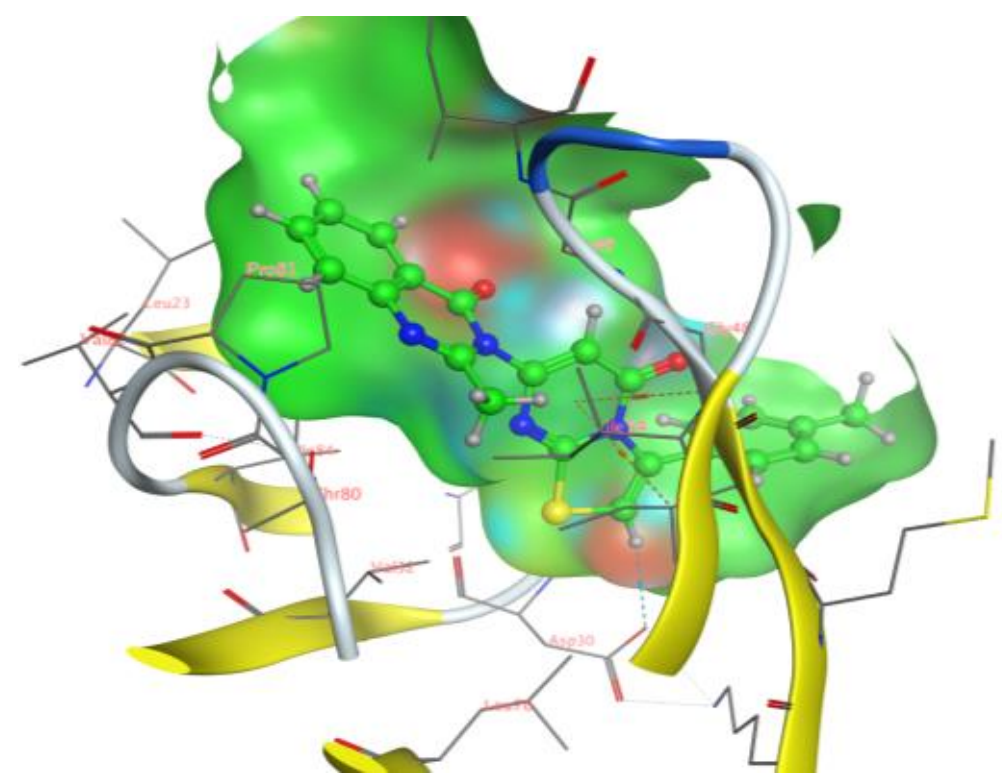
**7c**	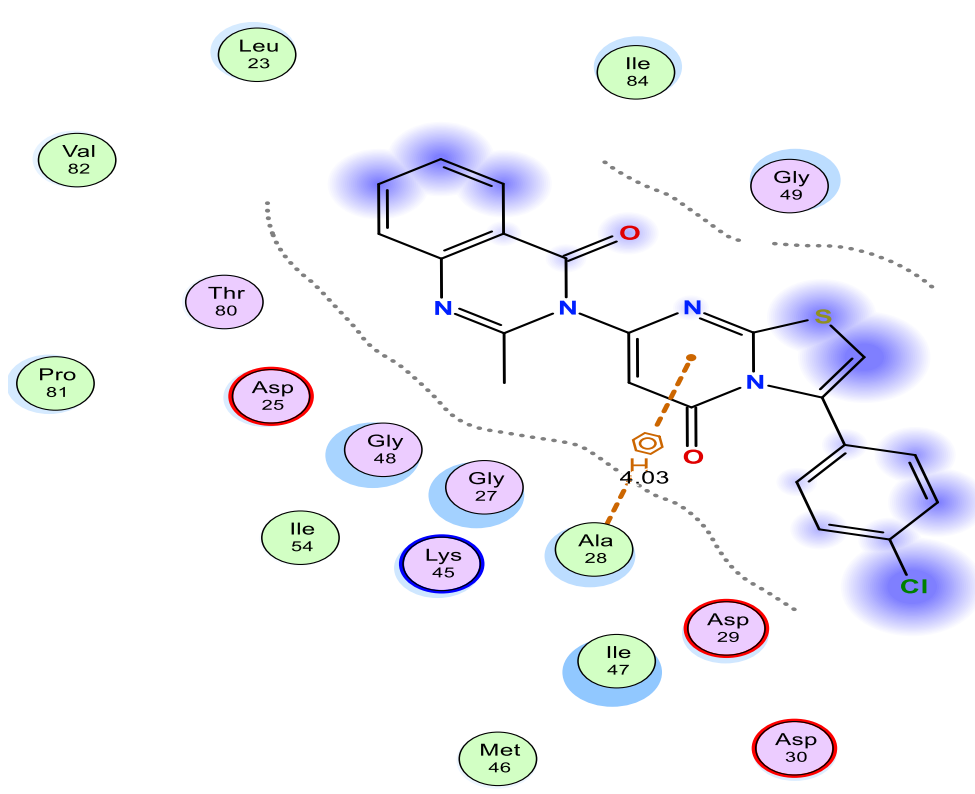	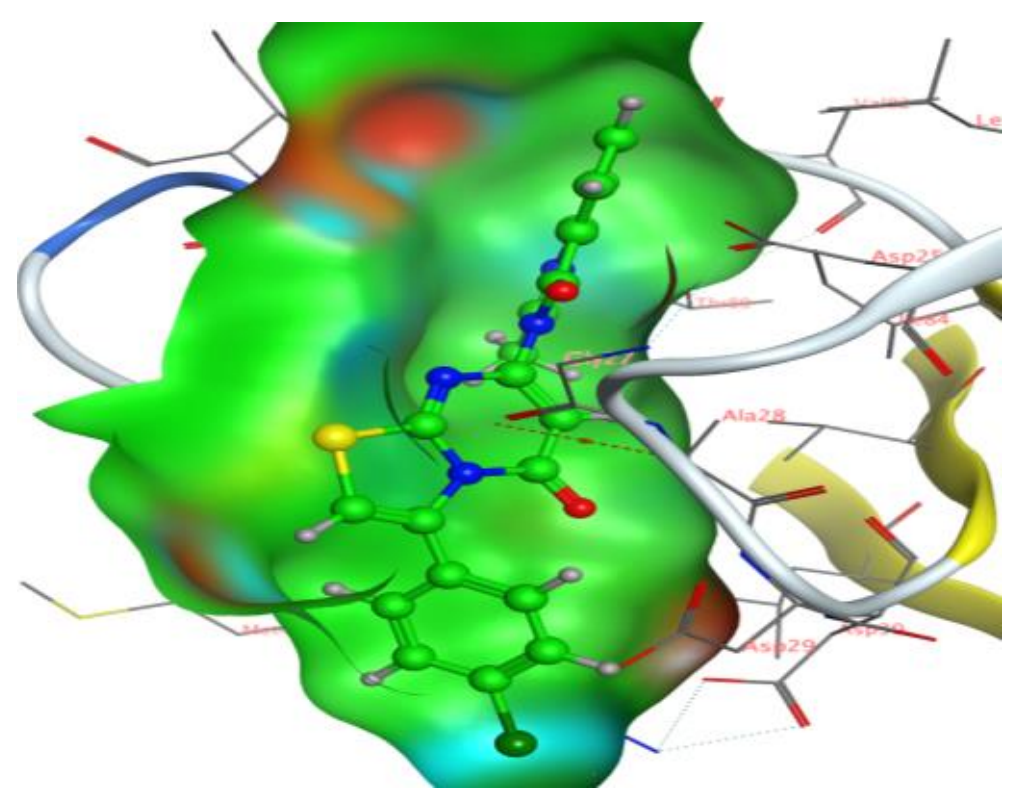

**7d**	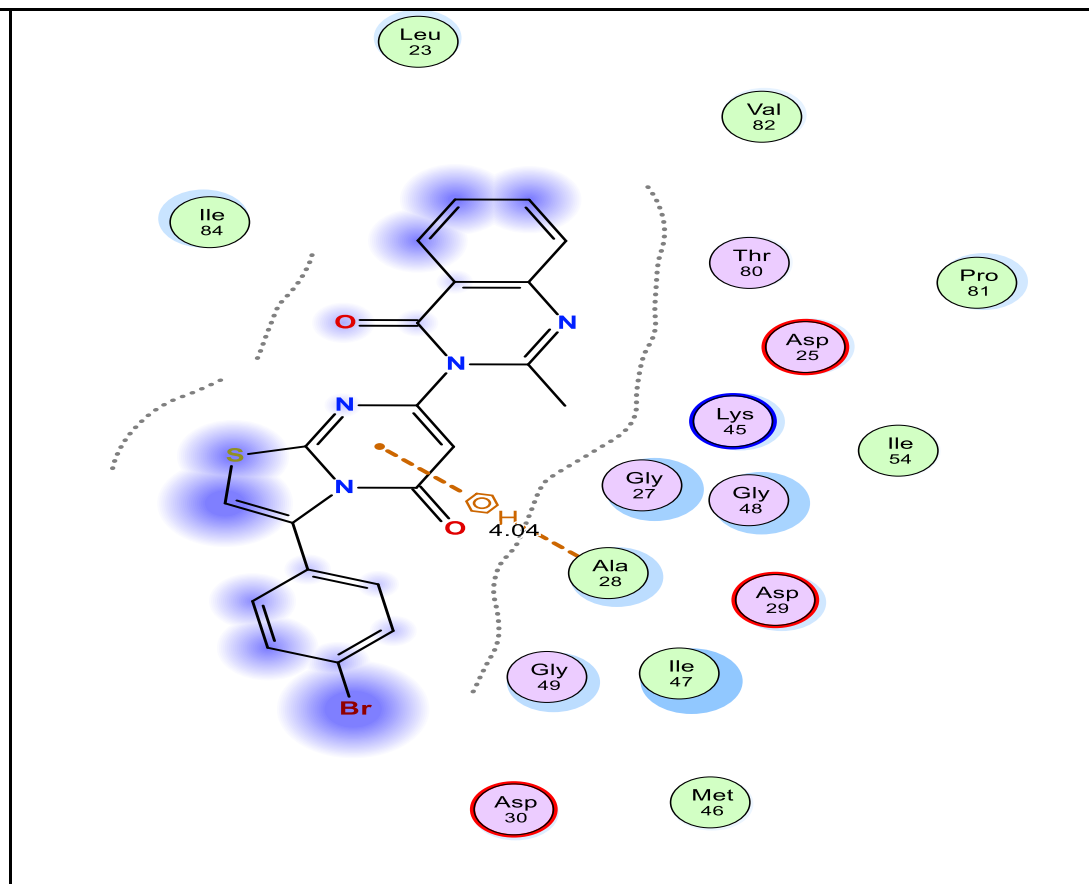 Leu 23 · Val 82 · Ile 84 · Thr 80 · Pro 81 · Asp 25 · Lys 45 · Ile 54 · Gly 27 · Gly 48 · 4.04 · Ala 28 · Asp 29 · Gly 49 · Ile 47 · Asp 30 · Met 46	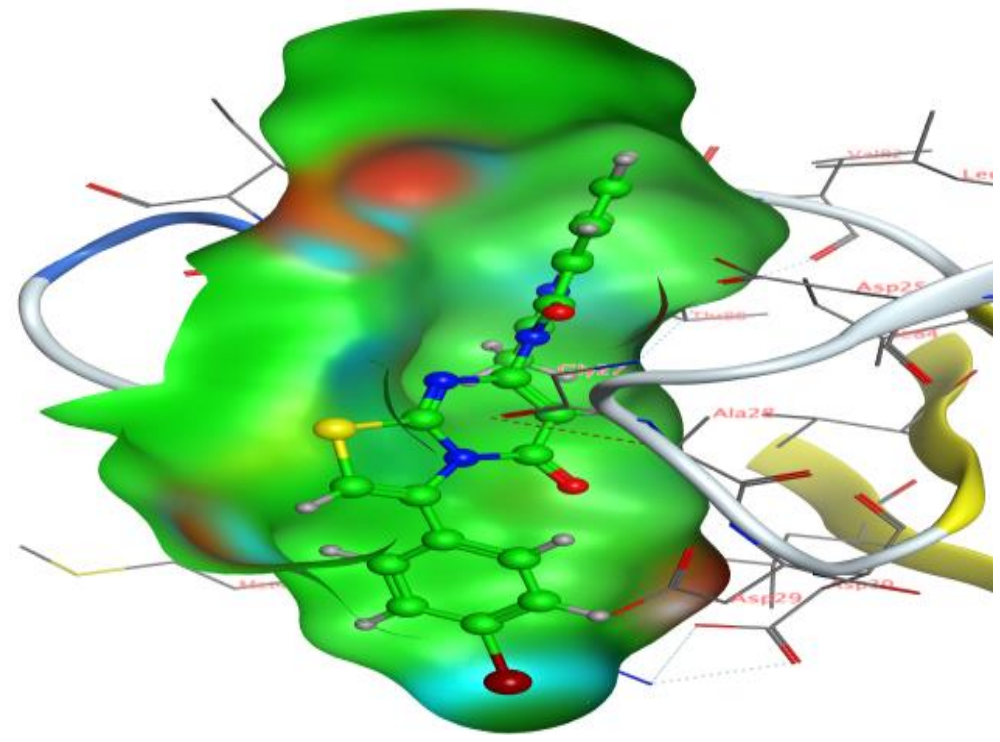
**7e**	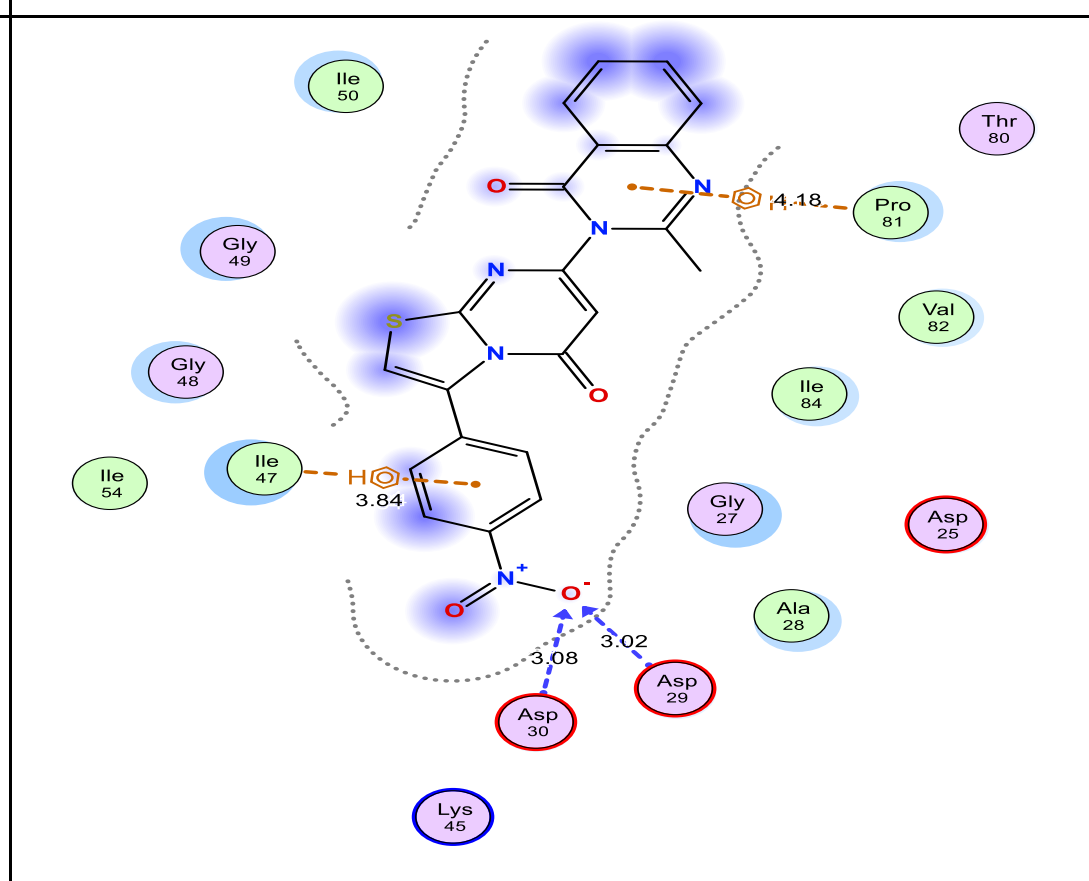 Ile 50 · Thr 80 · 4.18 · Pro 81 · Gly 49 · Val 82 · Gly 48 · Ile 84 · Ile 54 · Ile 47 · 3.84 · Gly 27 · Asp 25 · Ala 28 · 3.08 · 3.02 · Asp 30 · Asp 29 · Lys 45	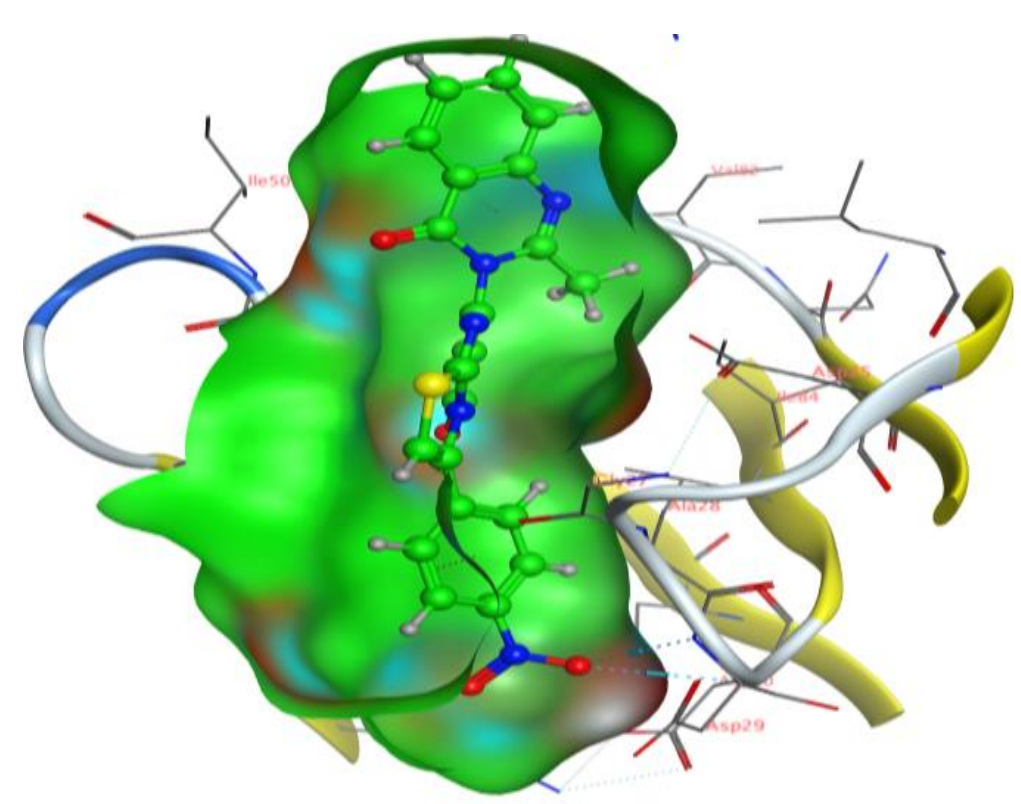
**7f**	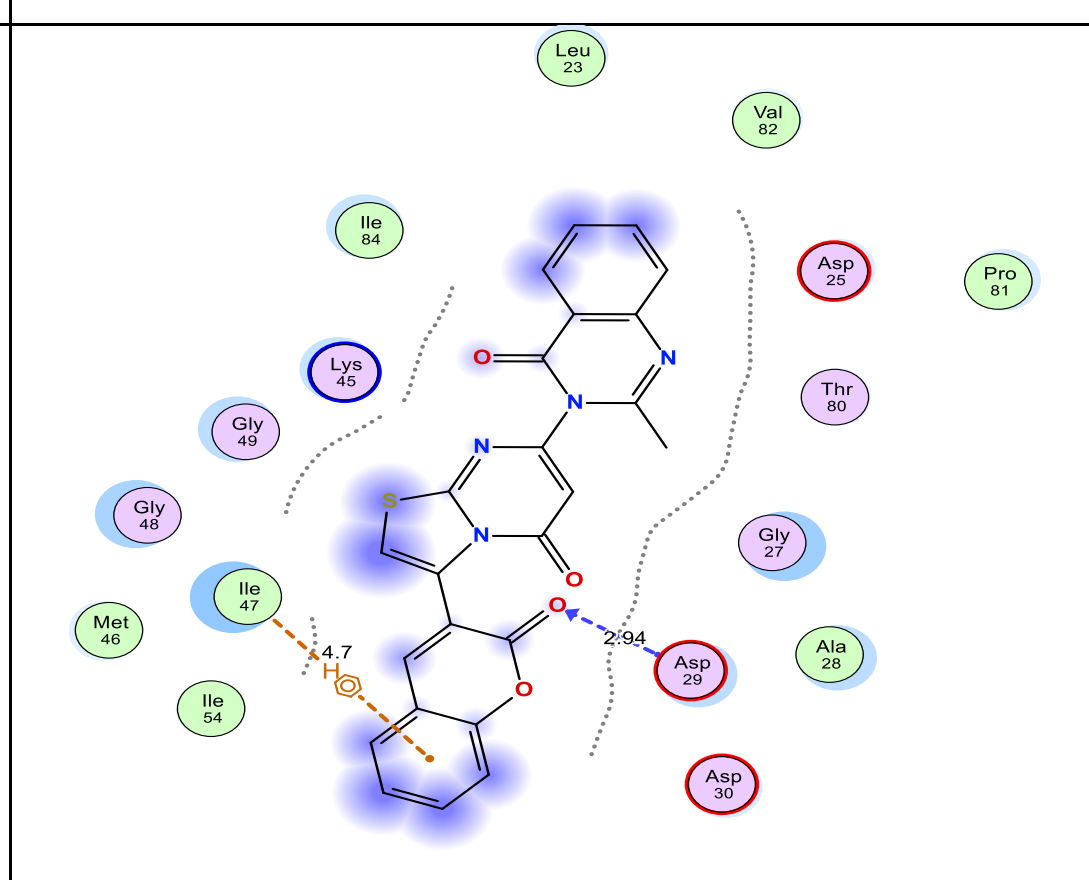 Leu 23 · Val 82 · Ile 84 · Asp 25 · Pro 81 · Lys 45 · Thr 80 · Gly 49 · Gly 48 · Gly 27 · Ile 47 · Met 46 · 4.7 · 2.94 · Asp 29 · Ala 28 · Ile 54 · Asp 30	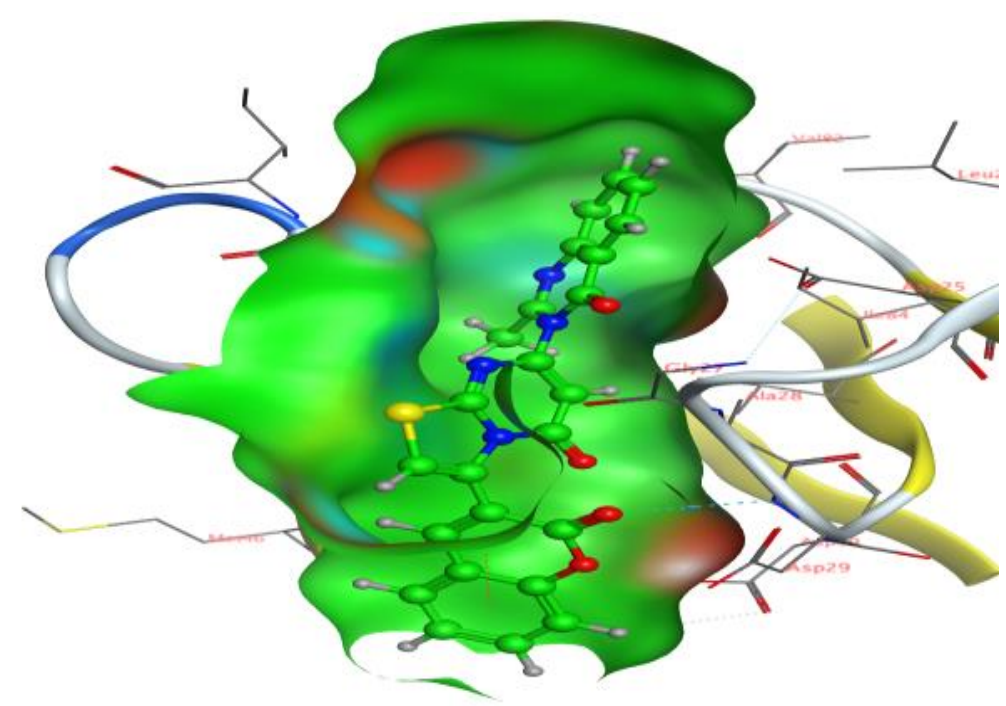
**11a**	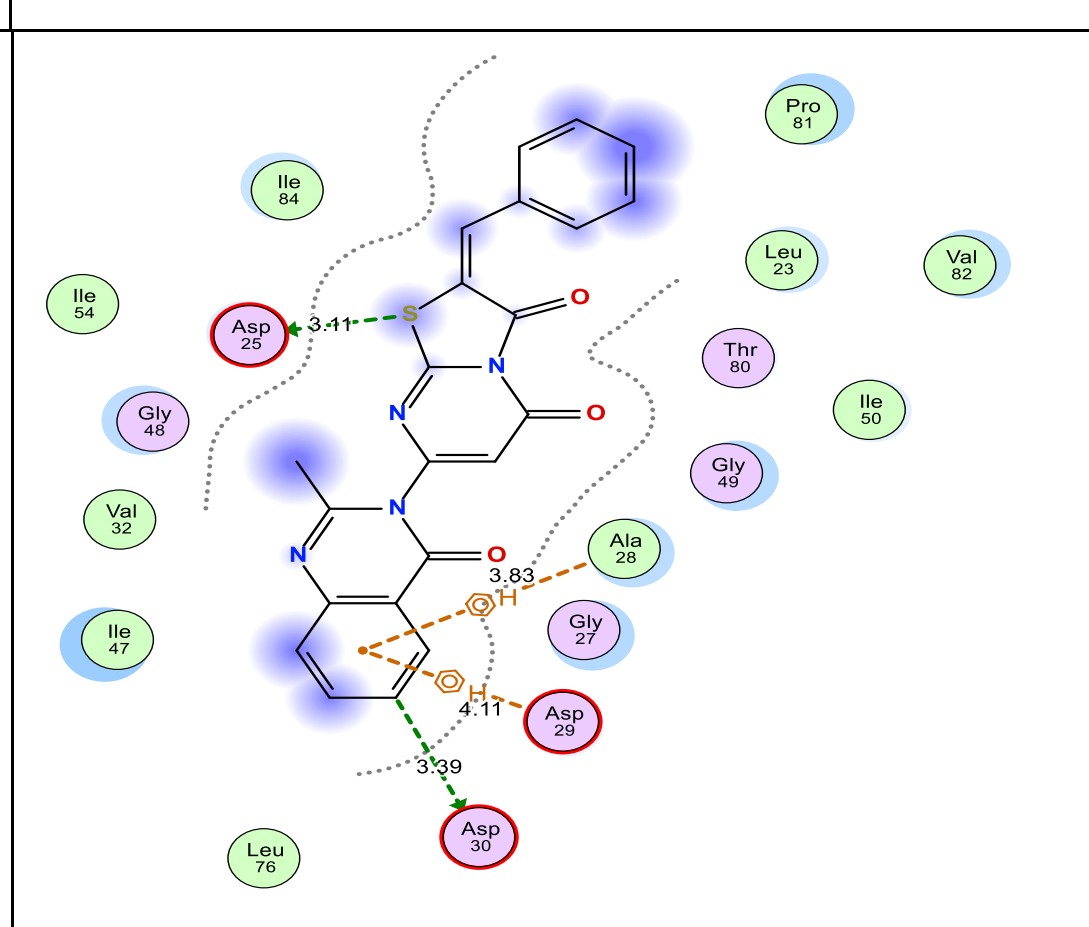 Pro 81 · Ile 84 · Leu 23 · Val 82 · Ile 54 · Asp 25 · 3.41 · Thr 80 · Ile 50 · Gly 48 · Gly 49 · Val 32 · Ala 28 · 3.83 · Gly 27 · Ile 47 · 4.11 · Asp 29 · 3.39 · Asp 30 · Leu 76	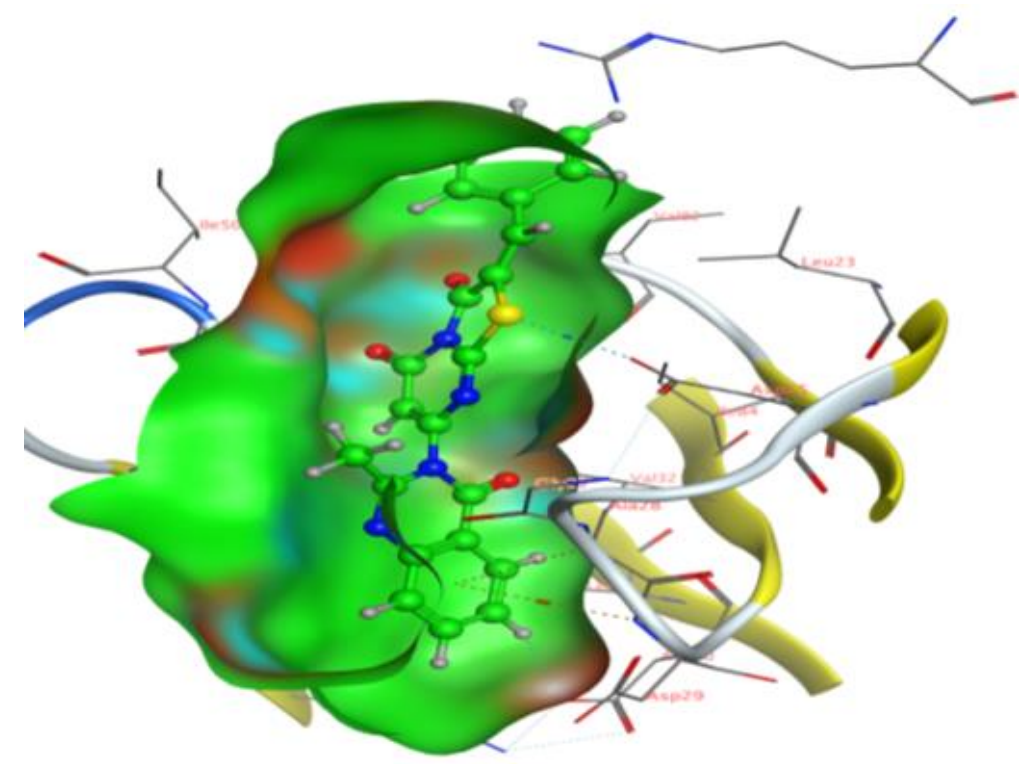

11b

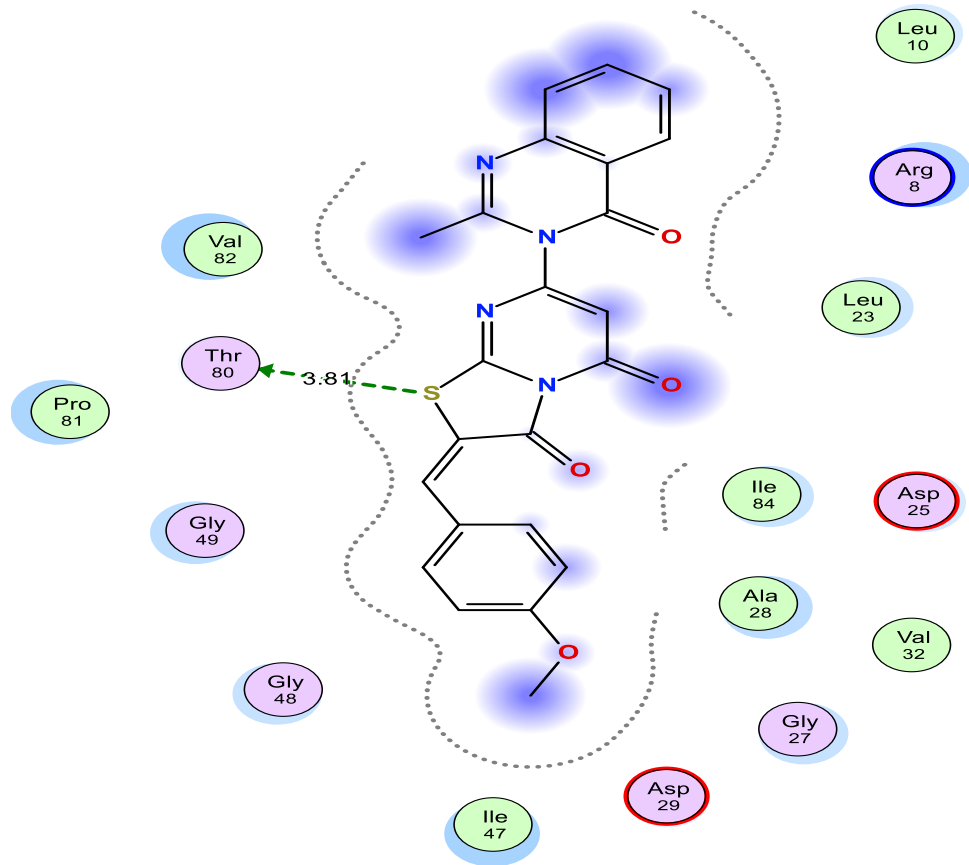

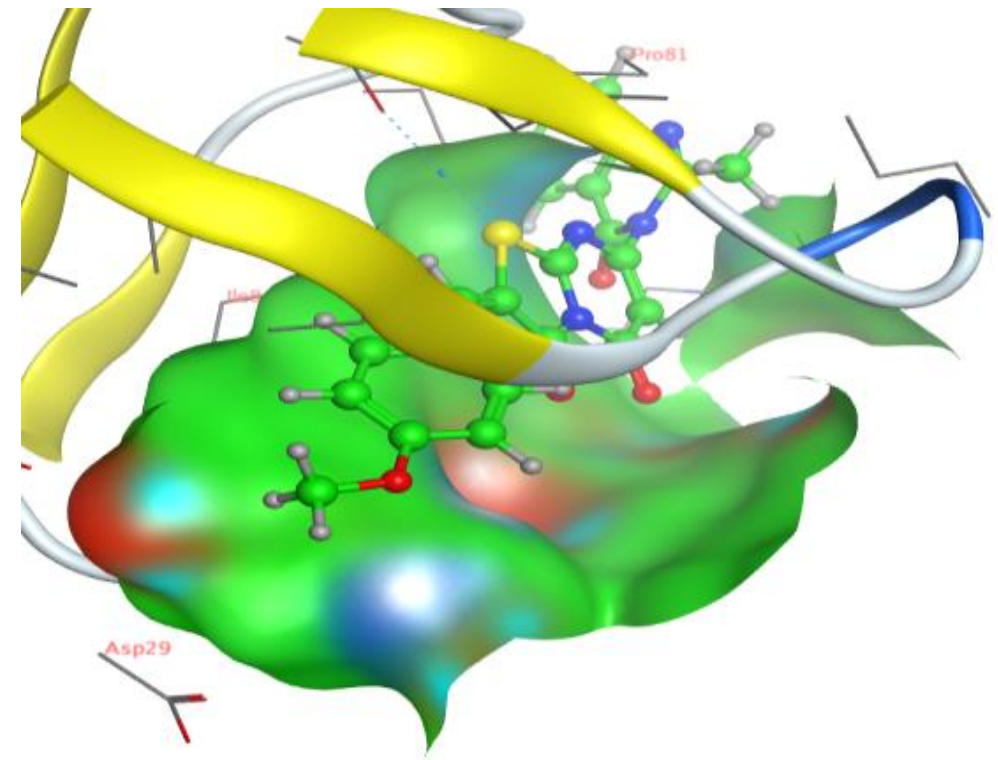

11c

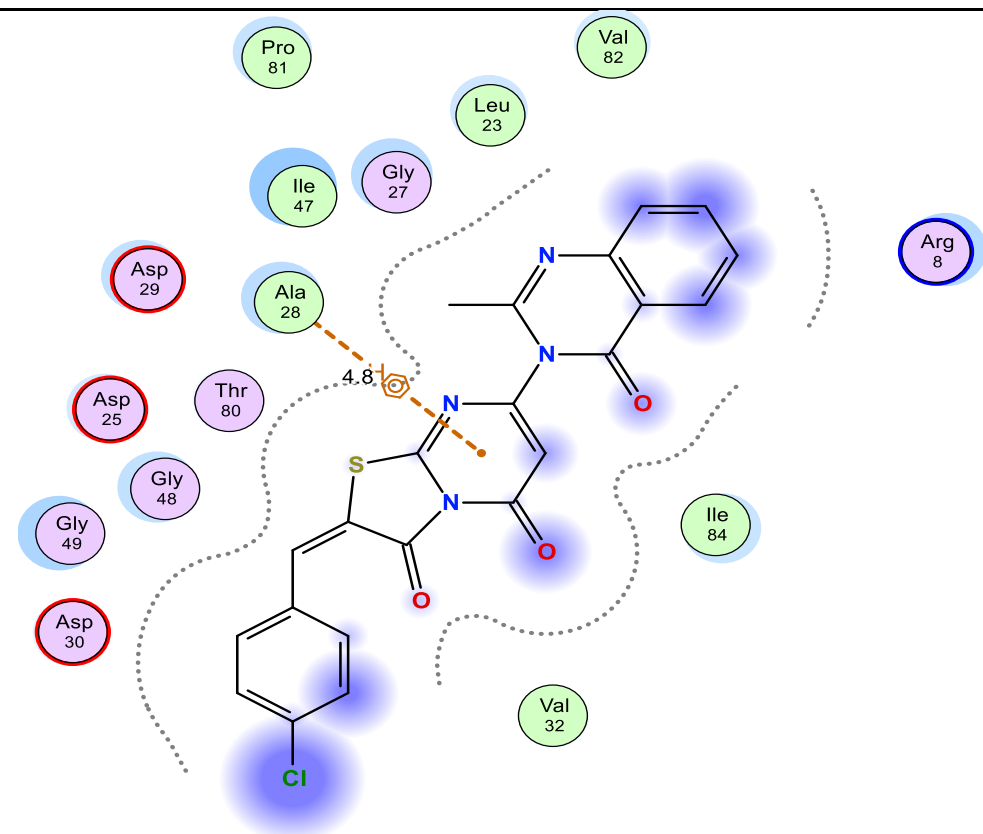

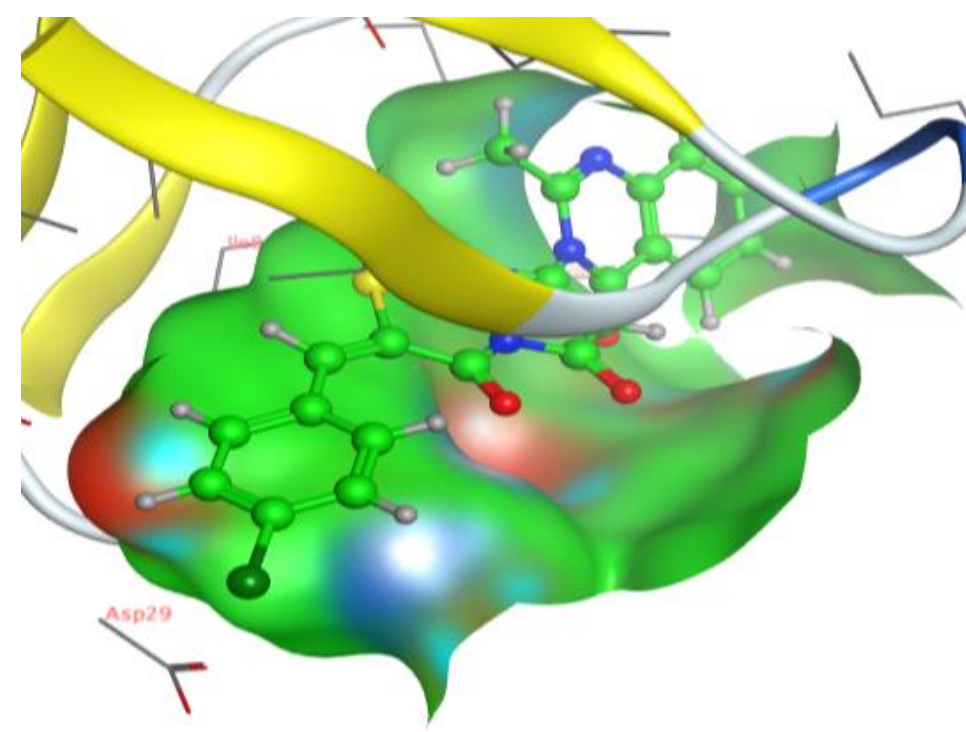

11d

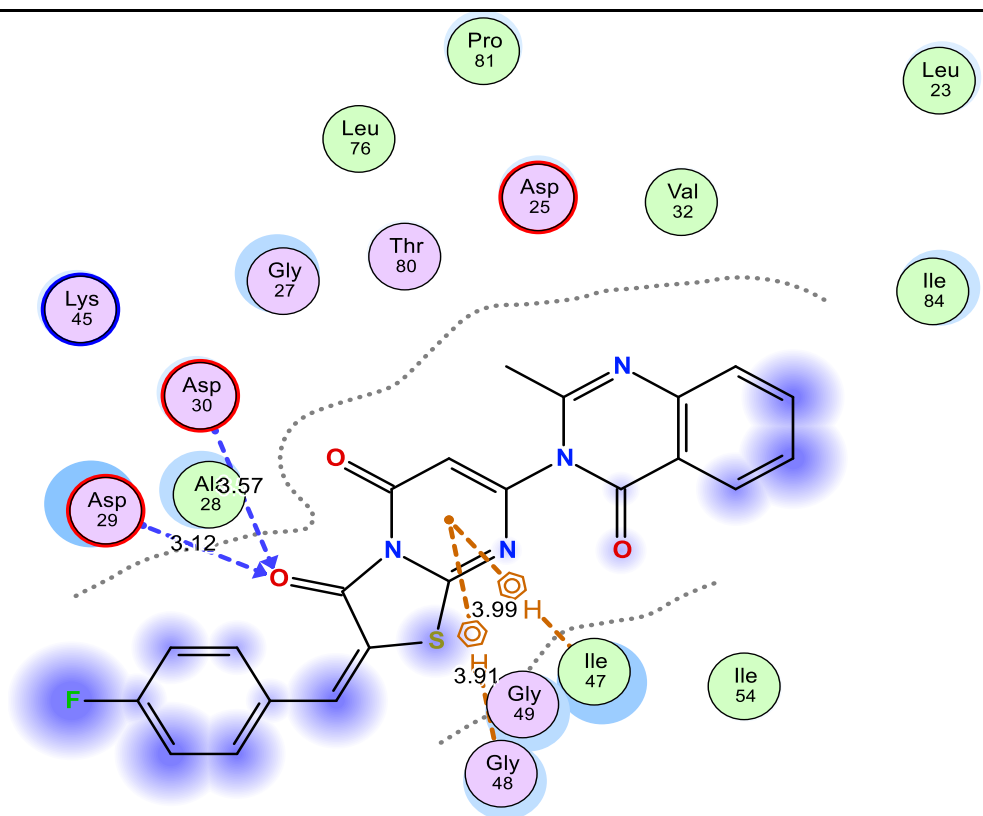

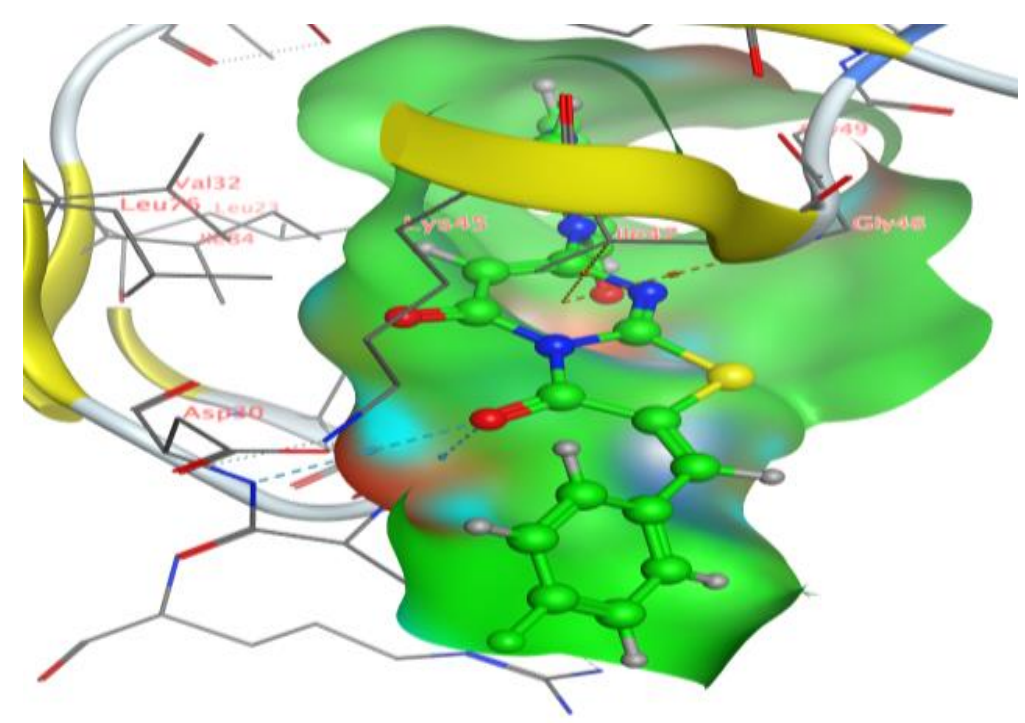

11e

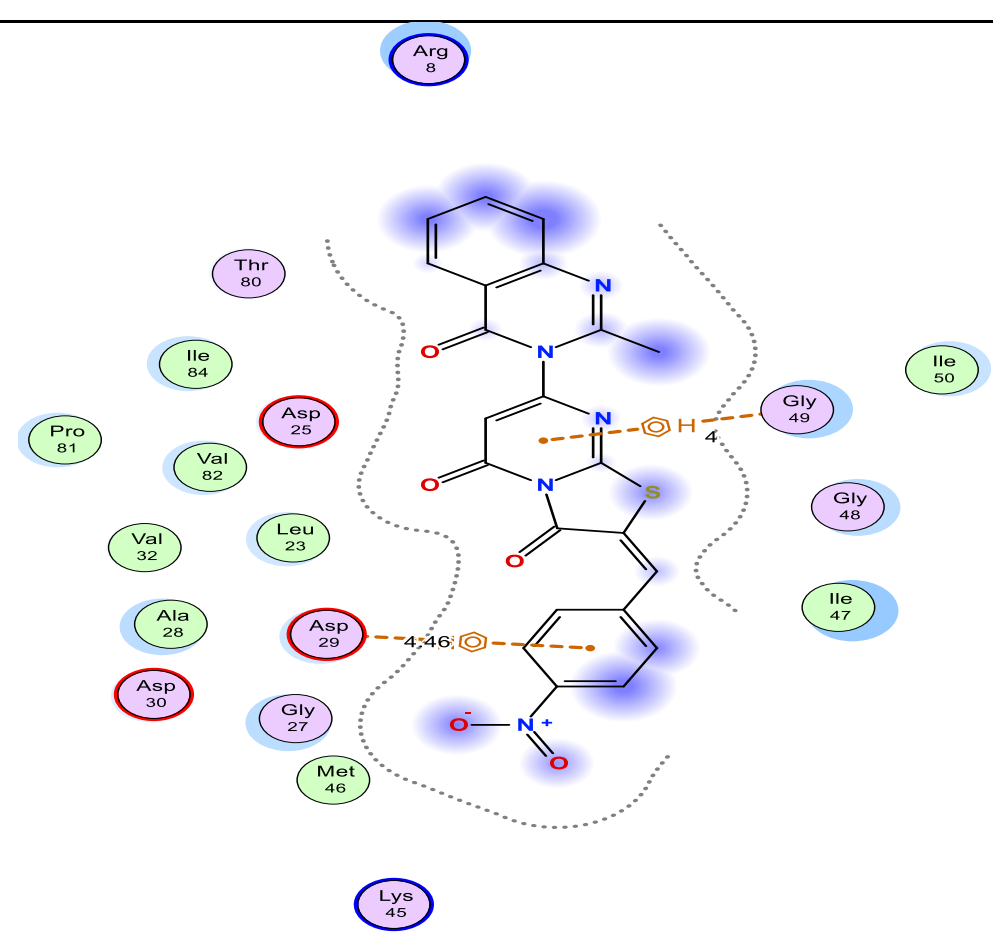

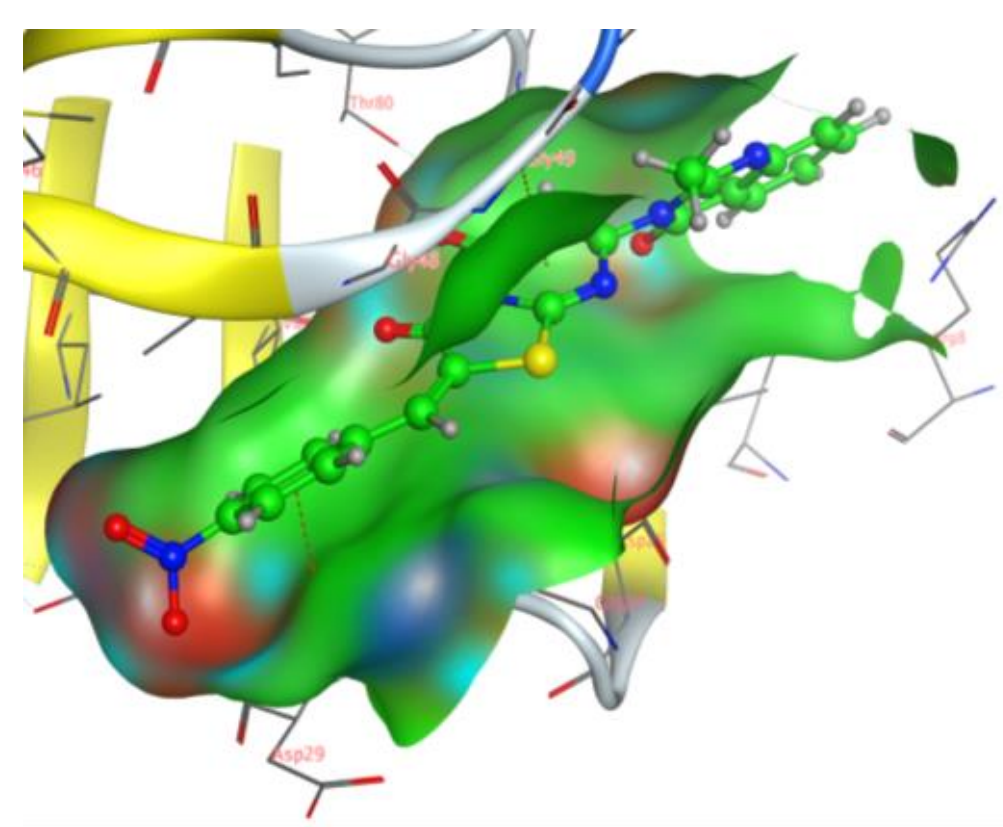

**11f**	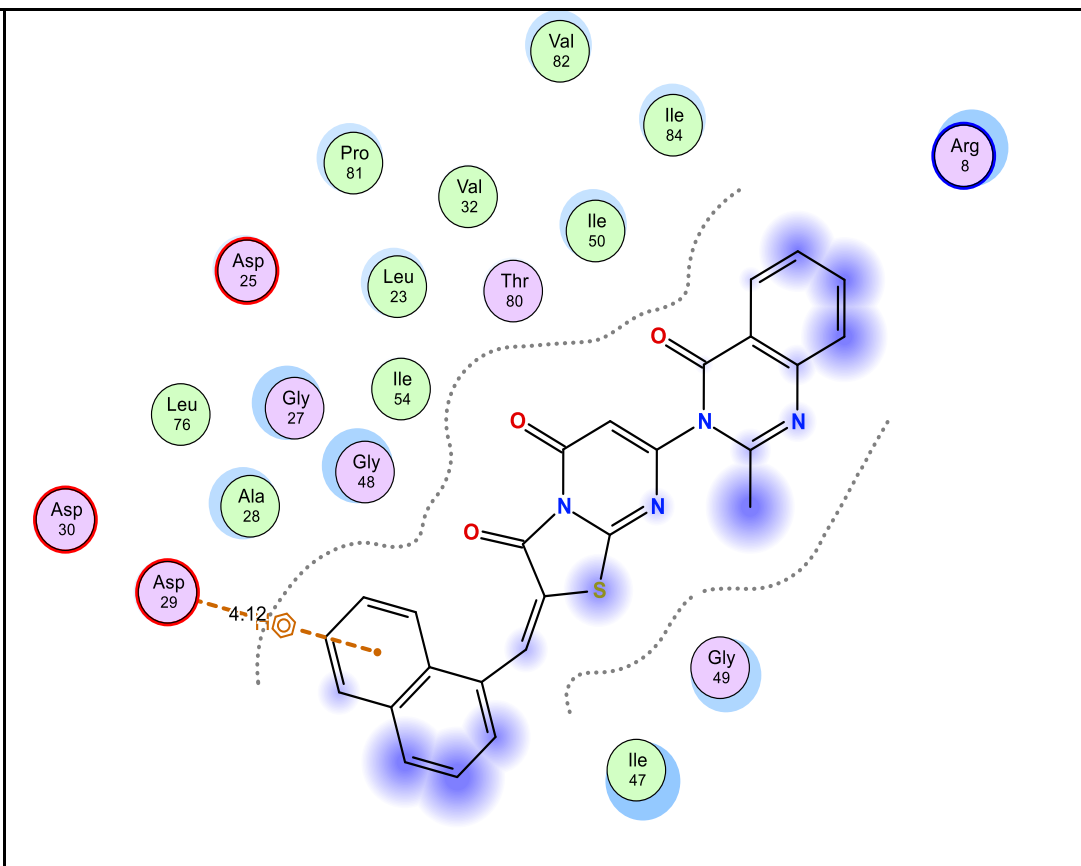	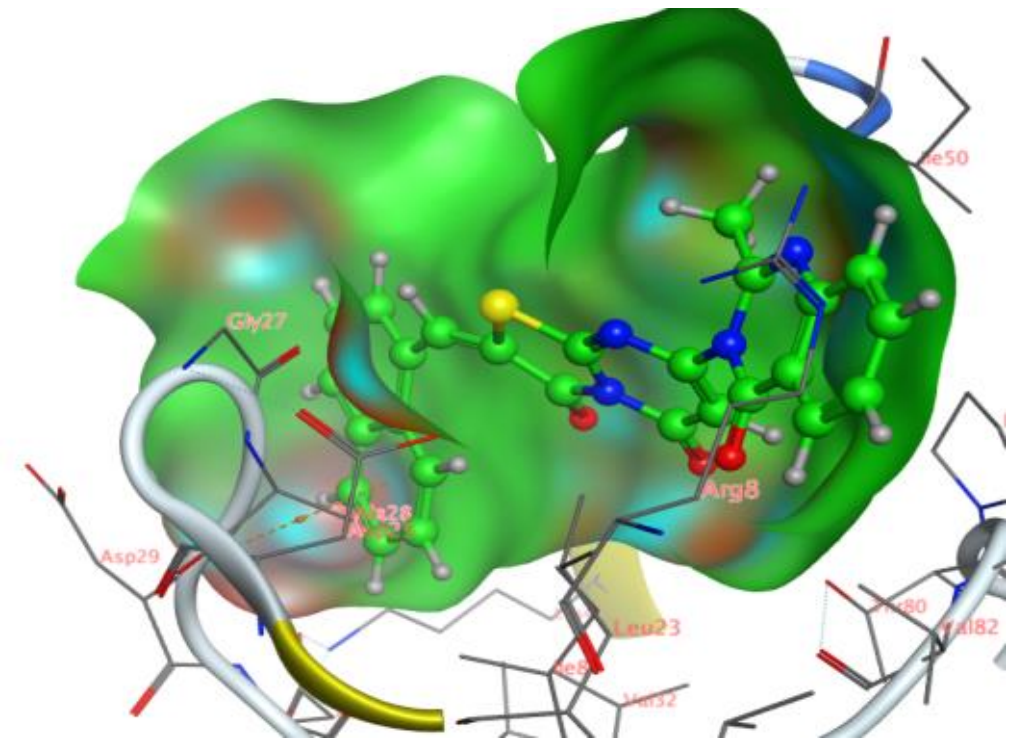
**Zidovudine**	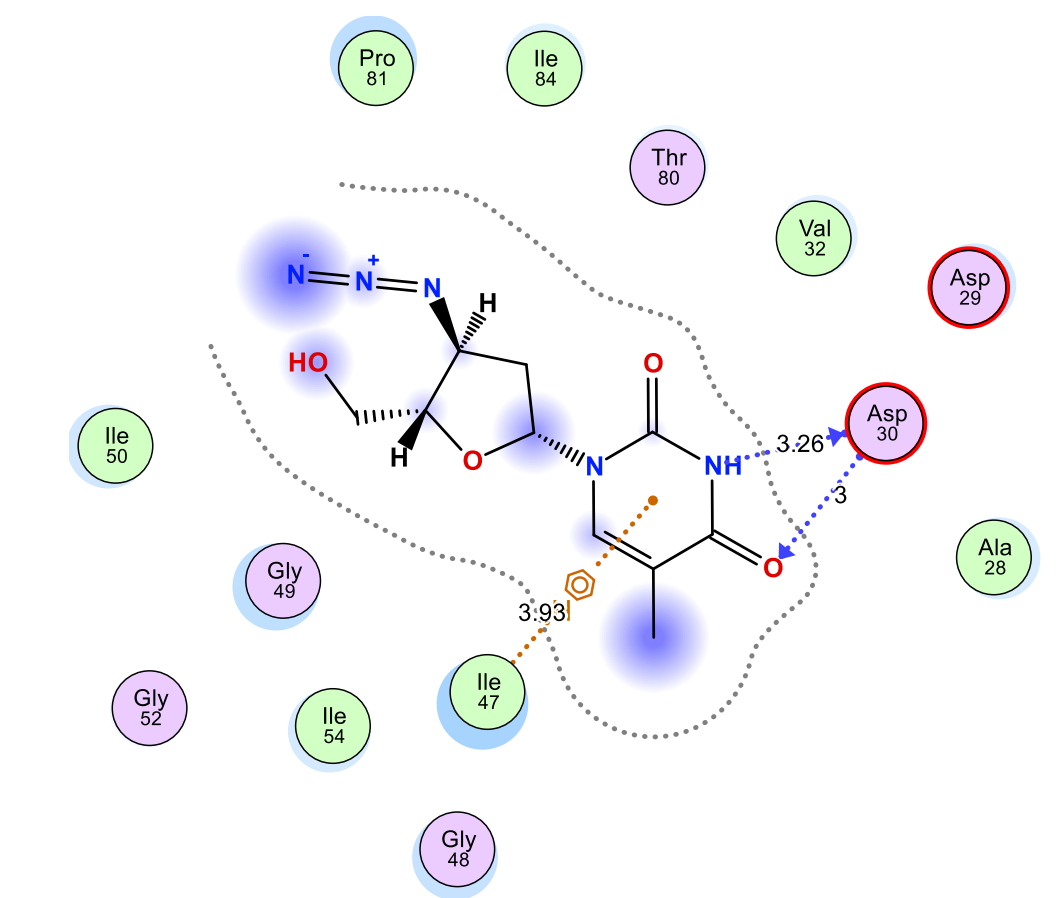	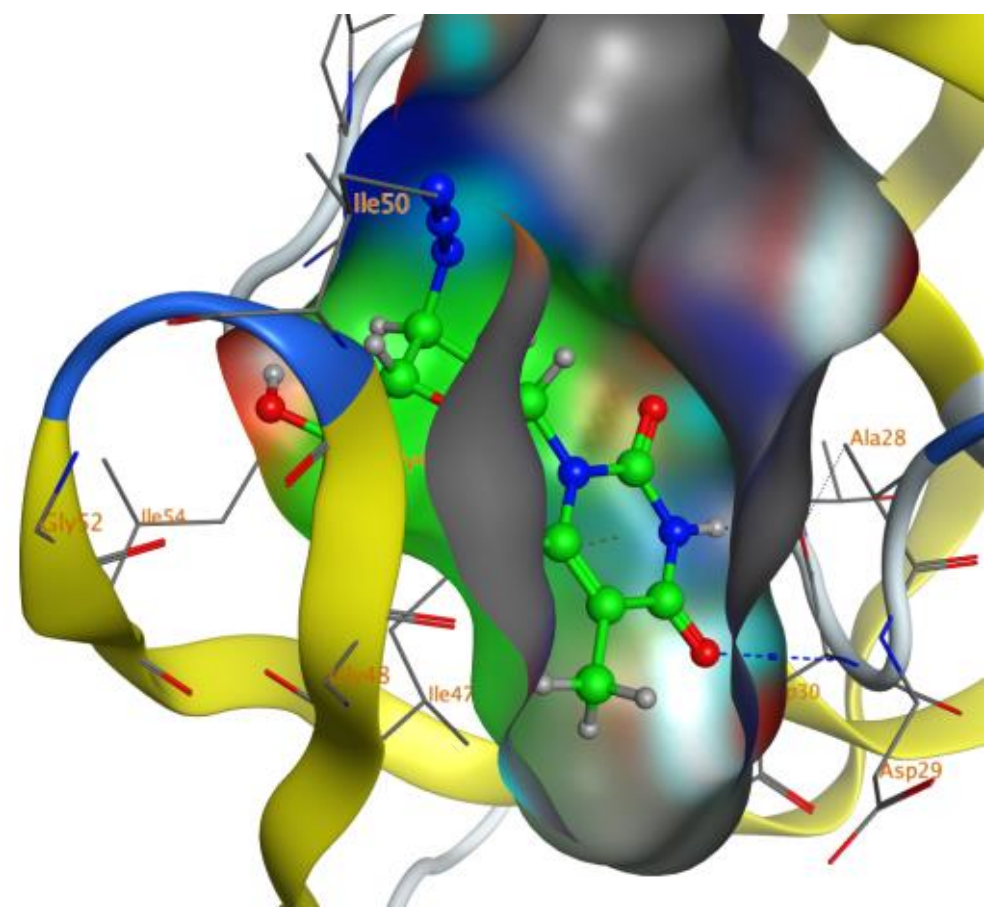

Table S2: Bioavailability radar of the compounds **7a-f** and **11a-f**.

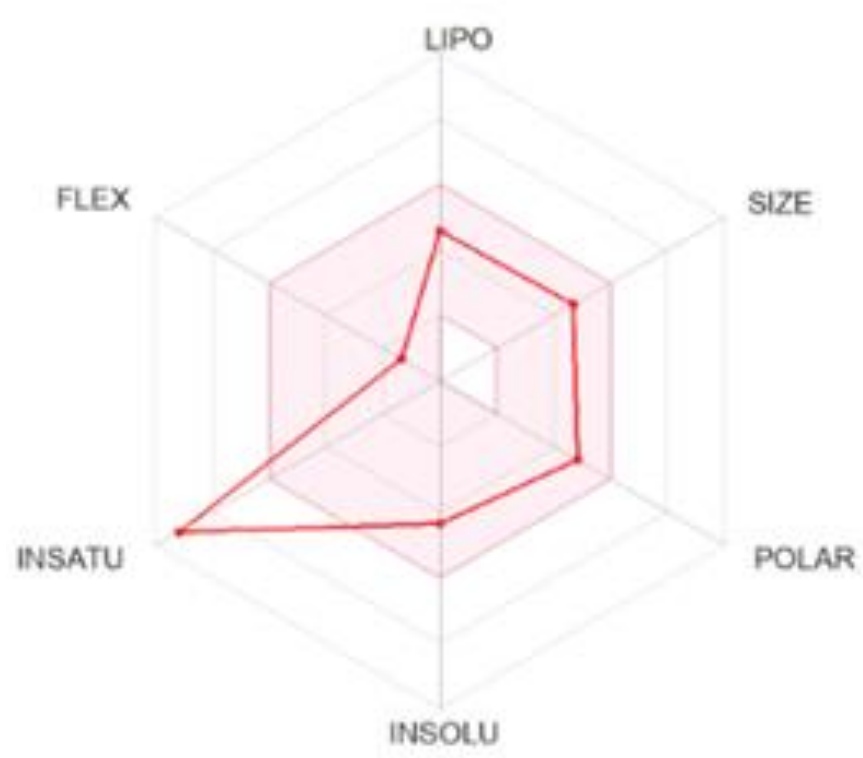	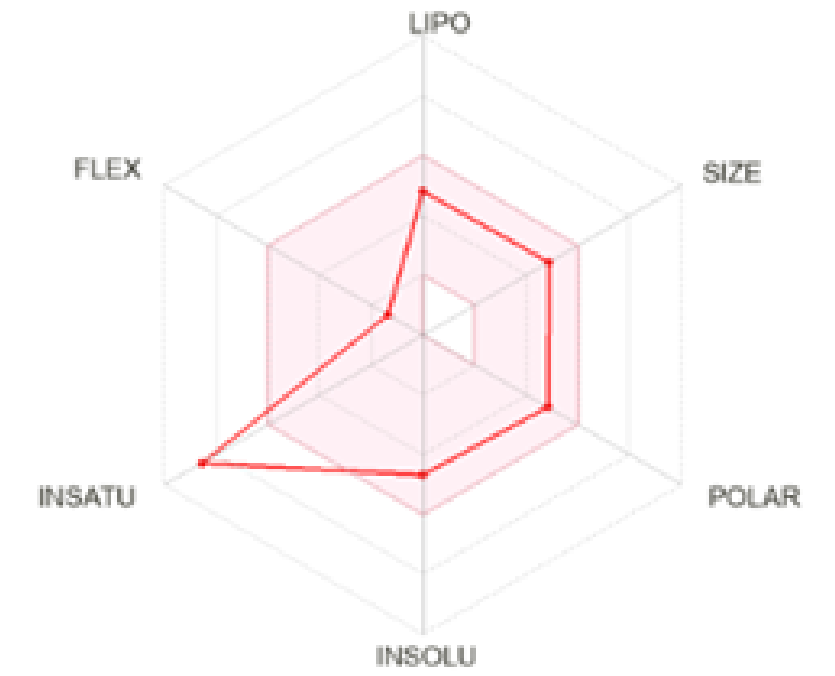	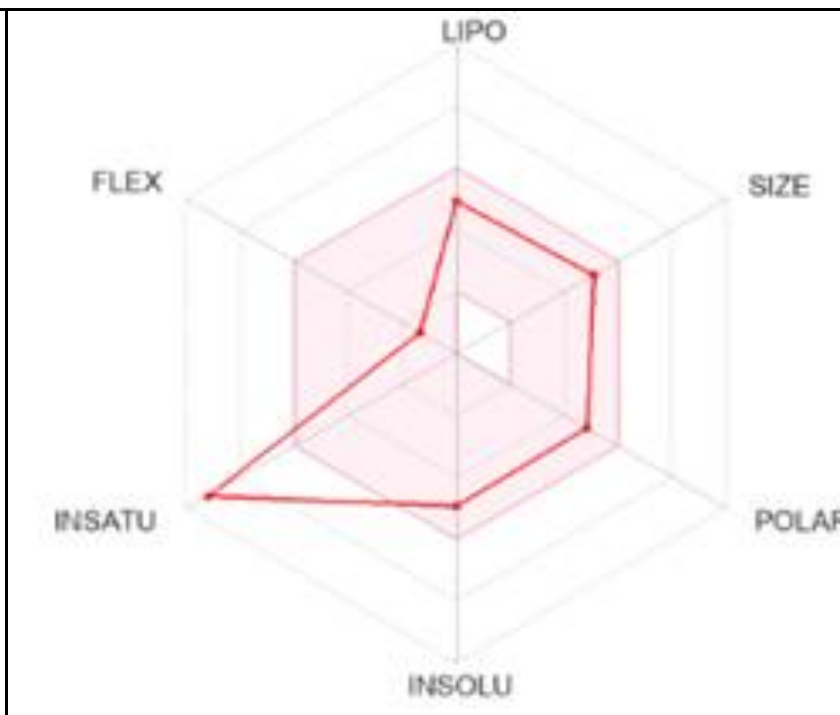
**7a**	**7b**	**7c**
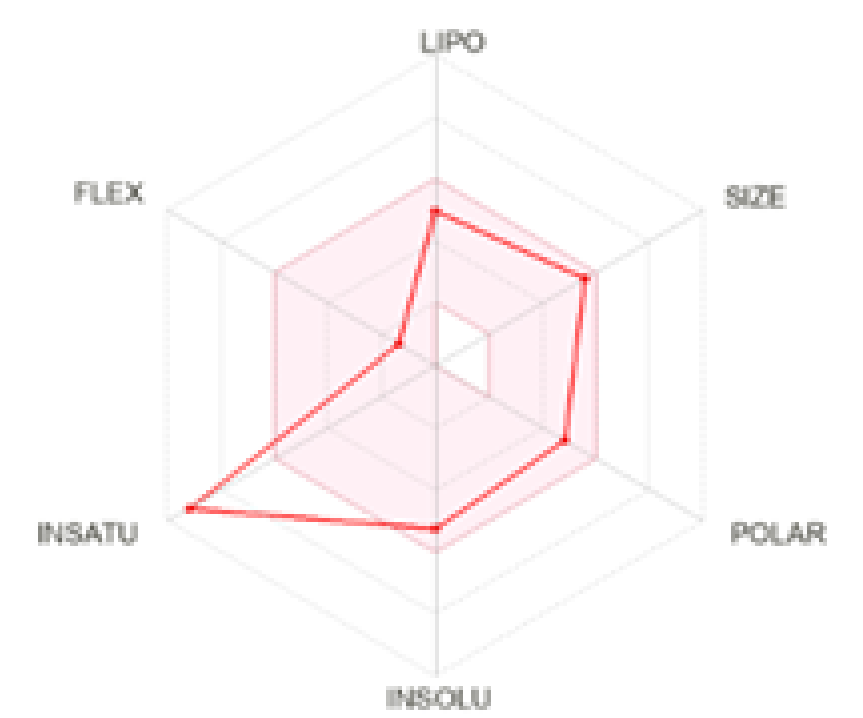	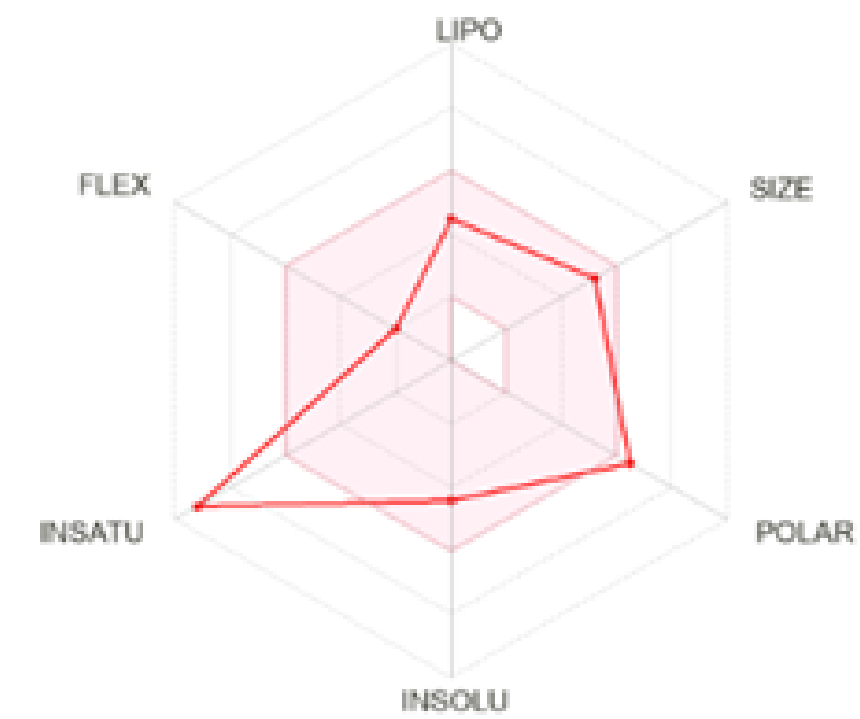	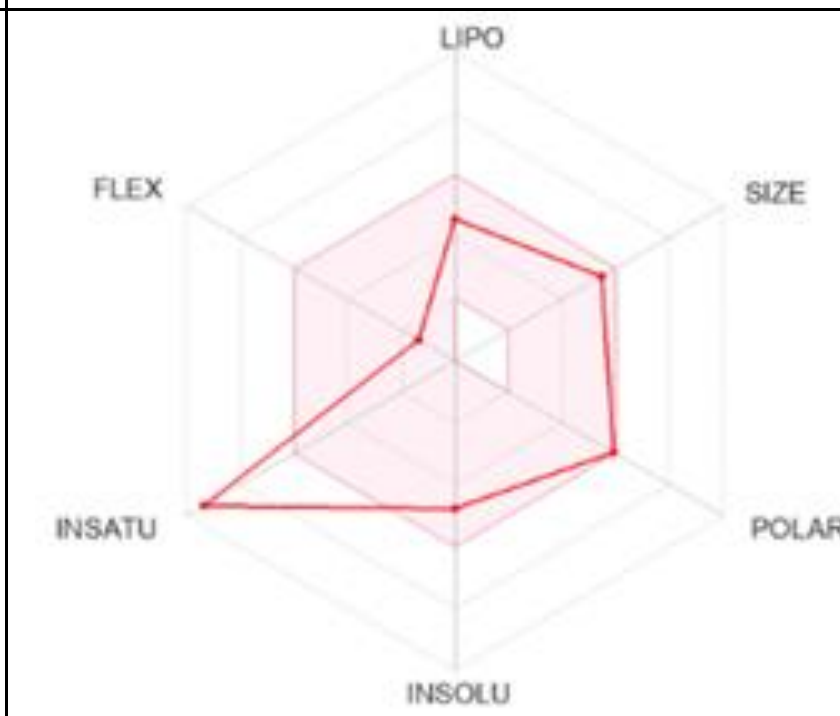
**7d**	**7e**	**7f**
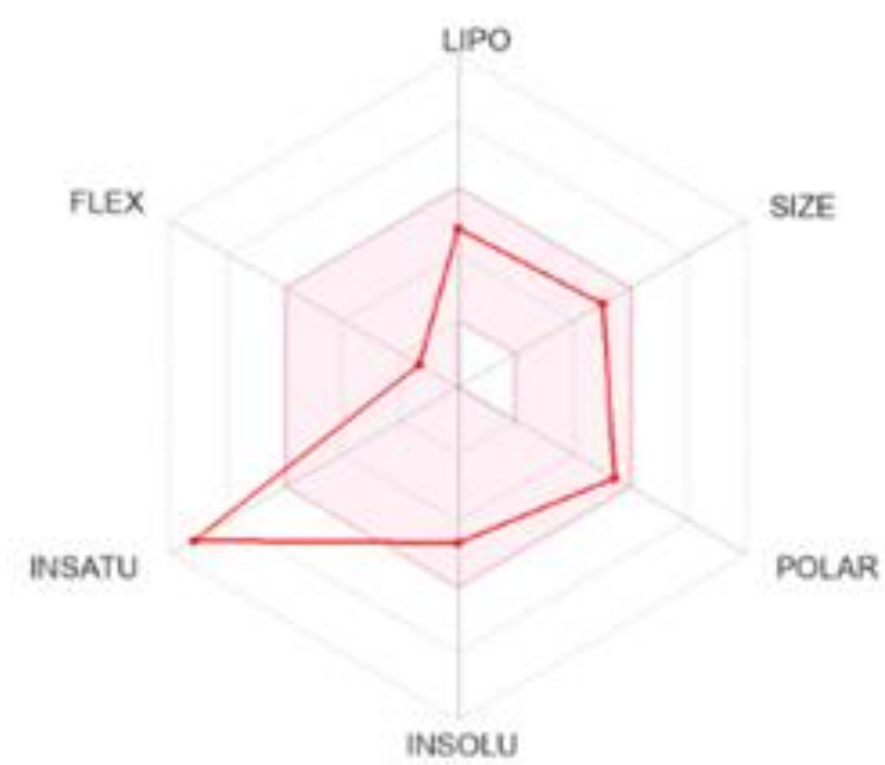	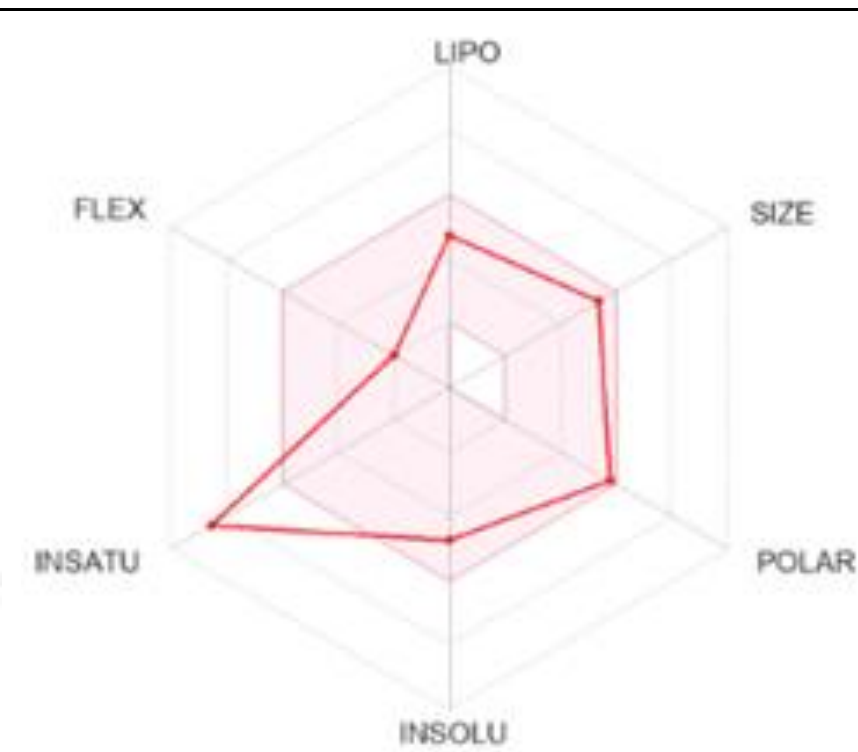	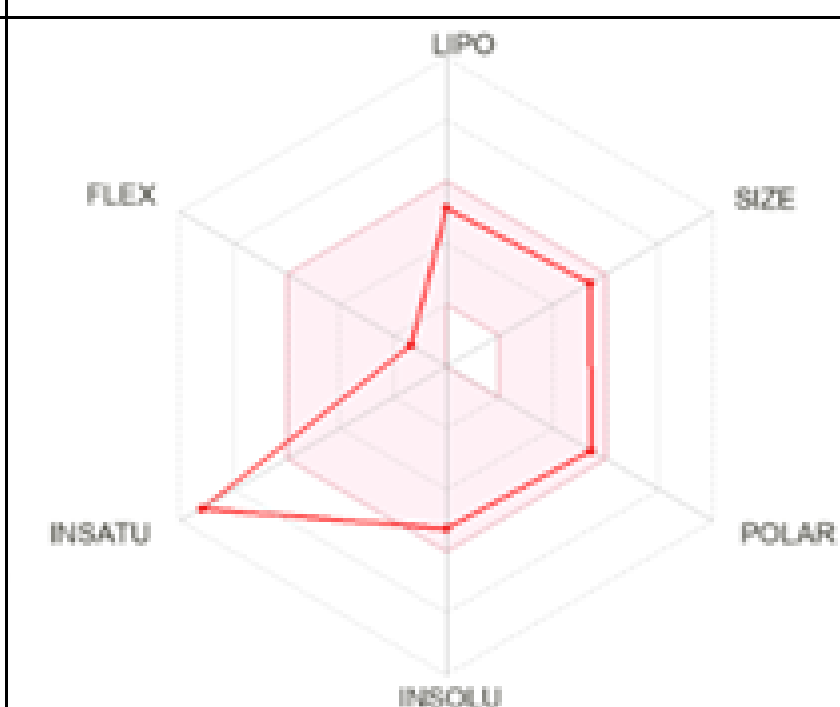
**11a**	**11b**	**11c**
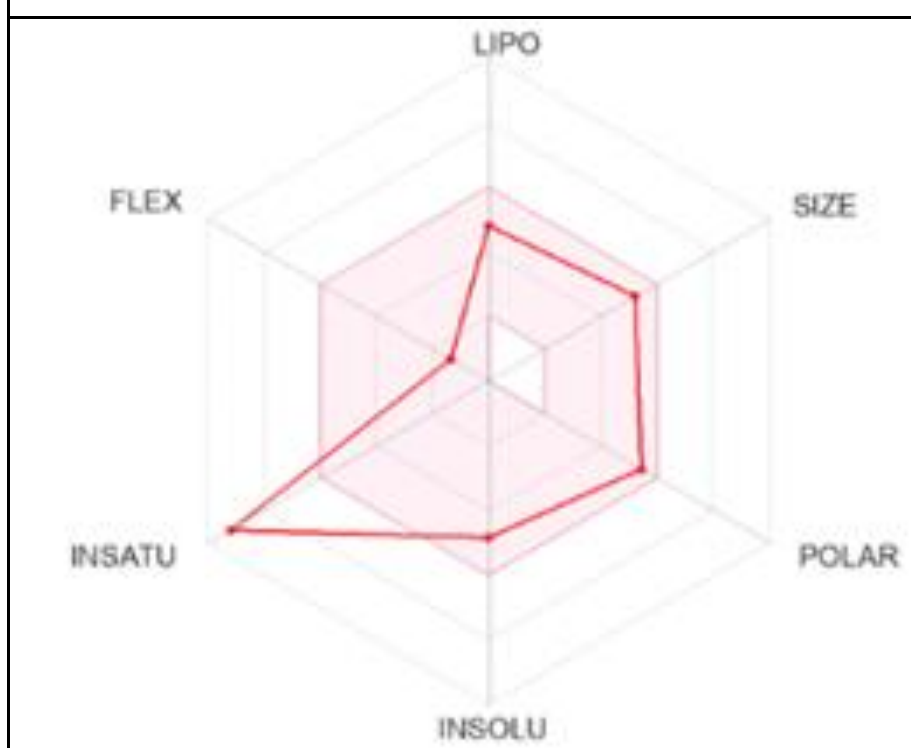	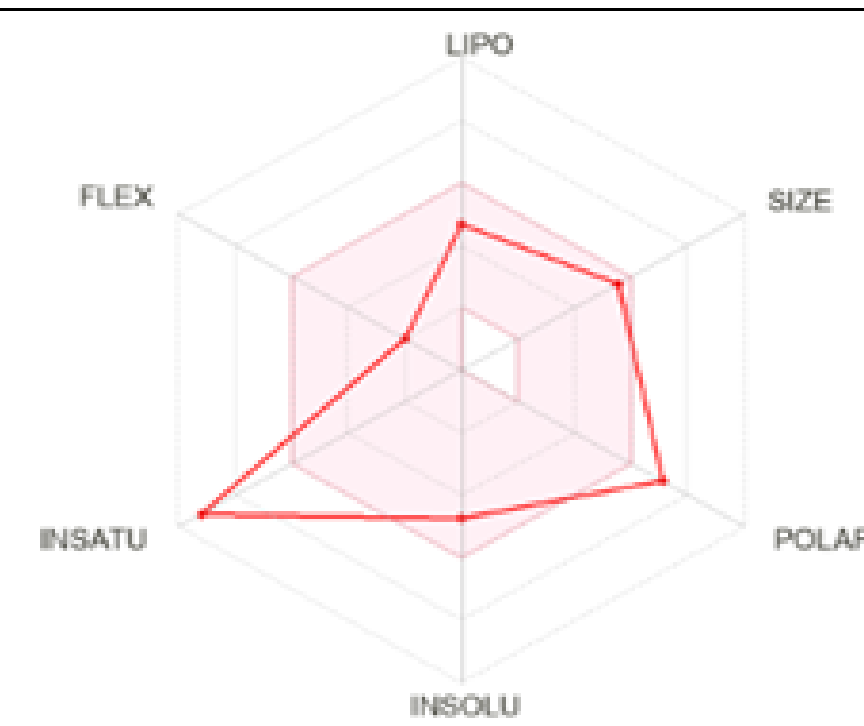	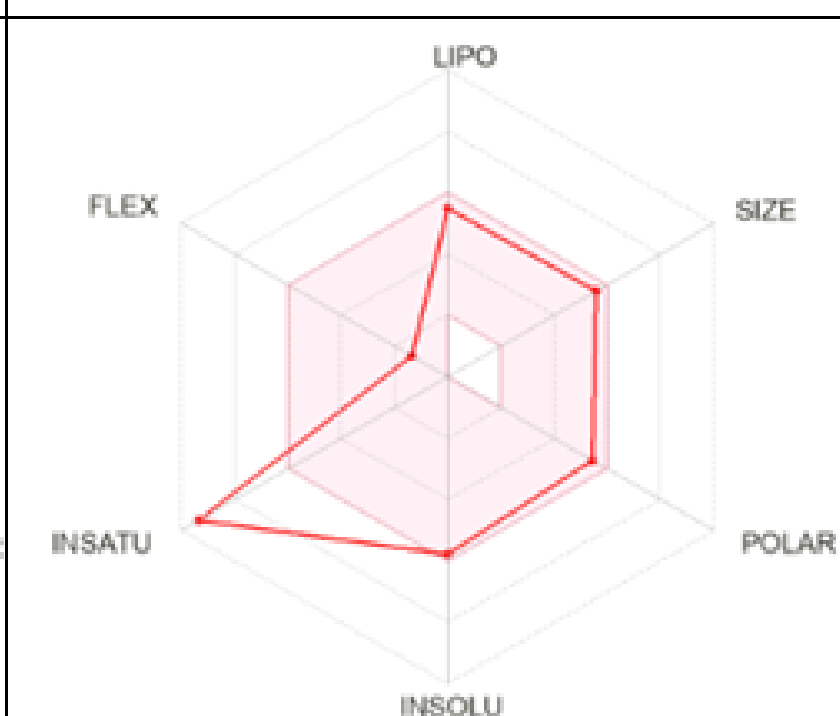
**11d**	**11e**	**11f**

HIV-1 RT inhibitory activities of compounds **7b**, **7e**, **7f**, **11b**, **11e**, **11f** and Zidovudine

s	sample		HIV-RT
	**Compound**	**MWt**	**IC_{50} ug/mL**
**1**	**7f**	**454**	**2.0074±0.086**
**2**	**11b**	**444**	**6.2751±0.269**
**3**	**11f**	**464**	**0.9005±0.034**
**4**	**7b**	**400**	**1.5336±0.066**
**5**	**7e**	**431**	**5.5463±0.238**
**6**	**11e**	**459**	**0.599±0.026**
*******	**Zidovudine**	**267**	**0.2024±0.009**

Detailed Results: HIV RT

[redacted]												
code	IC50	conc	log	%inh	ΔT	ΔOD	slope	K.Activity	EC	OD1	OD2	OD3
**7f**		50	1.7	84.3	30	0.2978	0.063362	18.7998956	120	0.295	0.300	0.298
		10	1	58.9	30	0.78135	0.063362	49.3258278	120	0.774	0.787	0.783
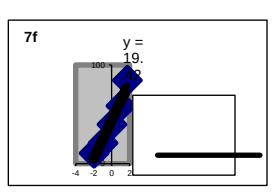		1	0	39.5	30	1.14981	0.063362	72.5867157	120	1.139	1.158	1.152
		0.1	-1	21.2	30	1.49808	0.063362	94.5730343	120	1.484	1.509	1.501
		0.01	-2	10.8	30	1.69493	0.063362	107.000084	120	1.679	1.707	1.698
EC				0	30	1.90087	0.063362	120	120	1.883	1.915	1.905
		[redacted]		[redacted]			[redacted]					
code	IC50	conc.ng/ml	log conc	%inh	ΔT	ΔRFU	slope	K.Activity	EC			
11B		50	1.7	81.5	30	0.35231	0.063362	22.2412325	120	0.349	0.355	0.353
		10	1	51.4	30	0.92368	0.063362	58.3115407	120	0.915	0.931	0.926
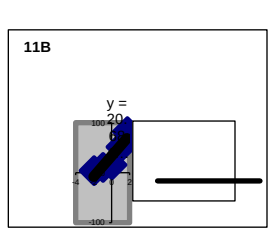		1	0	15.4	30	1.60812	0.063362	101.519436	120	1.593	1.620	1.611
		0.1	-1	9.93	30	1.7121	0.063362	108.083468	120	1.696	1.725	1.716
		0.01	-2	3.08	30	1.84232	0.063362	116.304439	120	1.825	1.856	1.846
EC				0	30	1.90087	0.063362	120	120	1.883	1.915	1.905
		[redacted]		[redacted]			[redacted]					
code	IC50	conc.ng/ml	log conc	%inh	ΔT	ΔRFU	slope	K.Activity	EC			
**11f**		50	1.7	87.6	30	0.23521	0.063362	14.8487311	120	0.233	0.237	0.236

		10	1	71	30	0.55118	0.063362	34.795739	120	0.546	0.555	0.552
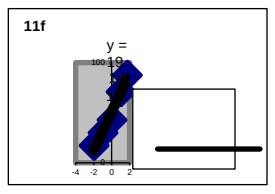		1	0	42.5	30	1.09227	0.063362	68.9541934	120	1.082	1.100	1.094
		0.1	-1	30.1	30	1.32849	0.063362	83.866653	120	1.316	1.338	1.331
		0.01	-2	17.3	30	1.57178	0.063362	99.2252118	120	1.557	1.583	1.575
EC				0	30	1.90087	0.063362	120	120	1.883	1.915	1.905
		[redacted]		[redacted]			[redacted]					

code	IC50	conc.ng/ml	log conc	%inh	ΔT	ΔRFU	slope	K.Activity	EC			
**7B**		50	1.7	86.1	30	0.26449	0.063362	16.6968565	120	0.262	0.266	0.265
		10	1	58.9	30	0.78135	0.063362	49.3258278	120	0.774	0.787	0.783
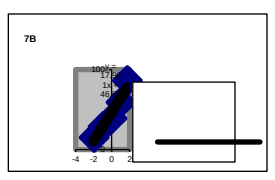		1	0	38.9	30	1.16192	0.063362	73.3514572	120	1.151	1.171	1.164
		0.1	-1	28.7	30	1.35474	0.063362	85.523593	120	1.342	1.365	1.357
		0.01	-2	15.3	30	1.61014	0.063362	101.646893	120	1.595	1.622	1.613
EC				0	30	1.90087	0.063362	120	120	1.883	1.915	1.905
		[redacted]		[redacted]			[redacted]					

code	IC50	conc.ng/ml	log conc	%inh	ΔT	ΔRFU	slope	K.Activity	EC			
**7e**		50	1.7	79.6	30	0.38865	0.063362	24.535457	120	0.385	0.392	0.389
		10	1	51.4	30	0.92469	0.063362	58.3752691	120	0.916	0.932	0.927
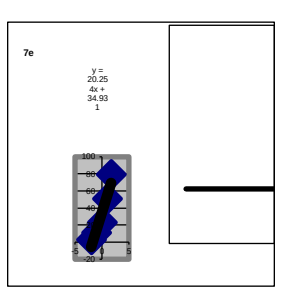		1	0	23.6	30	1.45266	0.063362	91.7052536	120	1.439	1.463	1.456
		0.1	-1	11.1	30	1.68989	0.063362	106.681442	120	1.674	1.702	1.693
		0.01	-2	2.97	30	1.84434	0.063362	116.431896	120	1.827	1.858	1.848
EC				0	30	1.90087	0.063362	120	120	1.883	1.915	1.905
		[redacted]		[redacted]			[redacted]					

code	IC50	conc.ng/ml	log conc	%inh	ΔT	ΔRFU	slope	K.Activity	EC			
**11e**		50	1.7	89.6	30	0.19685	0.063362	12.4270497	120	0.195	0.198	0.197
		10	1	76.3	30	0.45023	0.063362	28.422893	120	0.446	0.454	0.451
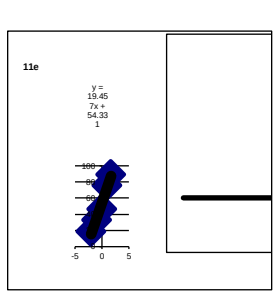		1	0	47.2	30	1.00444	0.063362	63.4098175	120	0.995	1.012	1.006
		0.1	-1	33.3	30	1.26792	0.063362	80.0429454	120	1.256	1.277	1.270
		0.01	-2	19.4	30	1.53241	0.063362	96.7398019	120	1.518	1.544	1.536
EC				0	30	1.90087	0.063362	120	120	1.883	1.915	1.905
		[redacted]		[redacted]			[redacted]					

code	IC50	conc.ng/ml	log conc	%inh	ΔT	ΔRFU	slope	K.Activity	EC

**Zidovudine**	50	1.7	90.9	30	0.17363	0.063362	10.9612951	120		0.172	0.175	0.174
	10	1	84.7	30	0.29174	0.063362	18.4175249	120		0.289	0.294	0.292
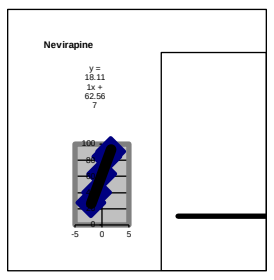	1	0	63.2	30	0.69958	0.063362	44.1638226	120		0.693	0.705	0.701
	0.1	-1	40.4	30	1.13265	0.063362	71.5033318	120		1.122	1.141	1.135
	0.01	-2	28.3	30	1.36382	0.063362	86.0971491	120		1.351	1.374	1.367
EC			0	30	1.90087	0.063362	120	120		1.883	1.915	1.905

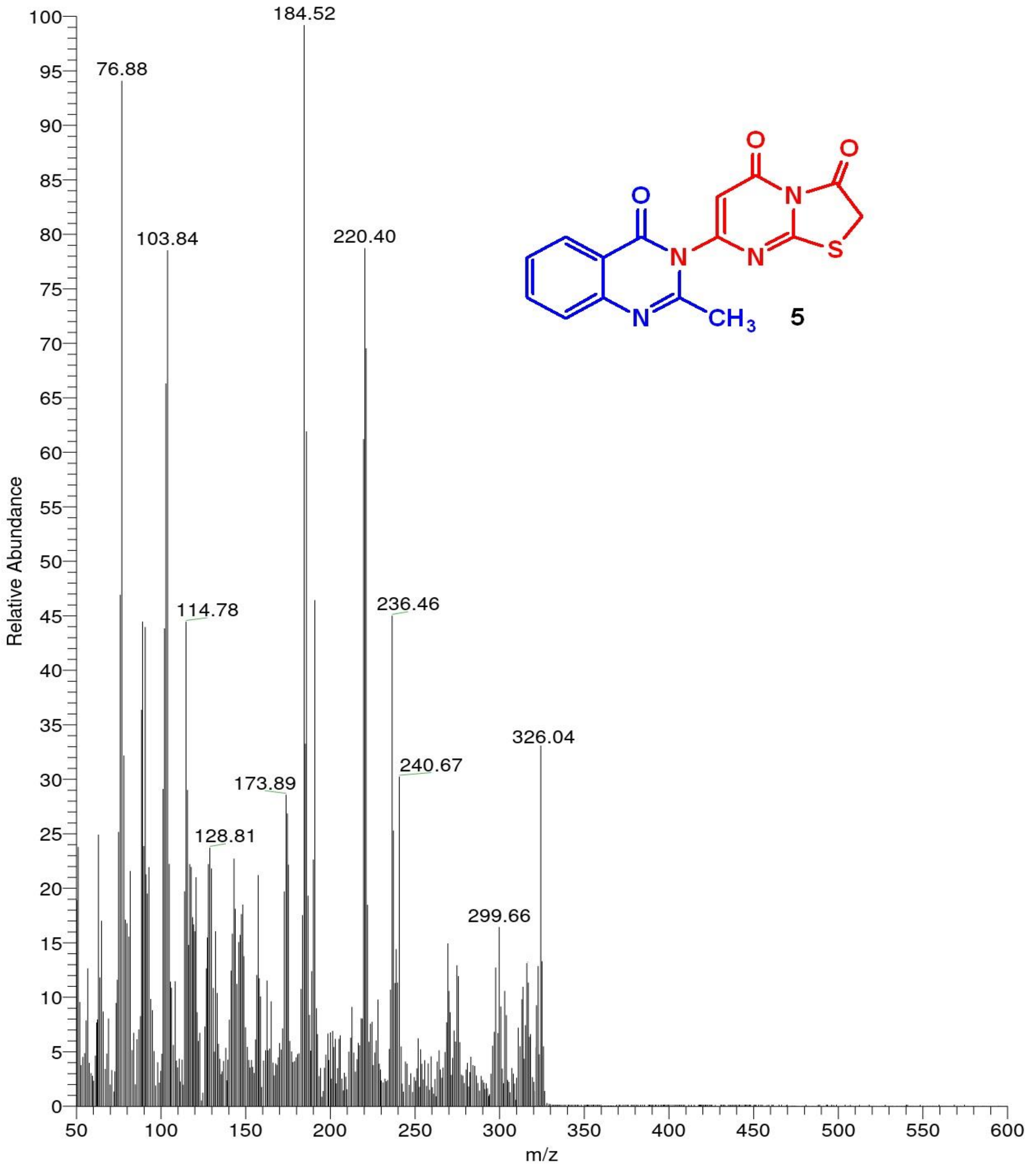

Mass spectra of compound **5**

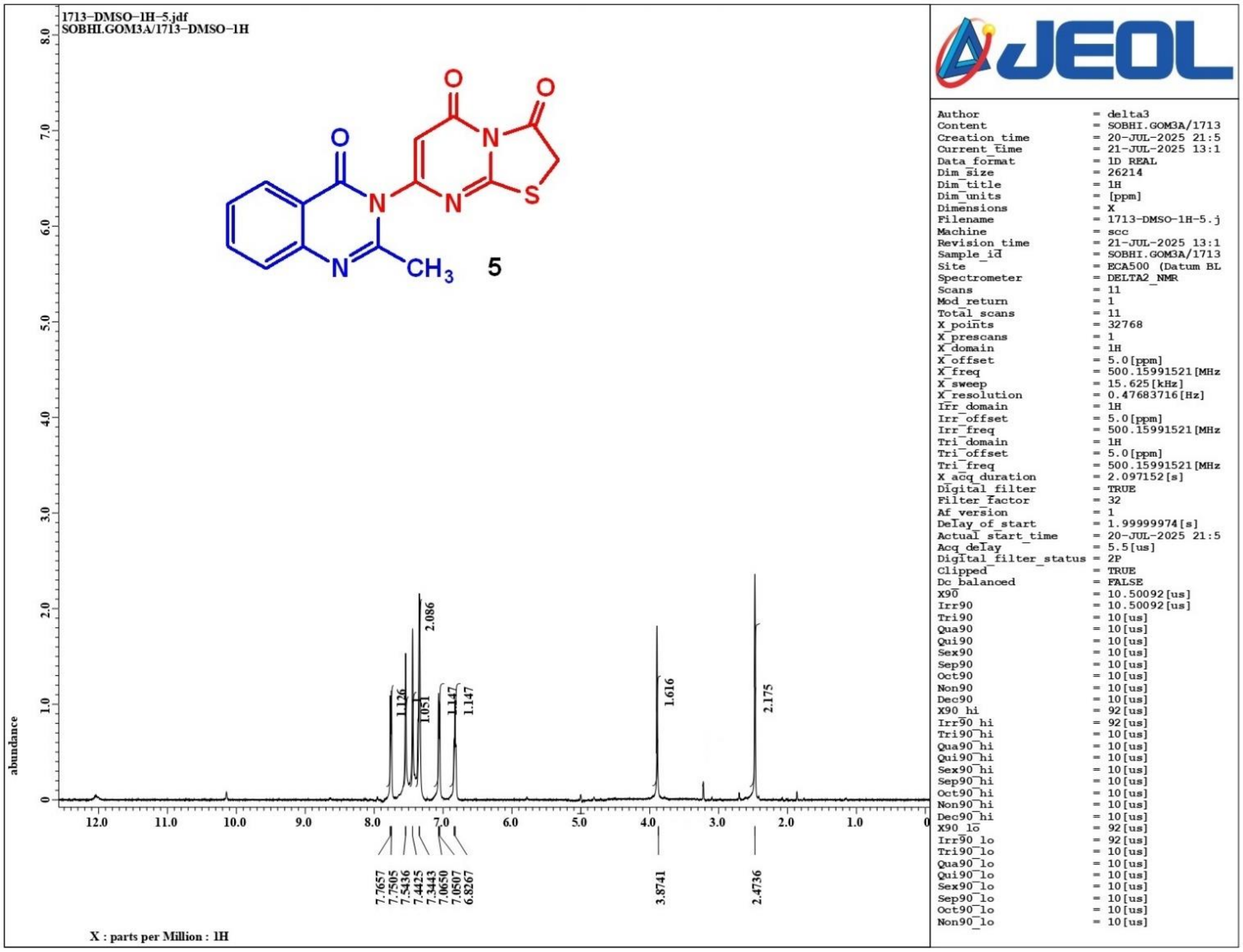

^{1}HNMR spectra of compound **5**

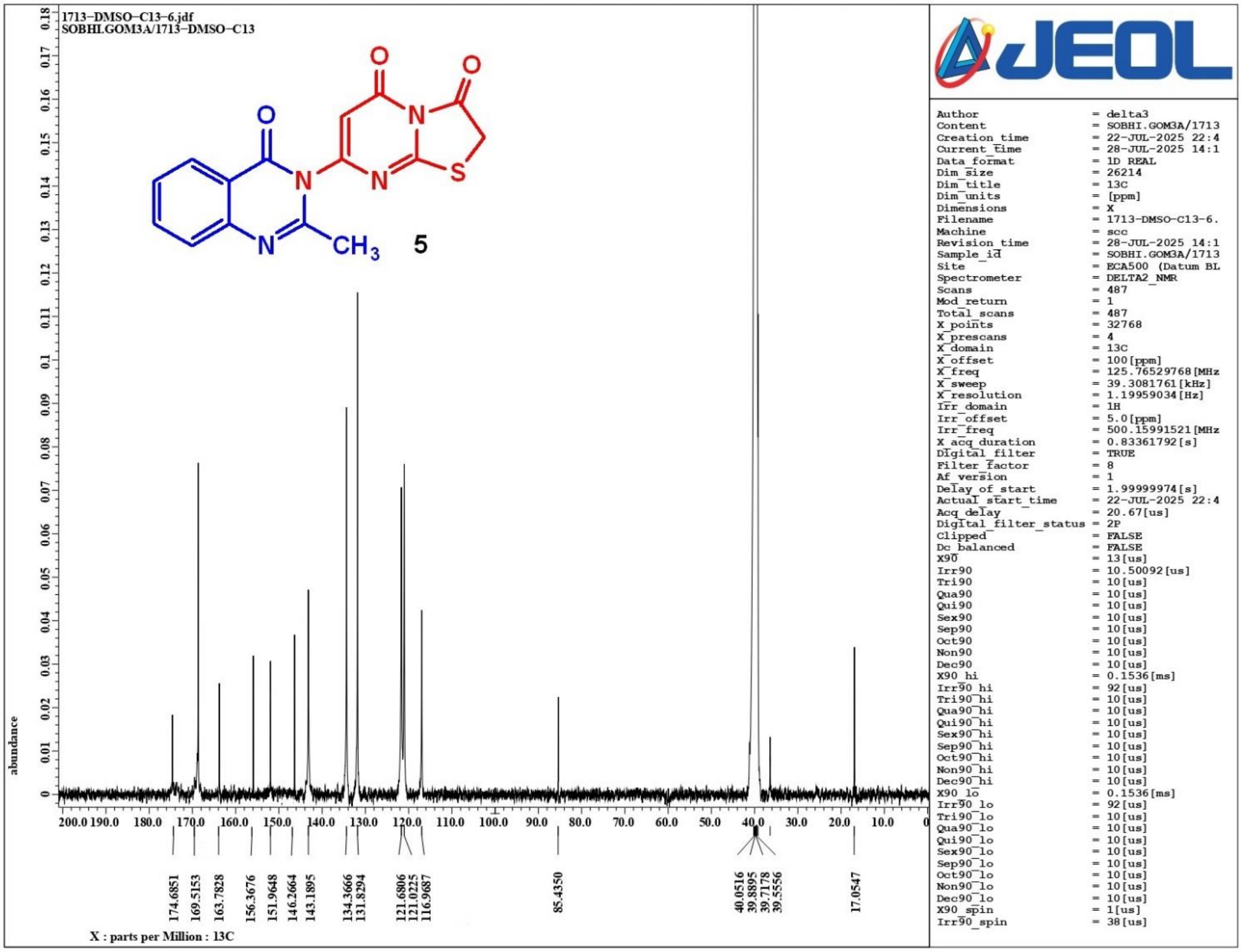

^{13}CNMR spectra of compound **5**

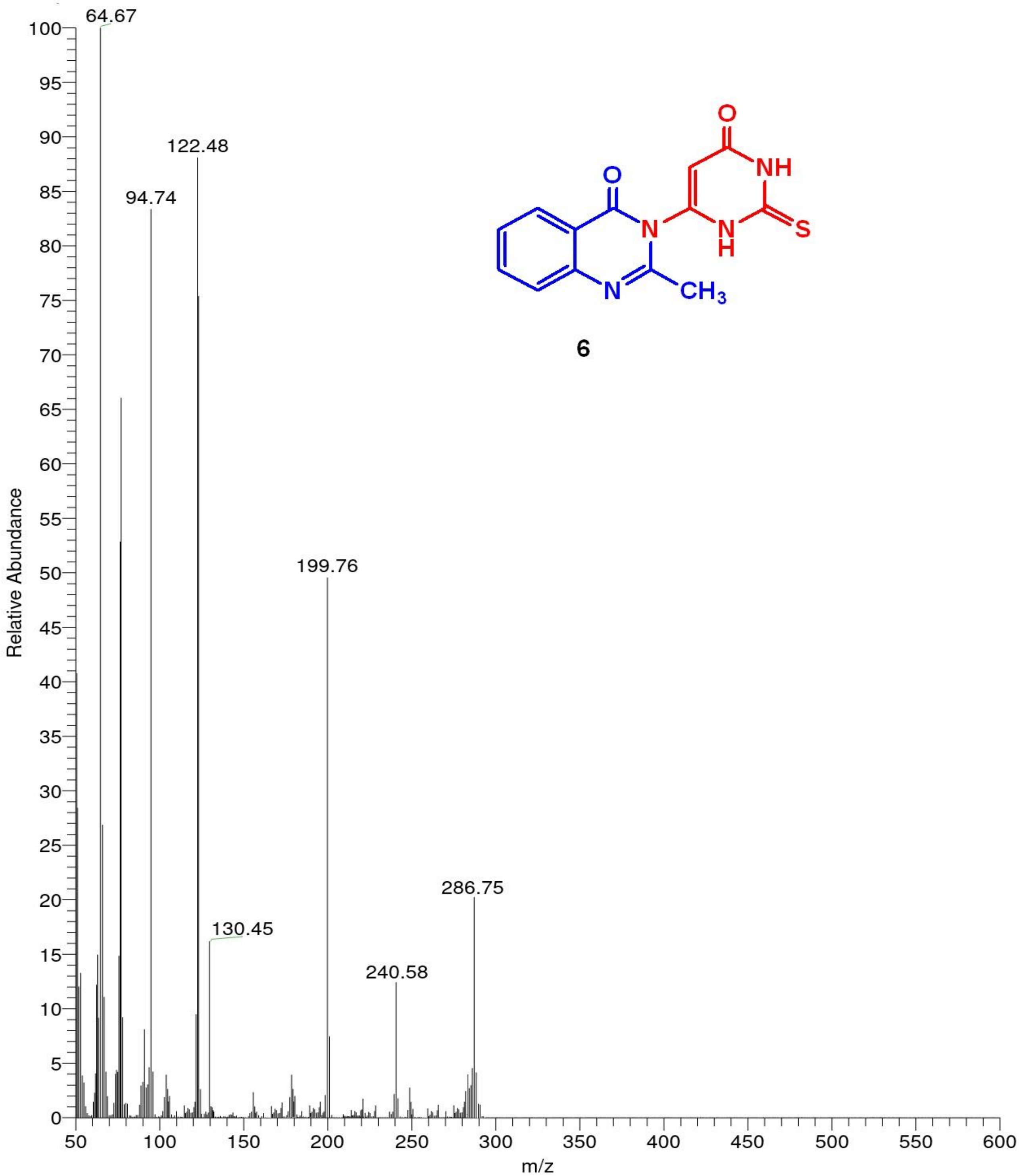

Mass spectra of compound **6**

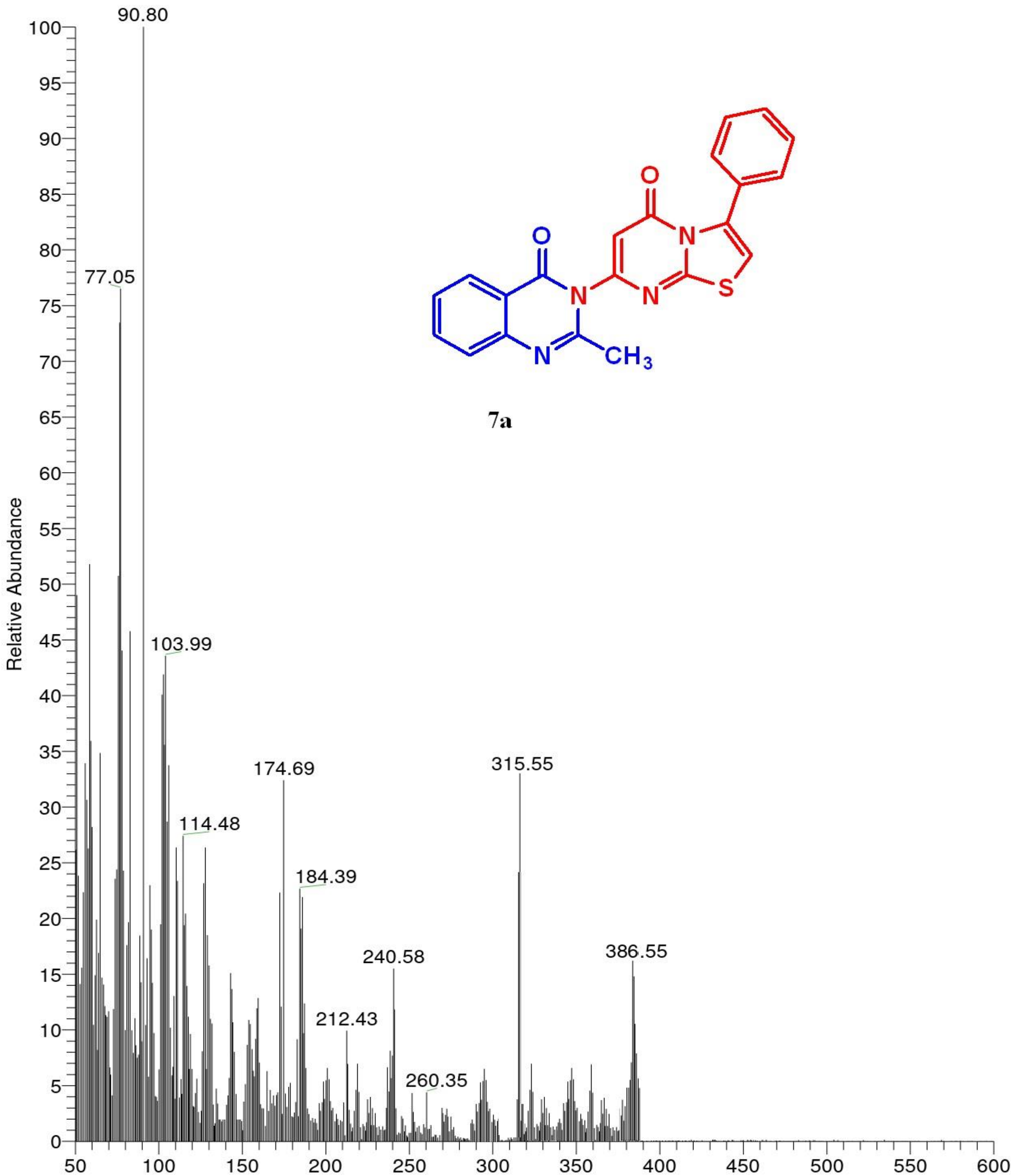

Mass spectra of compound **7a**

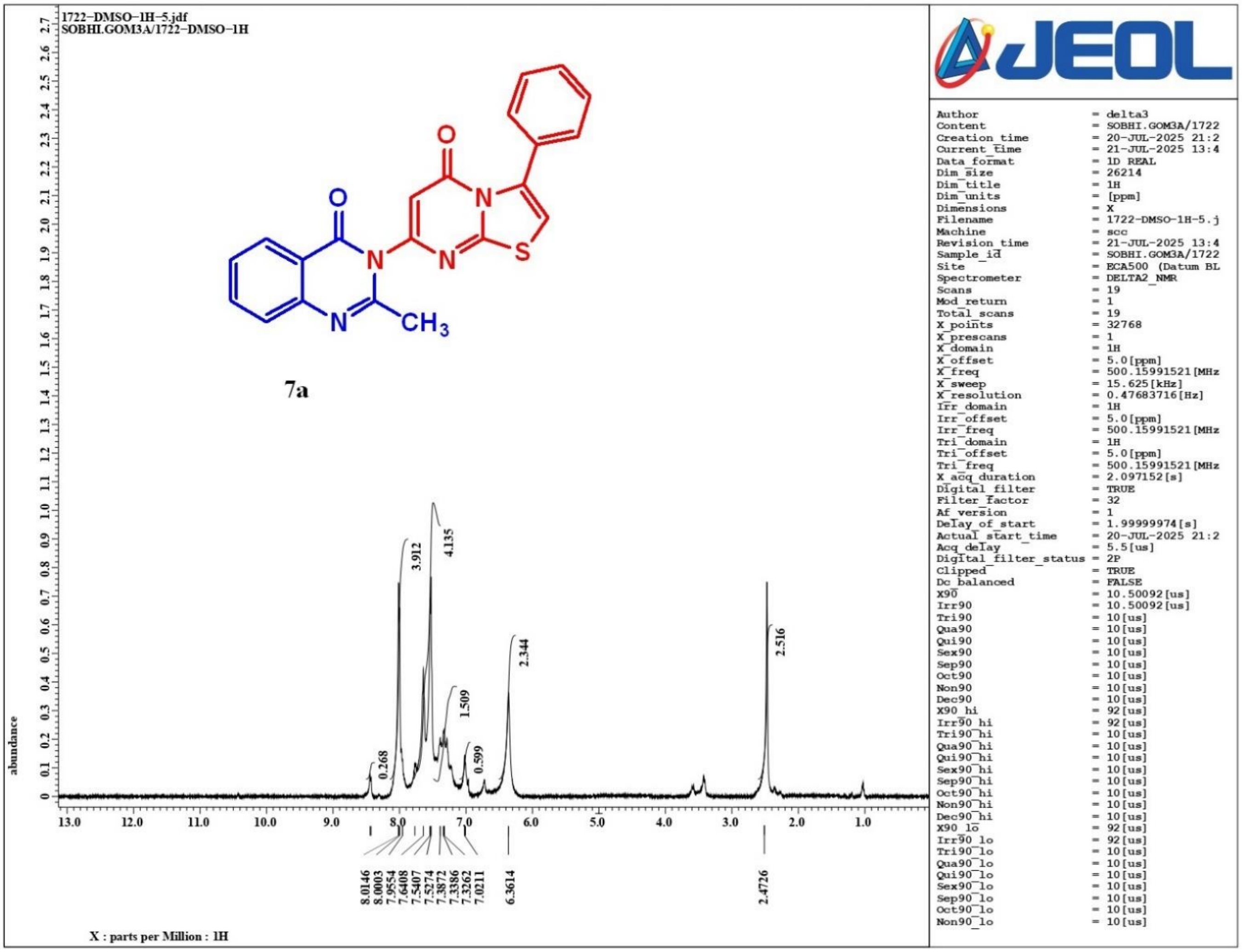

^{1}HNMR spectra of compound **7a**

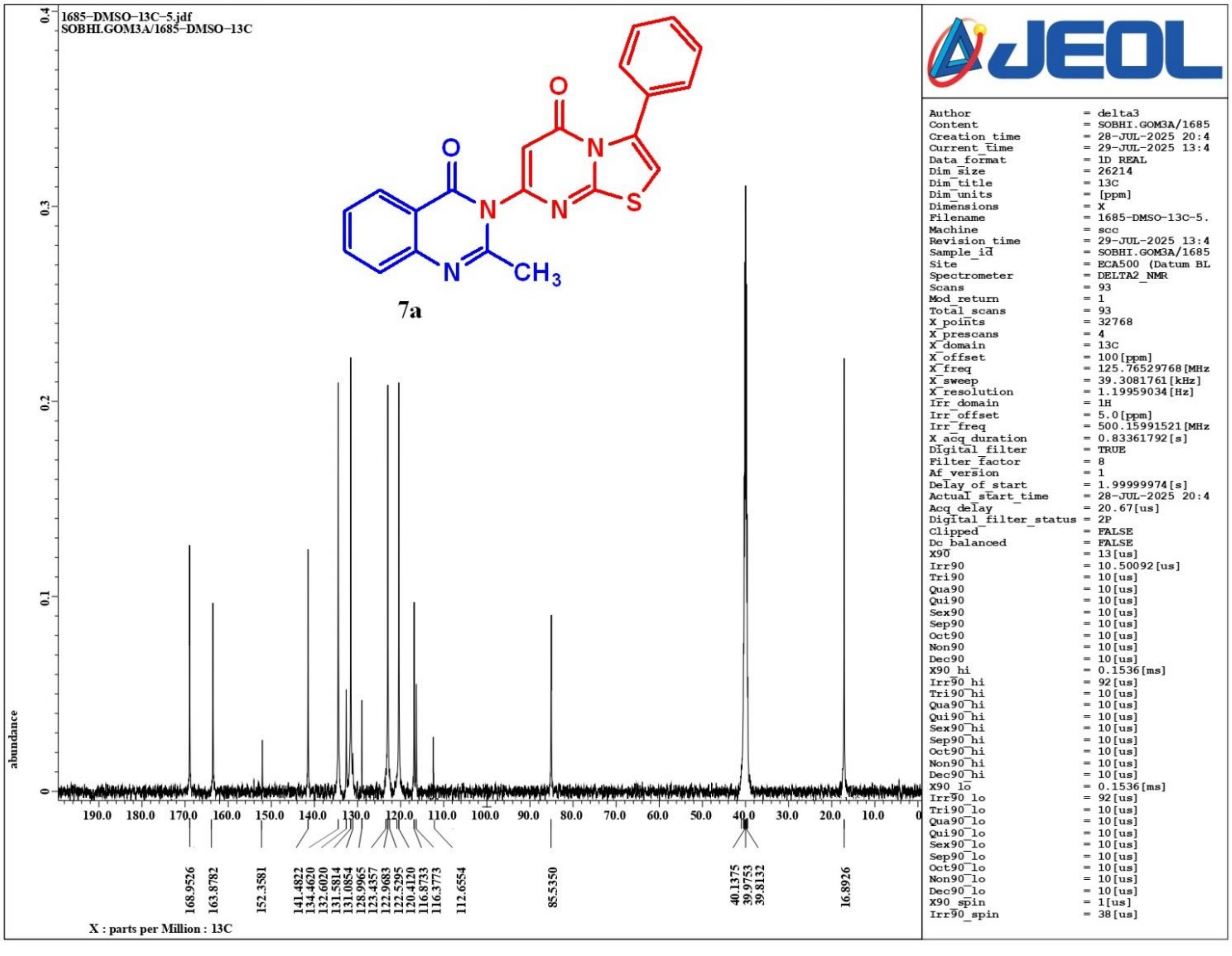

^{13}CNMR spectra of compound **7a**

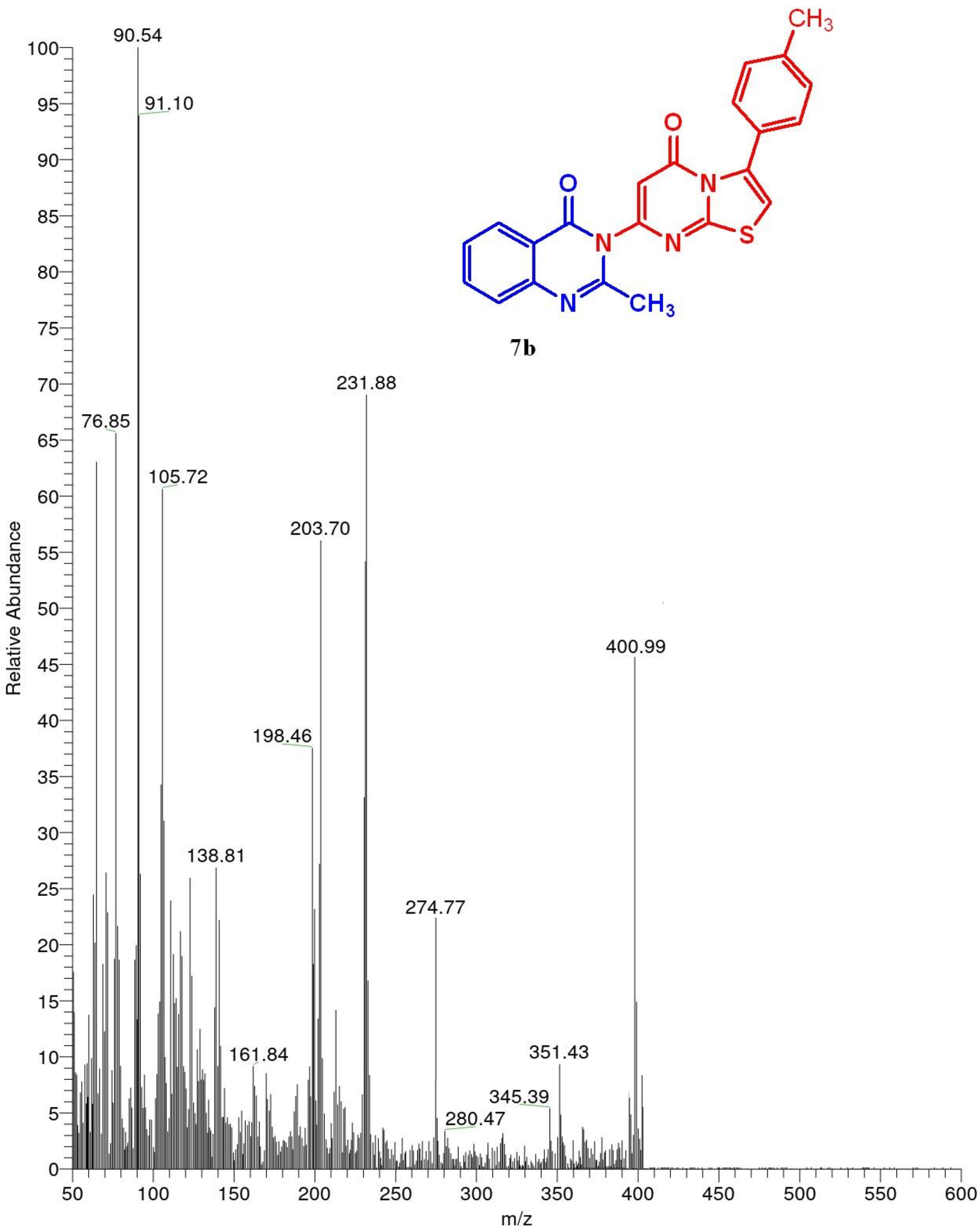

Mass spectra of compound **7b**

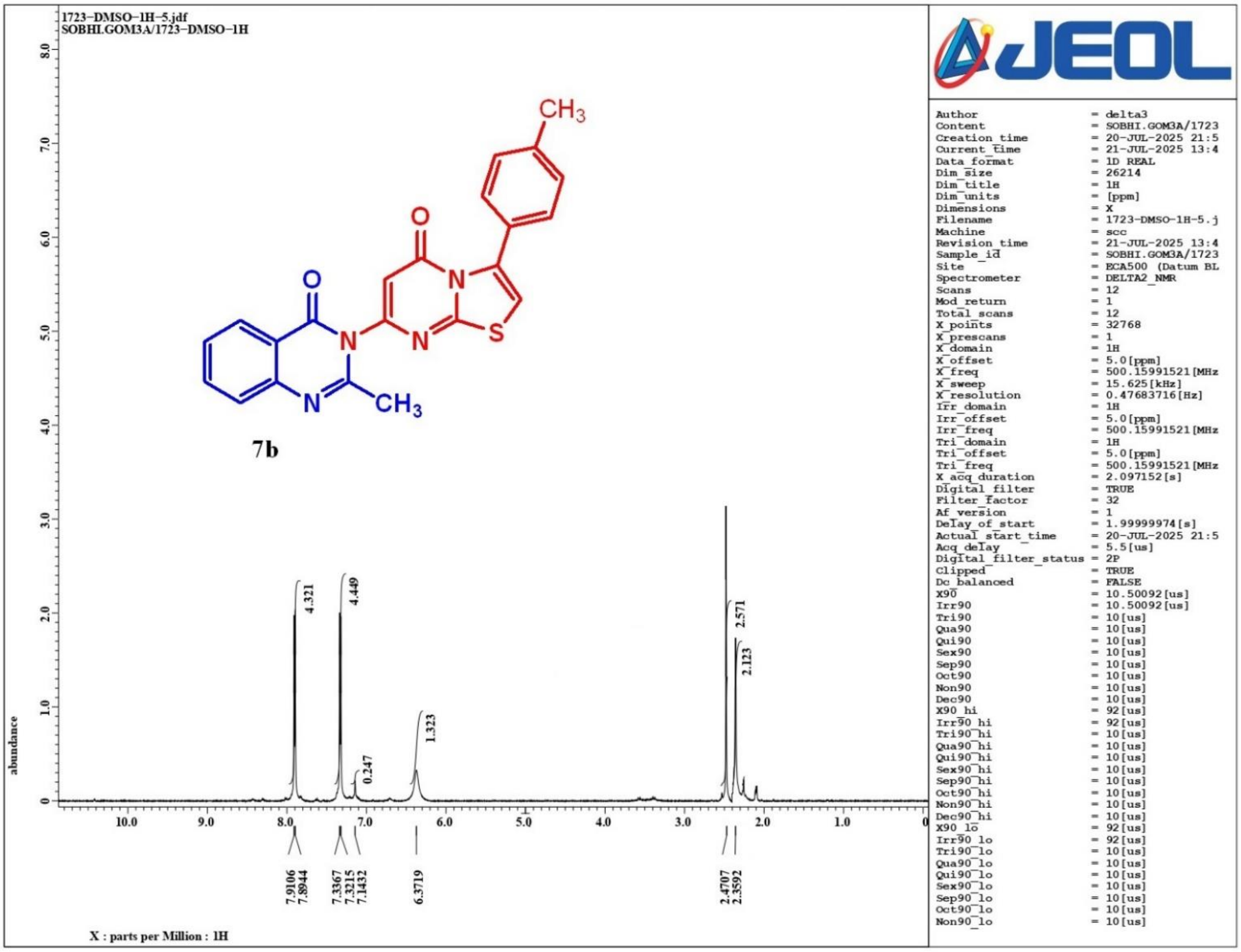

^{1}HNMR spectra of compound **7b**

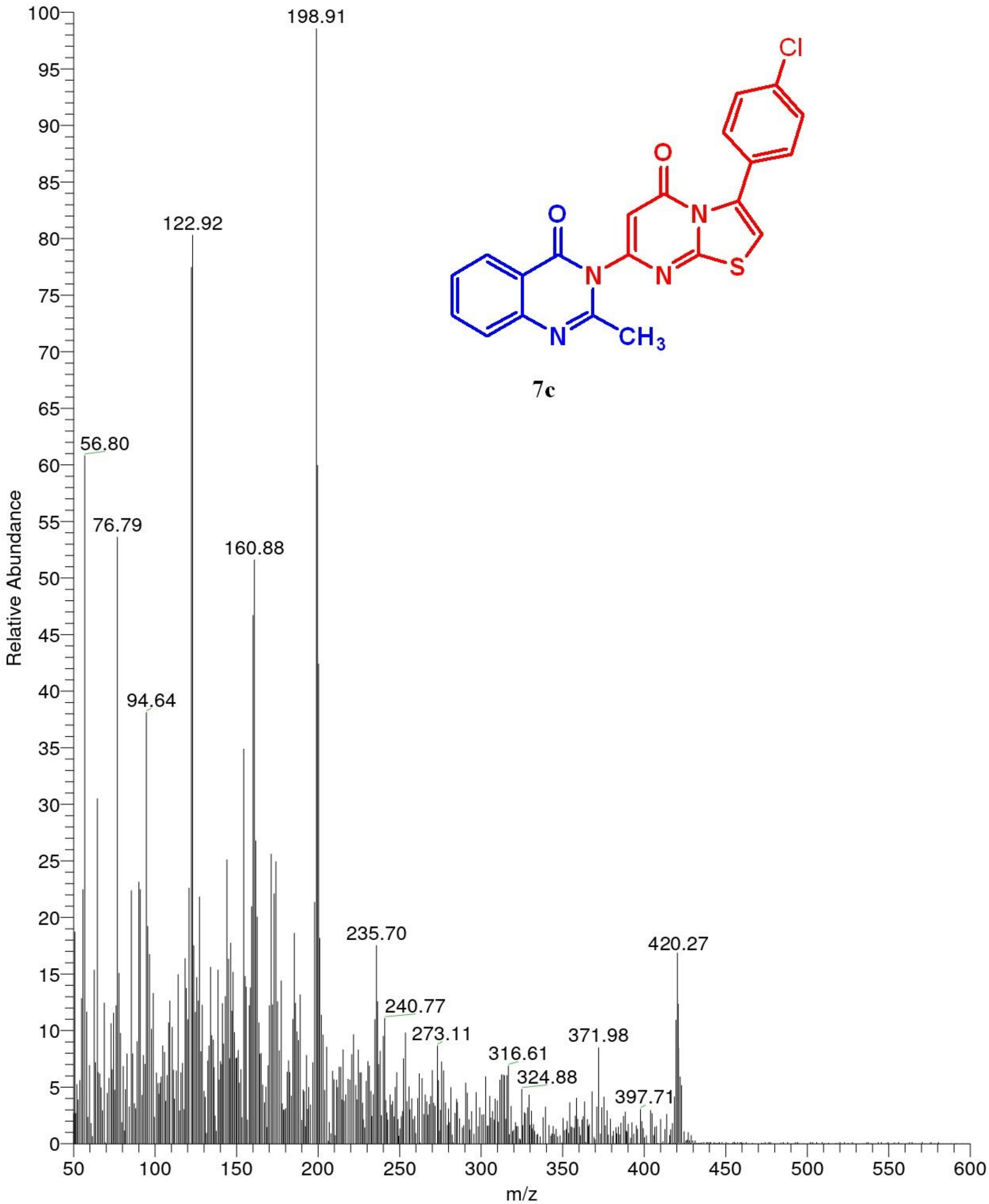

Mass spectra of compound **7c**

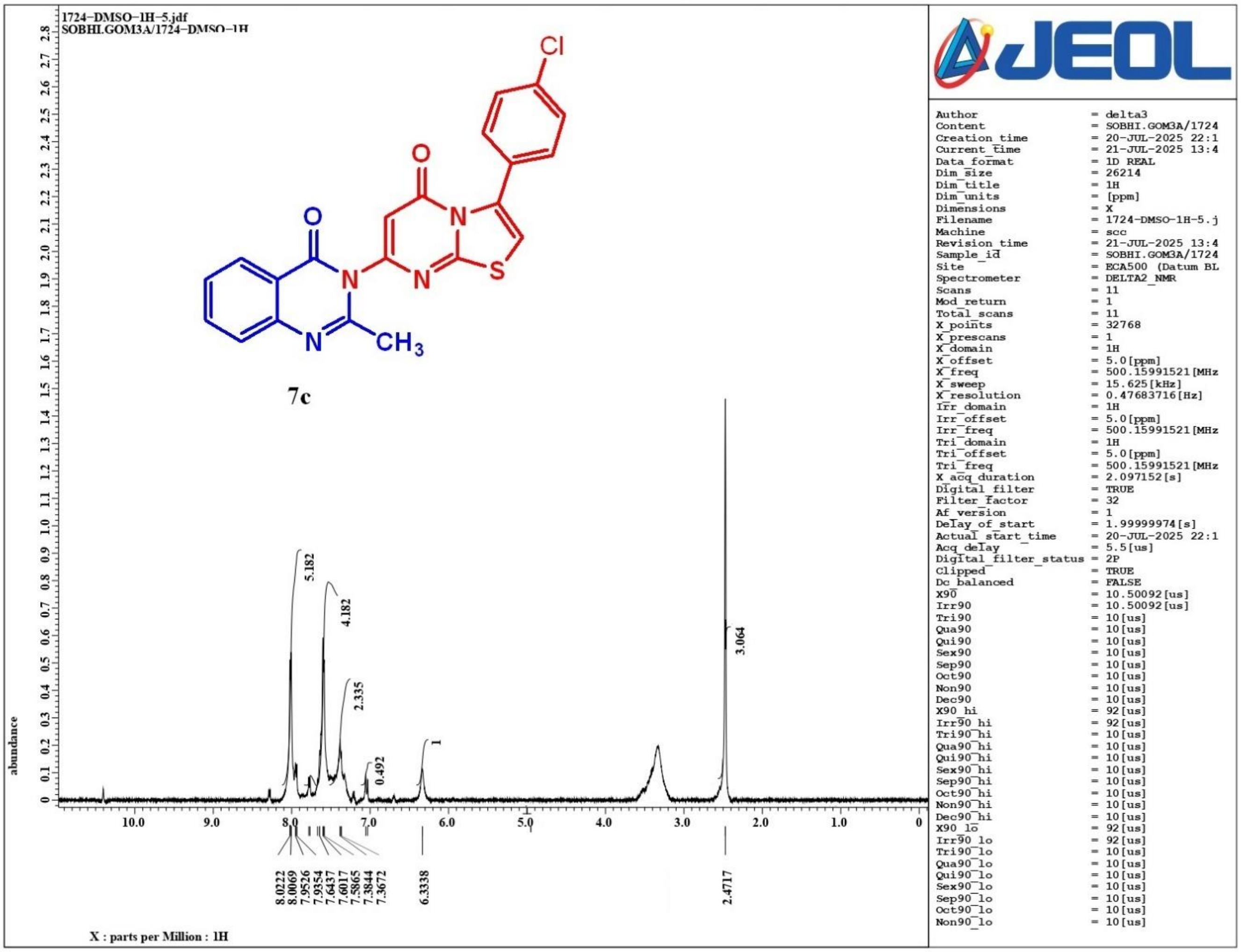

^{1}HNMR spectra of compound **7c**

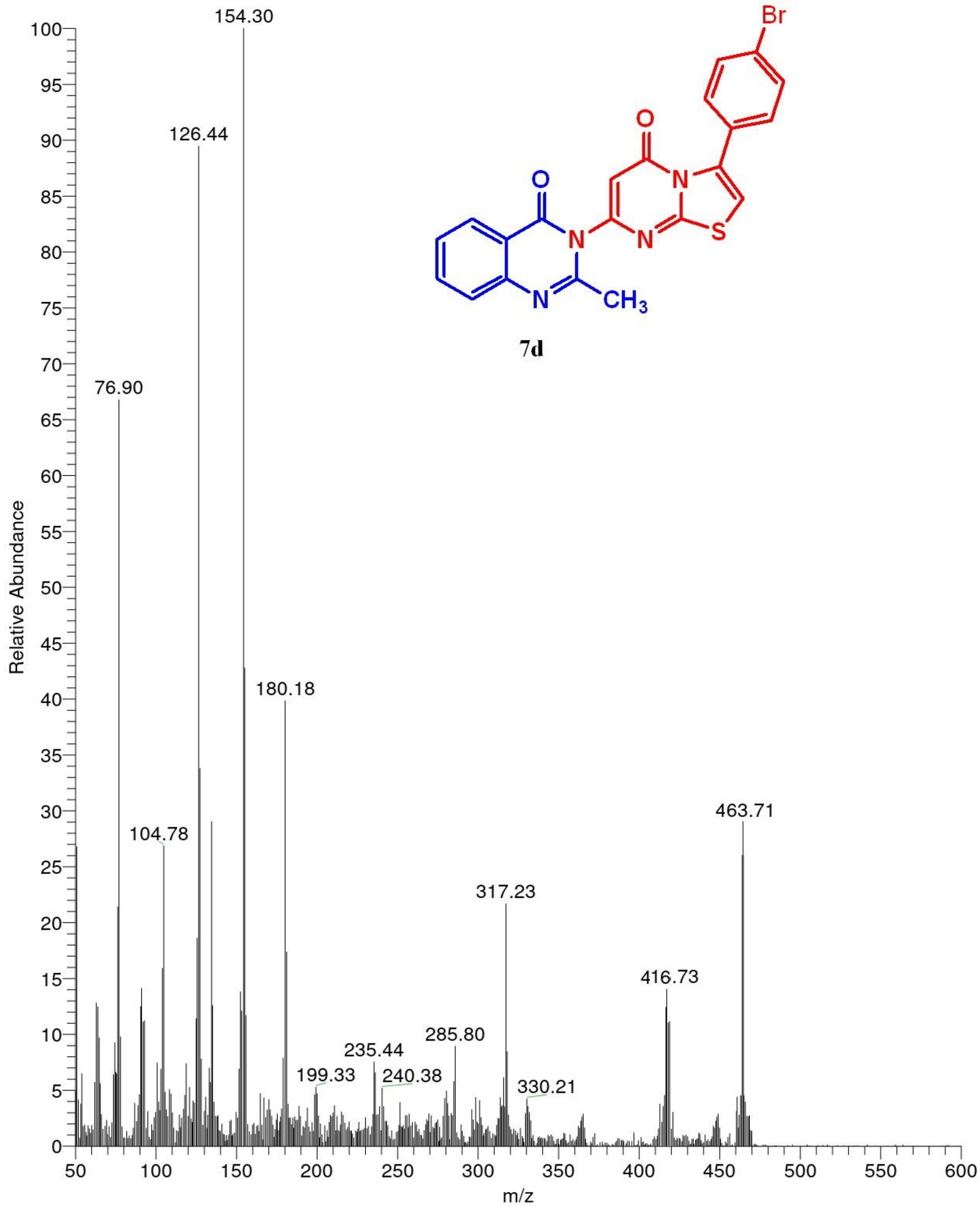

Mass spectra of compound **7d**

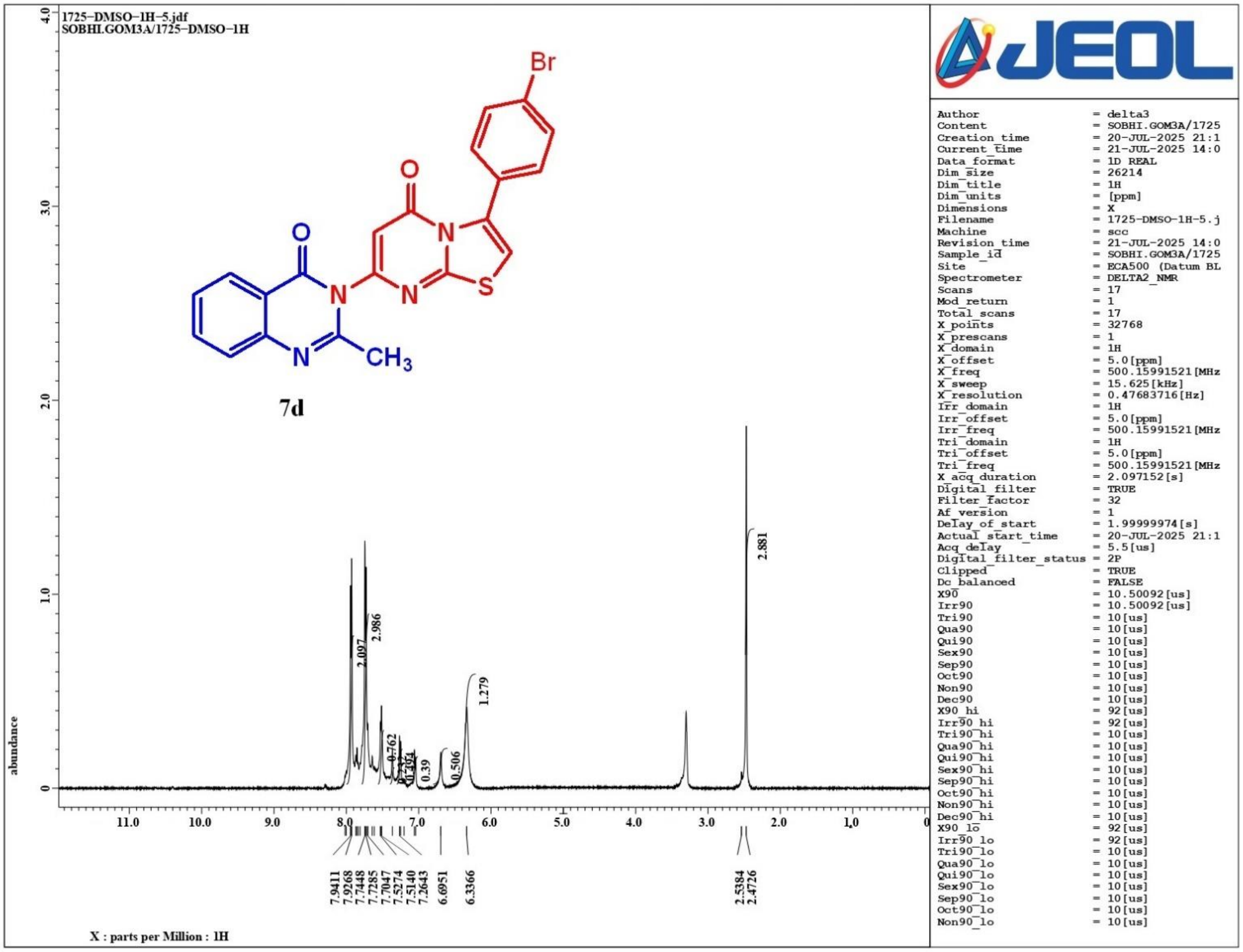

^{1}HNMR spectra of compound **7d**

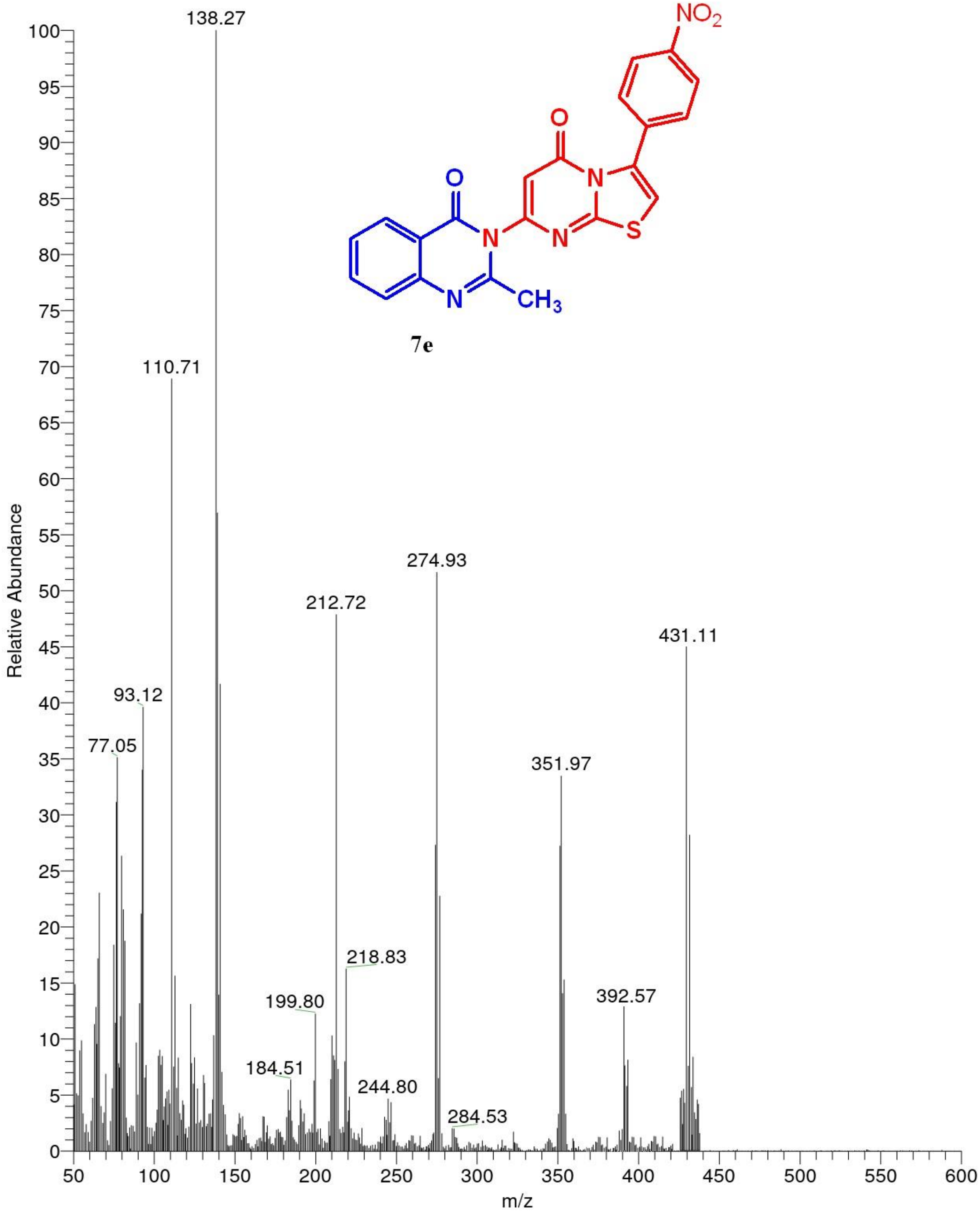

Mass spectra of compound **7e**

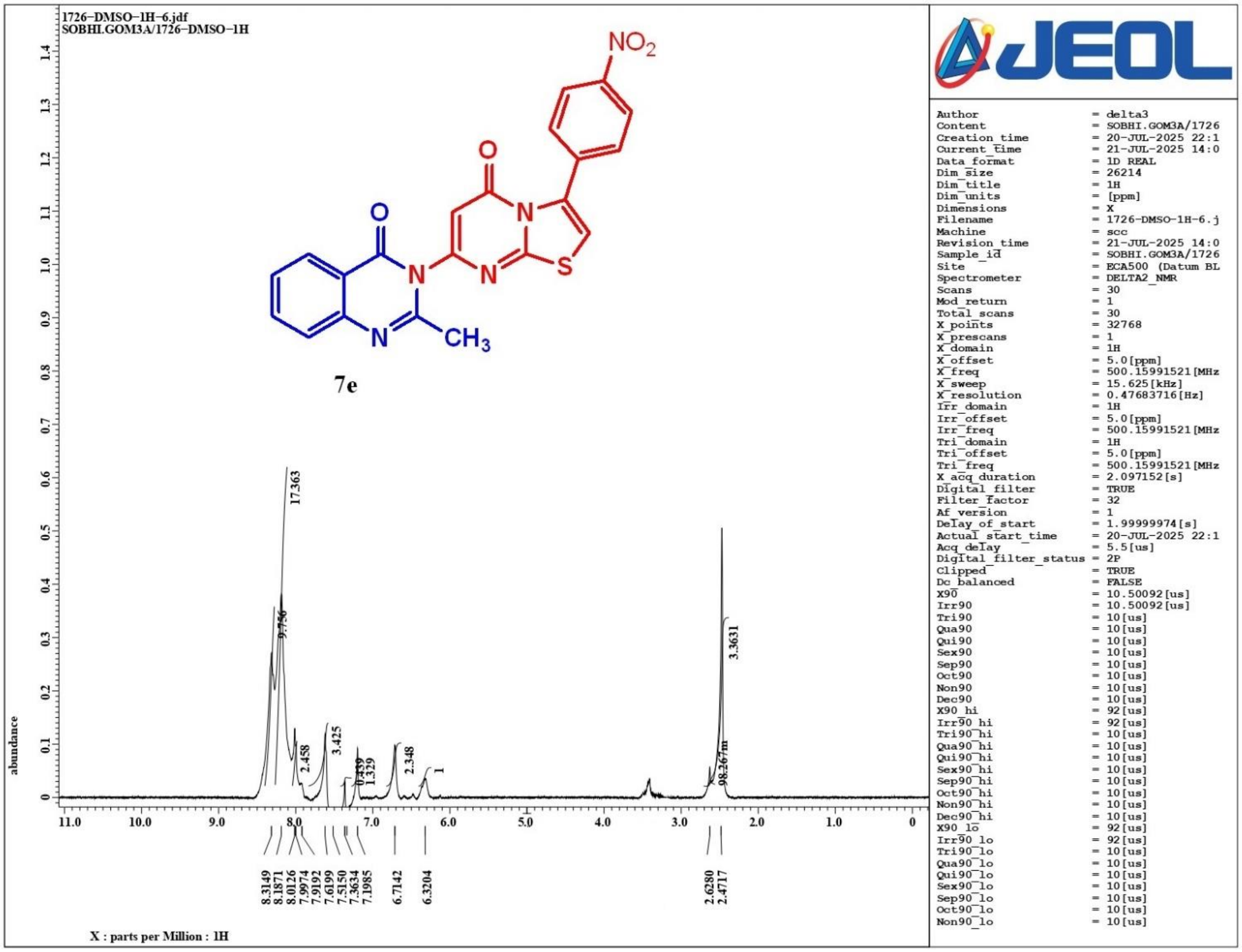

^{1}HNMR spectra of compound **7e**

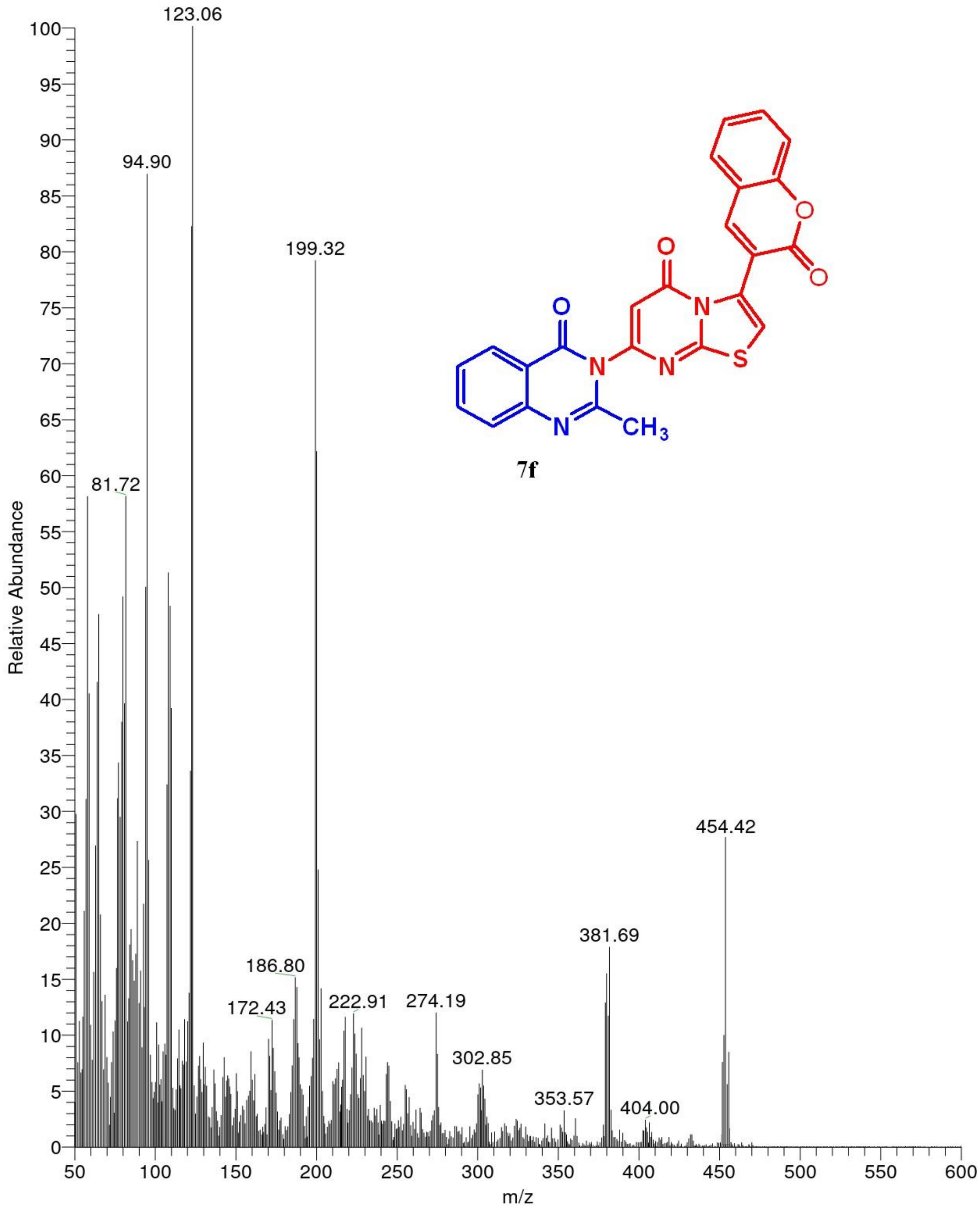

Mass spectra of compound **7f**

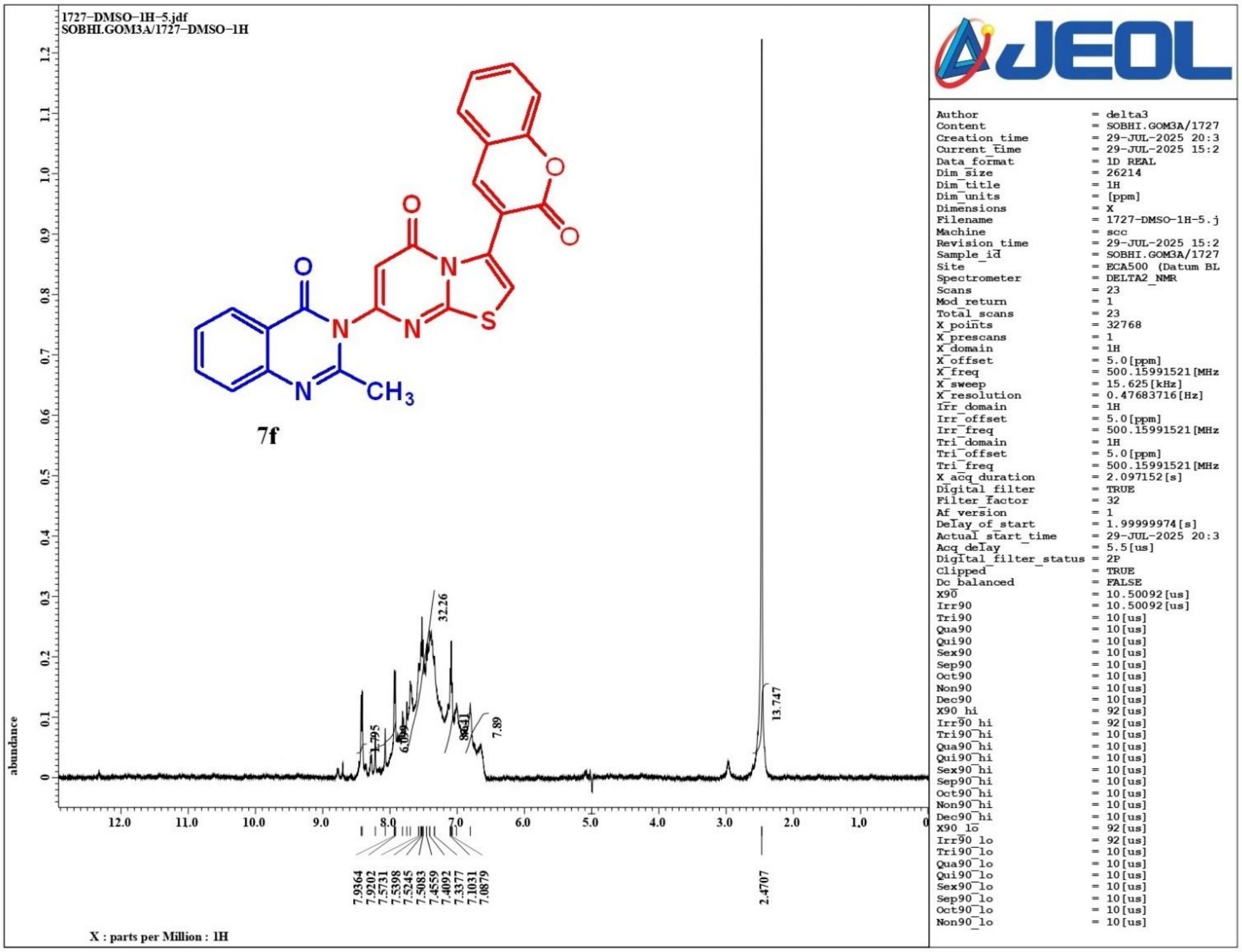

^{1}HNMR spectra of compound **7f**

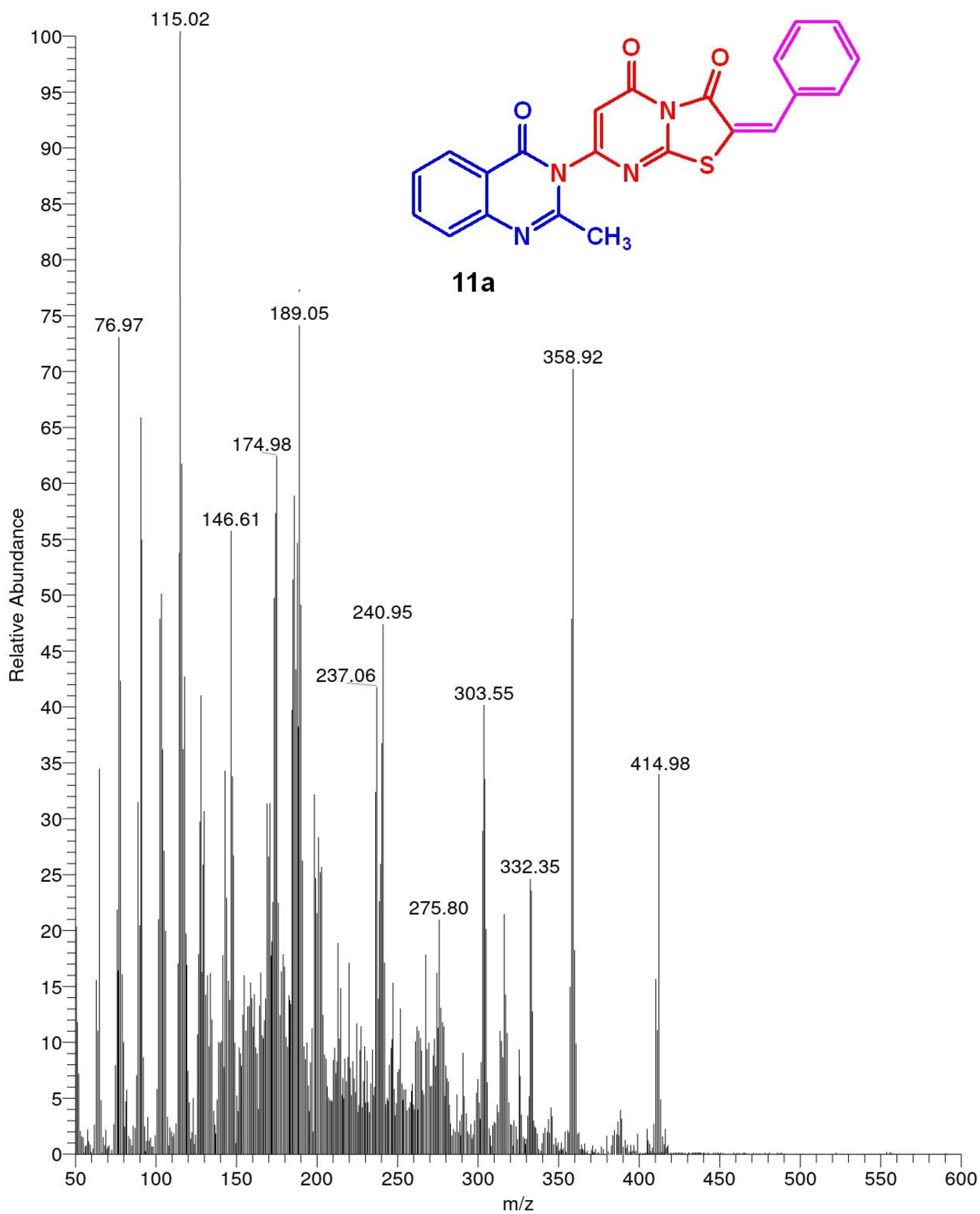

Mass spectra of compound **11a**

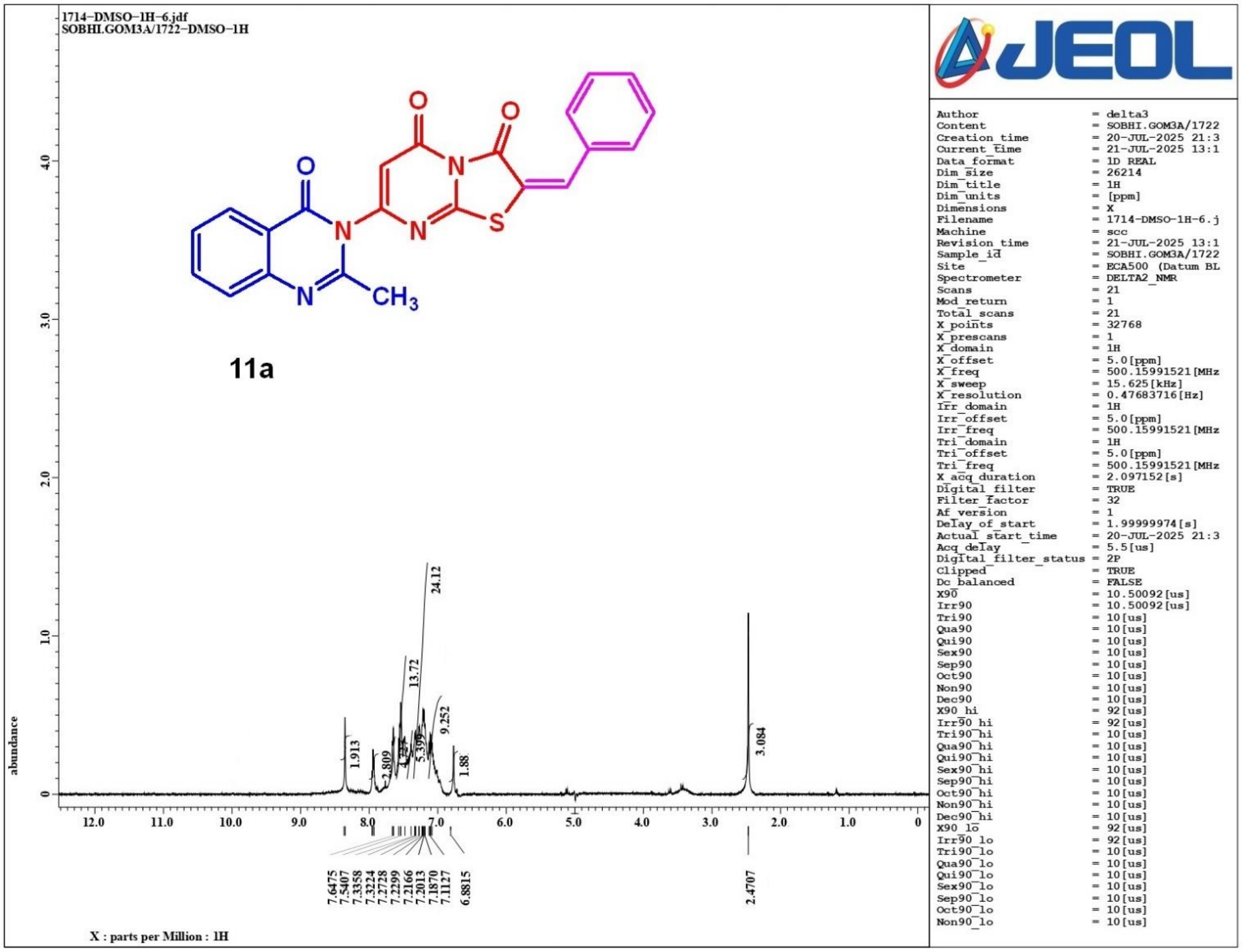

^{1}HNMR spectra of compound **11a**

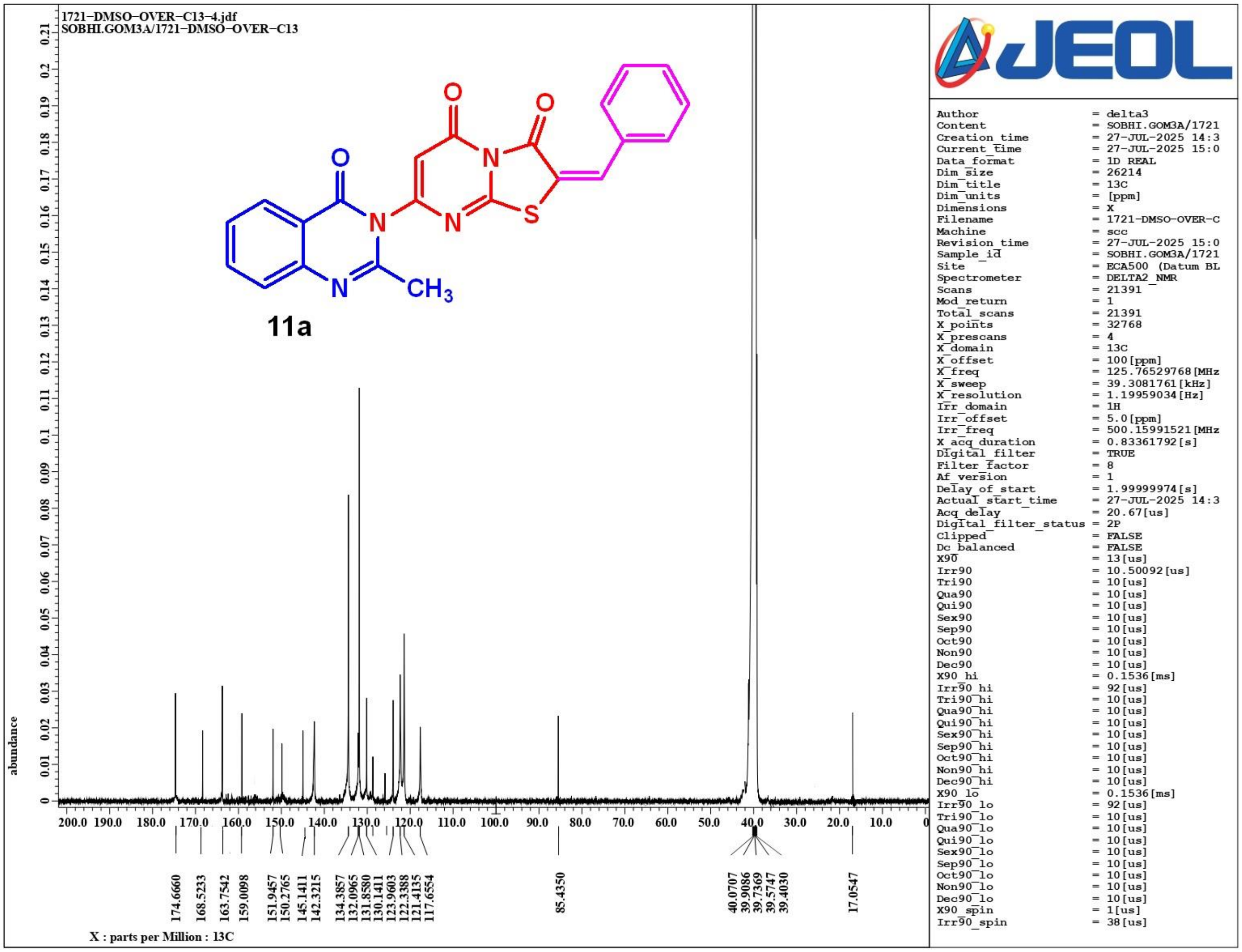

^{13}CNMR spectra of compound **11a**

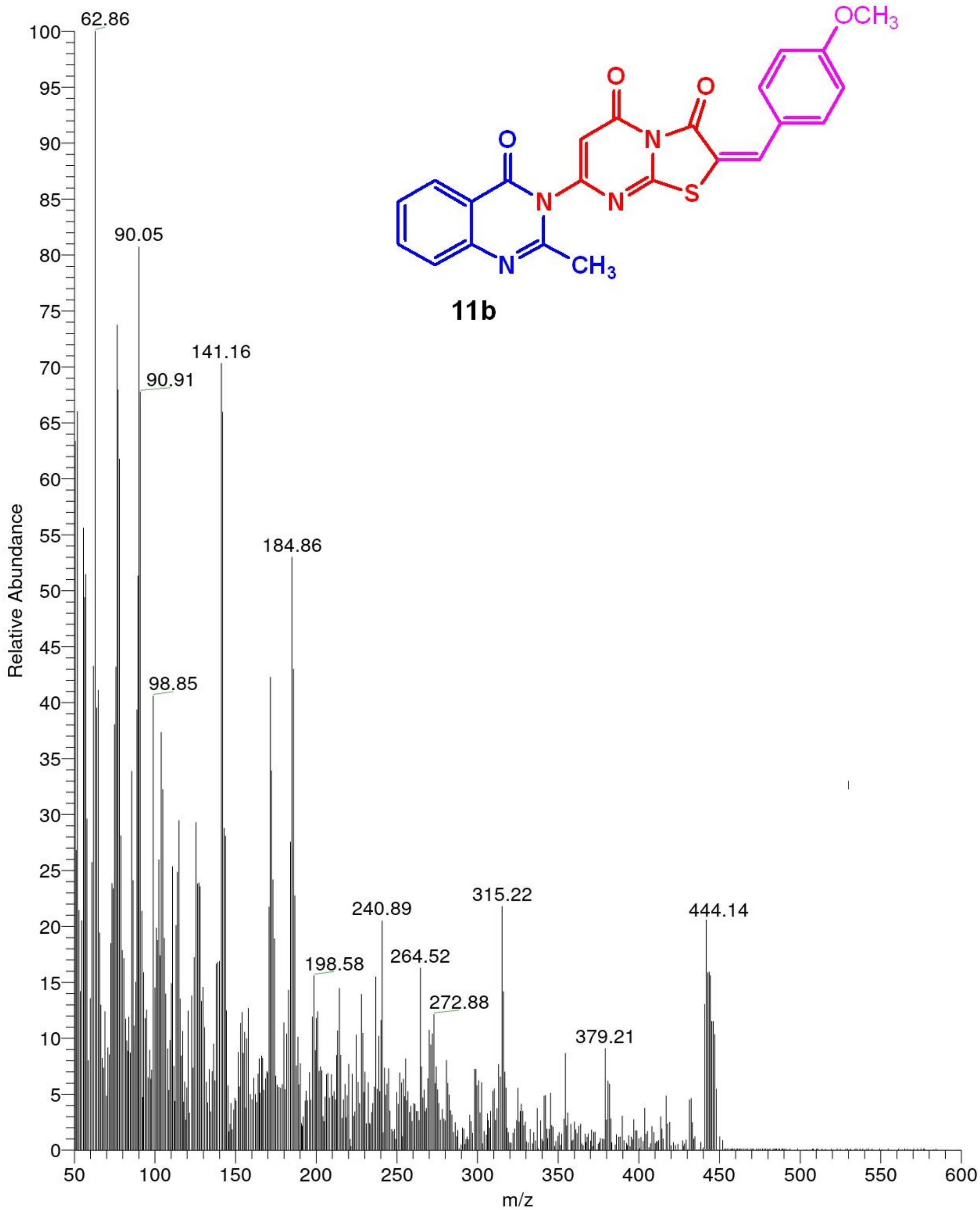

Mass spectra of compound **11b**

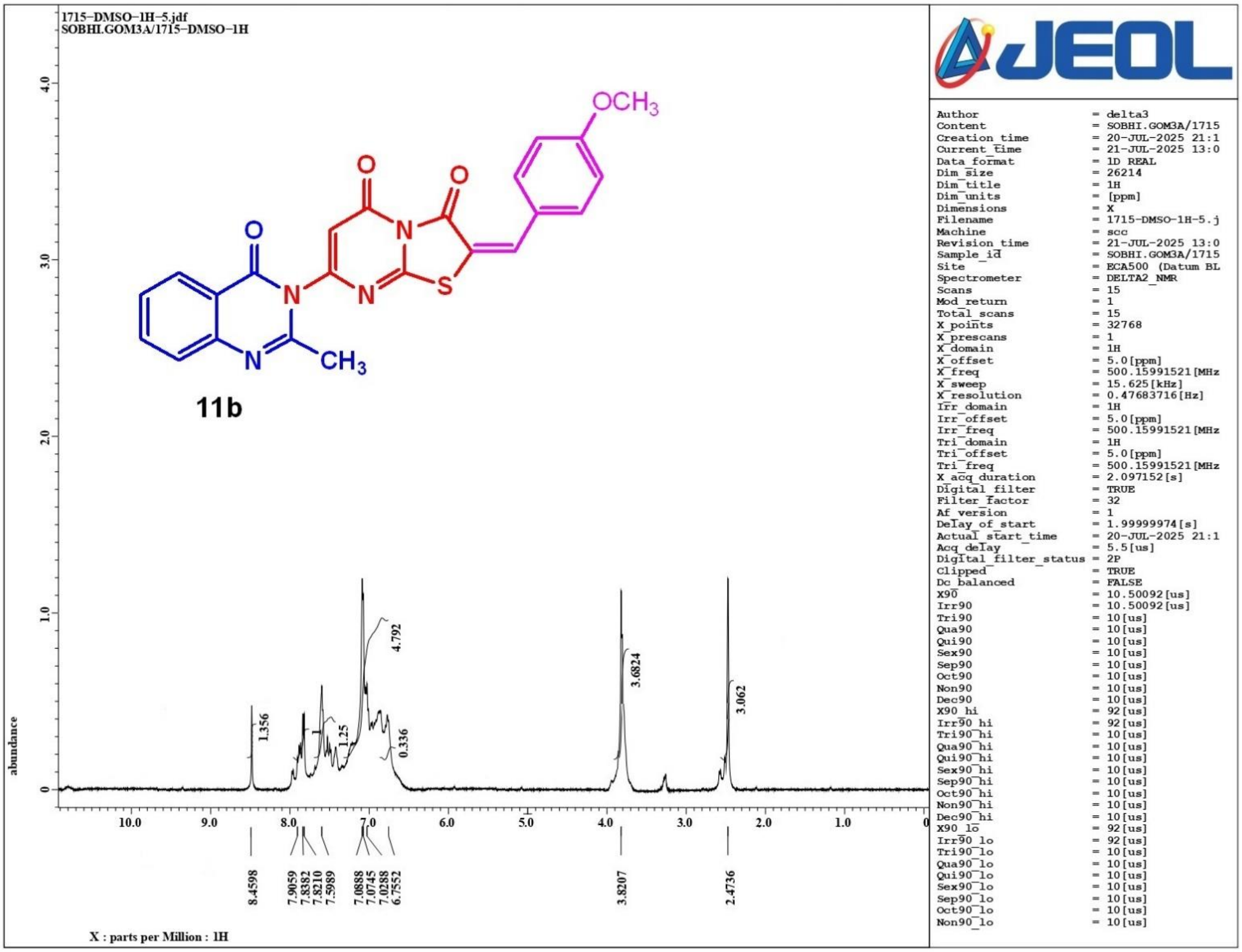

^{1}HNMR spectra of compound **11b**

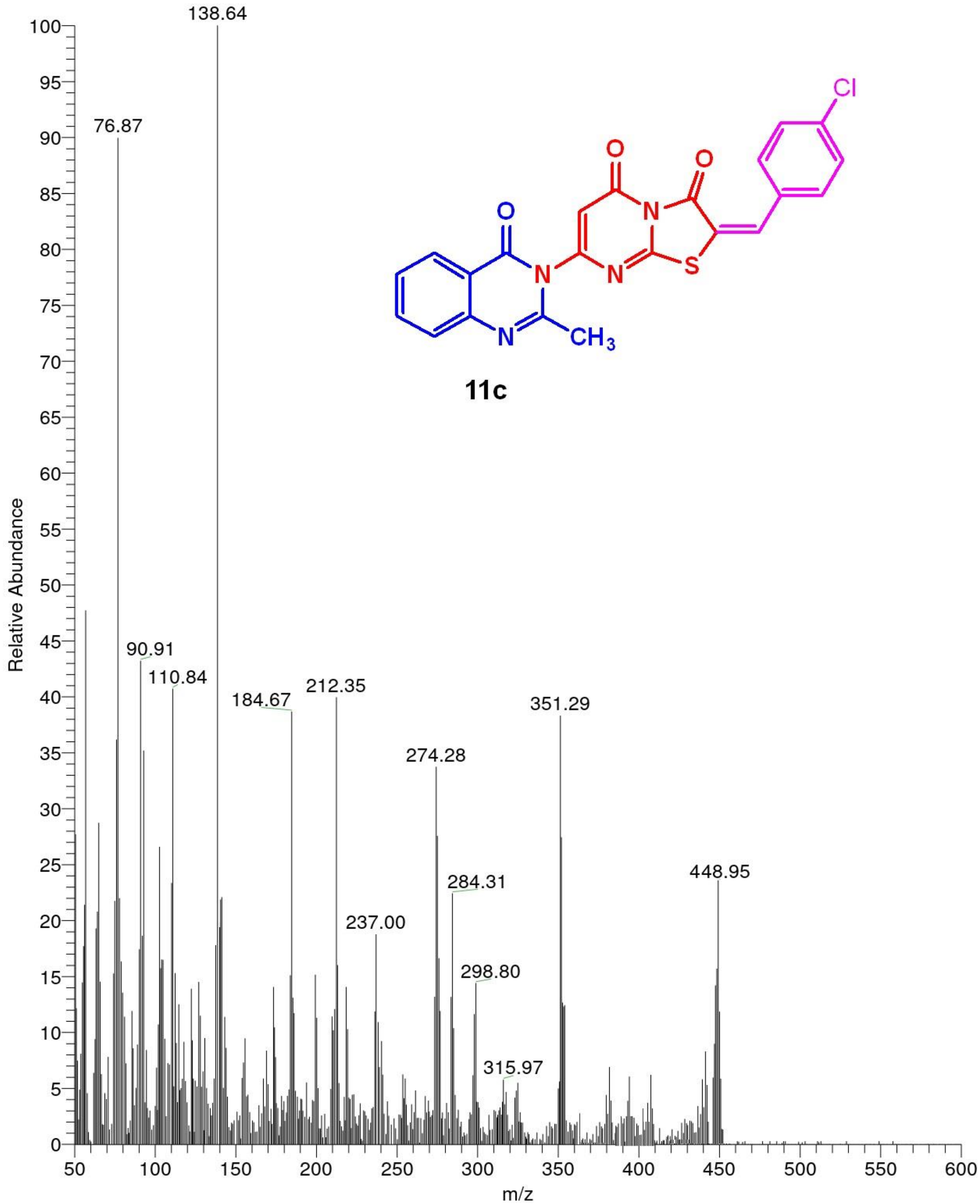

Mass spectra of compound **11c**

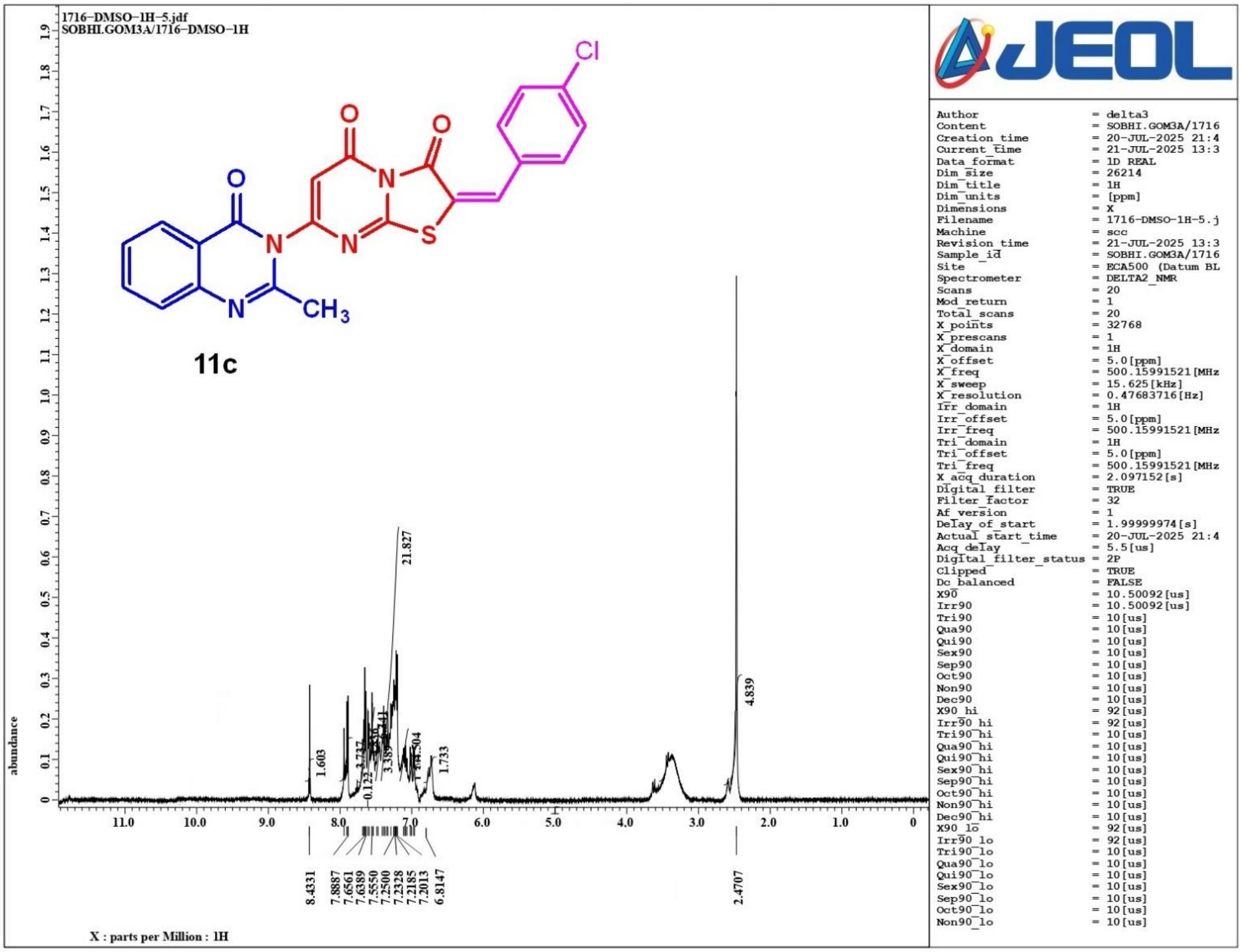

^{1}HNMR spectra of compound **11c**

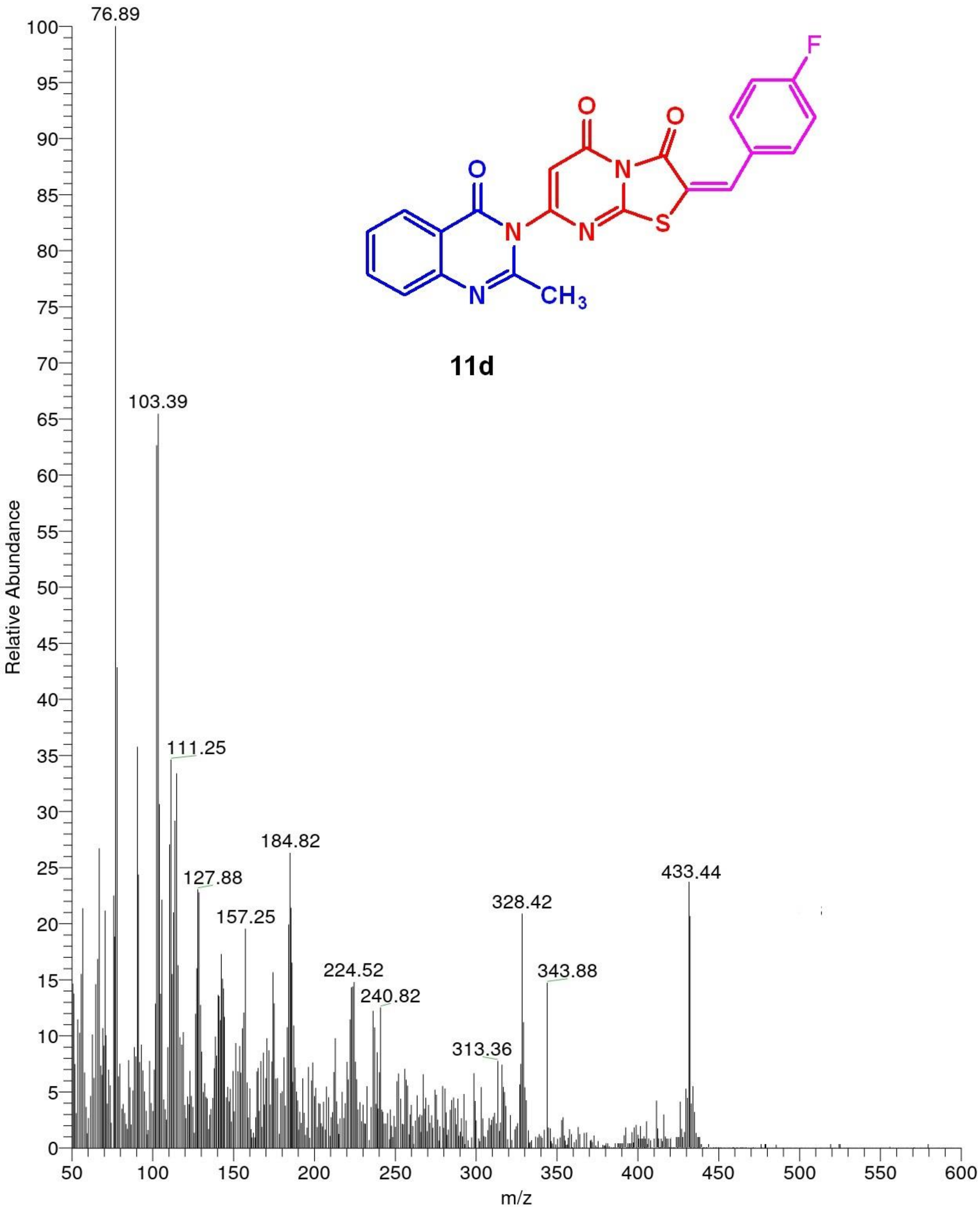

Mass spectra of compound **11d**

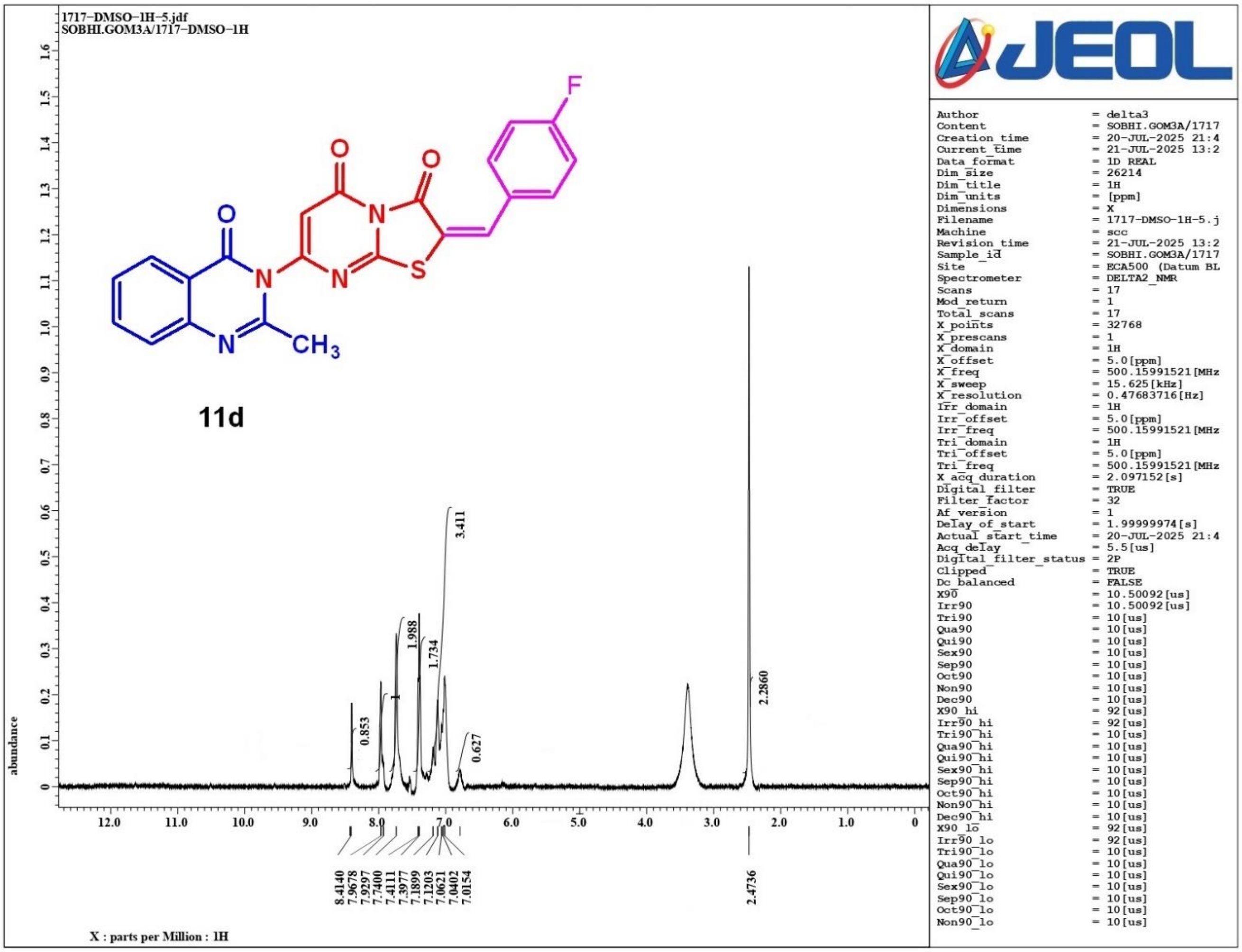

^{1}HNMR spectra of compound **11d**

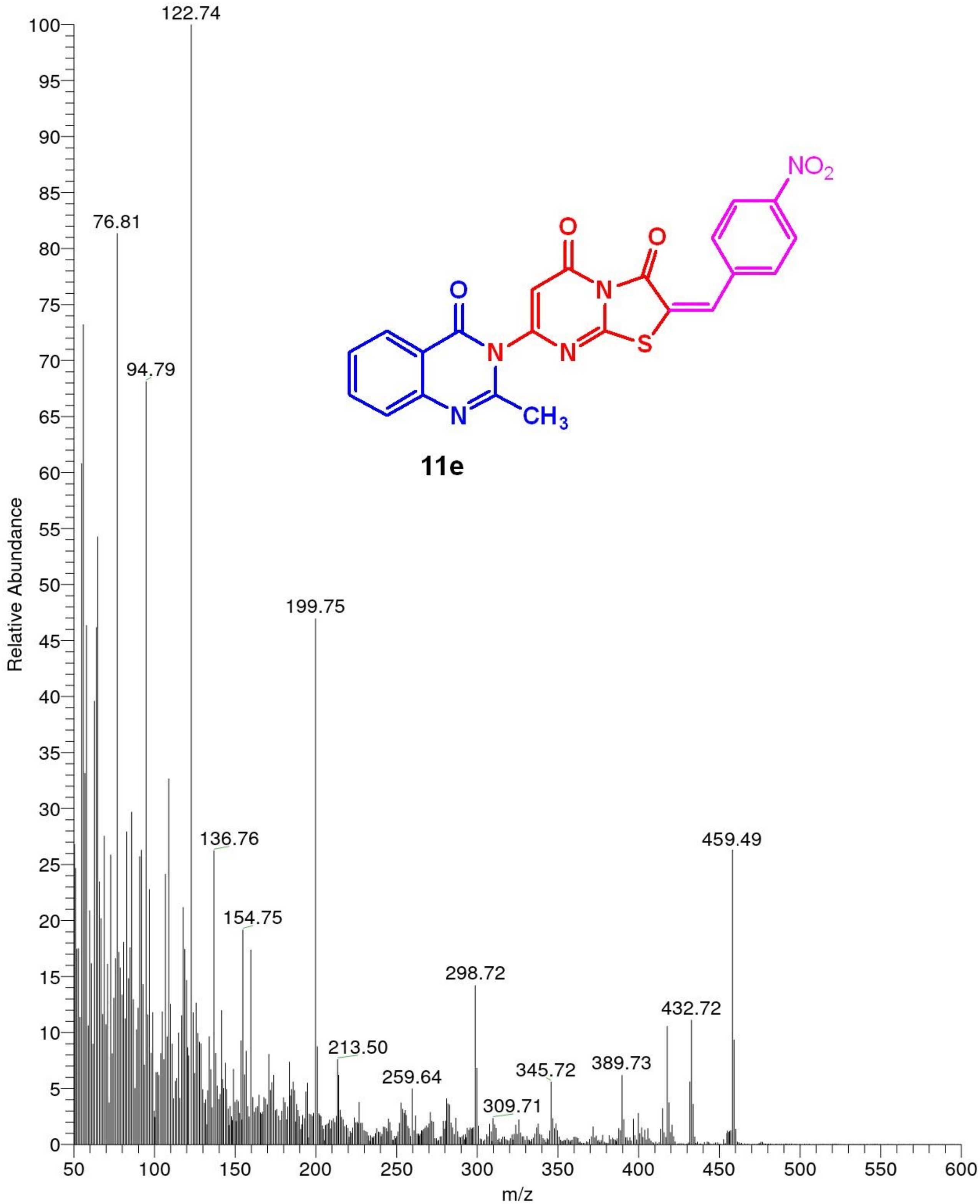

Mass spectra of compound **11e**

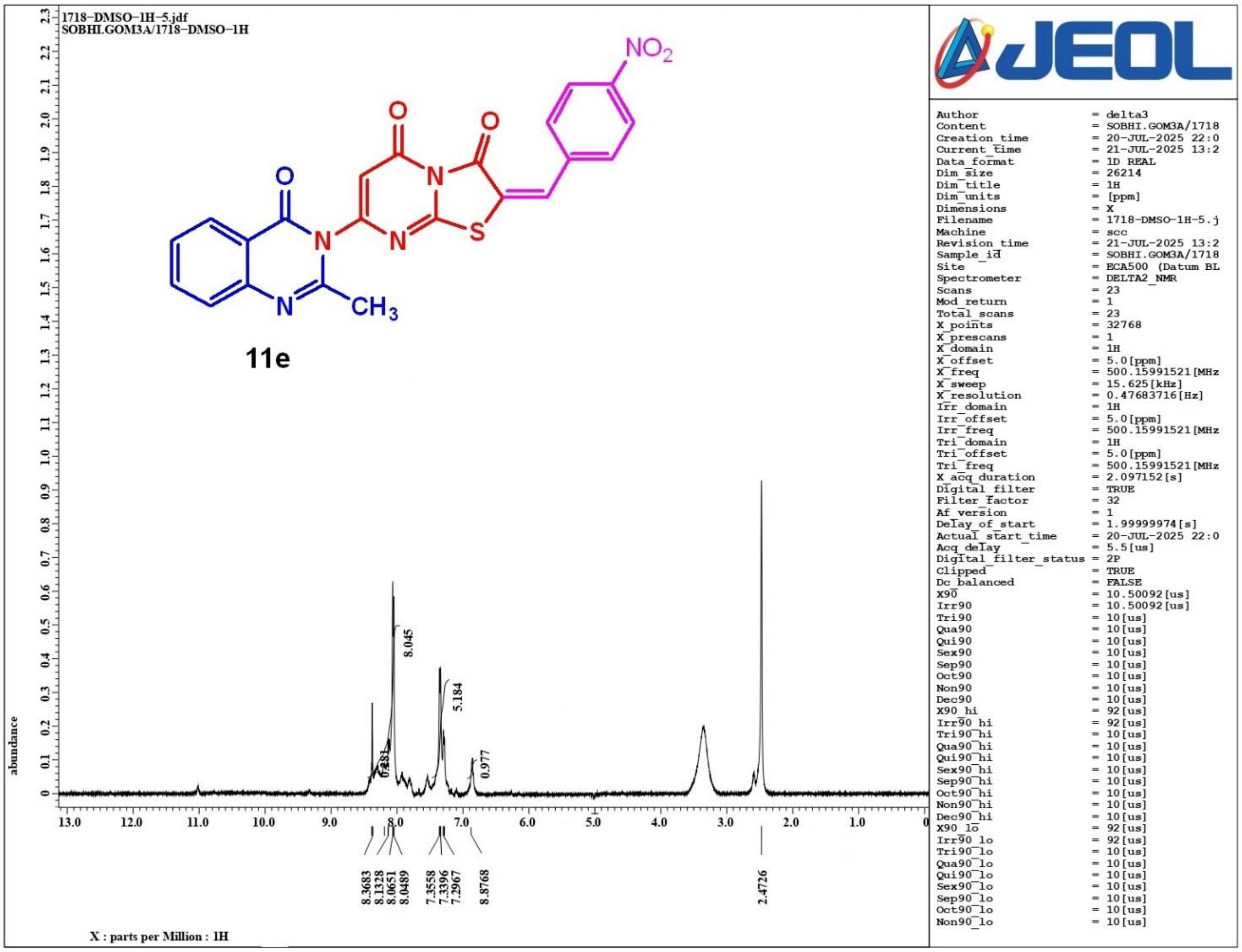

^{1}HNMR spectra of compound **11e**

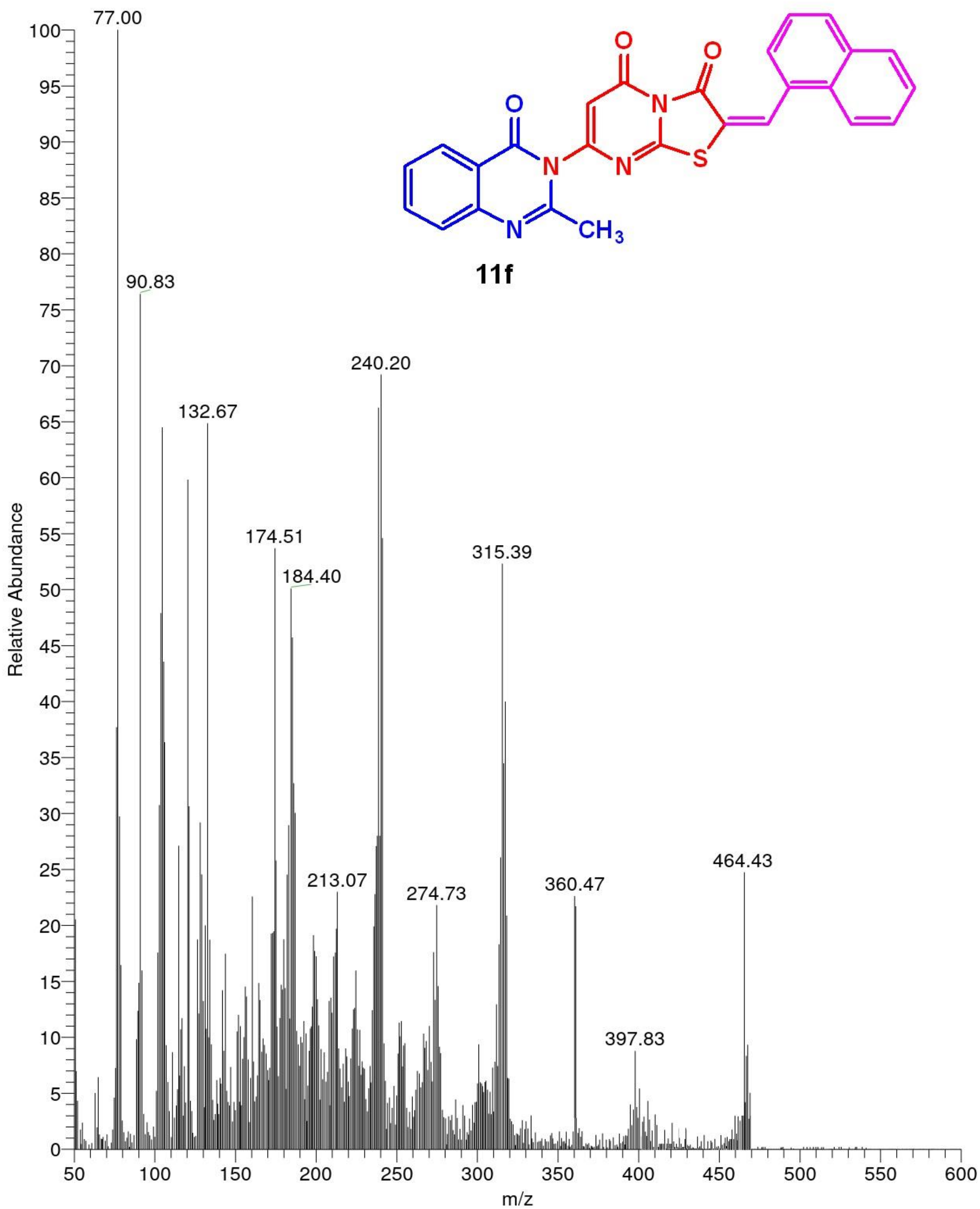

Mass spectra of compound **11f**

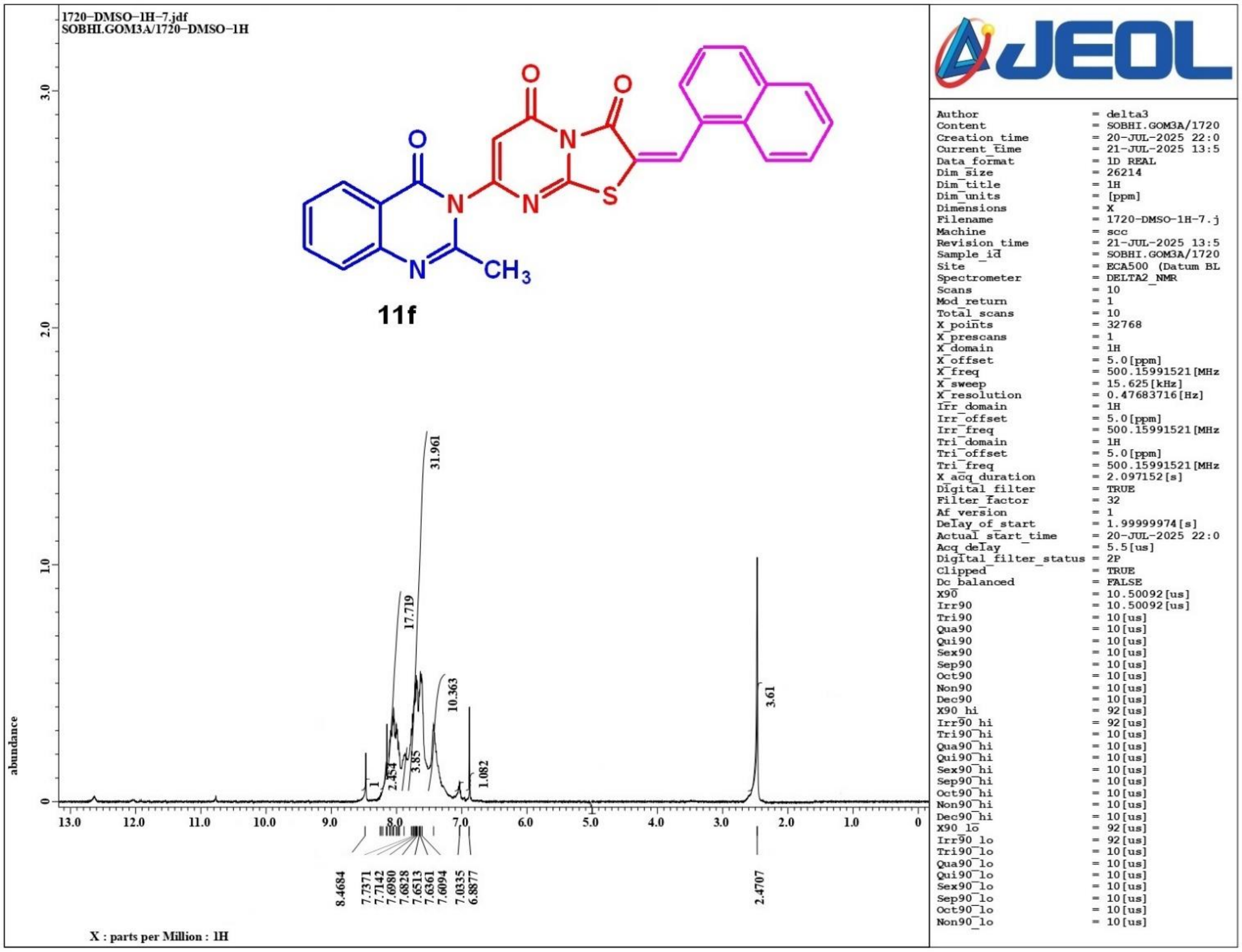

^{1}HNMR spectra of compound **11f**
